# Dataset on daytime outdoor thermal comfort for Belo Horizonte, Brazil

**DOI:** 10.1016/j.dib.2016.09.019

**Published:** 2016-09-20

**Authors:** Simone Queiroz da Silveira Hirashima, Eleonora Sad de Assis, Marialena Nikolopoulou

**Affiliations:** a Department of Civil Engineering, Federal Center of Technological Education of Minas Gerais, Av. Amazonas, 7675, CEP 30510-000 Belo Horizonte, Minas Gerais, Brasil; b Department of Technology of Architecture and Urbanism, The Federal University of Minas Gerais, Rua Paraíba, 697, CEP 30130-140 Belo Horizonte, Minas Gerais, Brasil; c Kent School of Architecture, Marlowe Building, University of Kent, Canterbury, Kent CT2 7NR, United Kingdom

## Abstract

This dataset describe microclimatic parameters of two urban open public spaces in the city of Belo Horizonte, Brazil; physiological equivalent temperature (PET) index values and the related subjective responses of interviewees regarding thermal sensation perception and preference and thermal comfort evaluation. Individuals and behavioral characteristics of respondents were also presented. Data were collected at daytime, in summer and winter, 2013. Statistical treatment of this data was firstly presented in a PhD Thesis (“Percepção sonora e térmica e avaliação de conforto em espaços urbanos abertos do município de Belo Horizonte – MG, Brasil” (Hirashima, 2014) [1]), providing relevant information on thermal conditions in these locations and on thermal comfort assessment. Up to now, this data was also explored in the article “Daytime Thermal Comfort in Urban Spaces: A Field Study in Brazil” (Hirashima et al., in press) [2]. These references are recommended for further interpretation and discussion.

**Specifications Table**

**Table:** 

Subject area	*Architecture and Urban Planning*
More specific subject area	*Outdoor Thermal Comfort*
Type of data	*Figure, table*
How data was acquired	*Data was acquired through field surveys. Objective environmental parameters were collected through measurements using instruments (thermo hygrometer make HOBO, model U12; thermo anemometer make ALNOR, model CF8586; globe thermometers, assembled by the Federal University of Minas Gerais using a thermo hygrometer make HOBO, model U12 (external channel), a temperature sensor make National Semiconductor, model LM35DT/NOPB and a table tennis ball painted in grey*[3]*; and a sensors shelter assembled with PVC pieces*[3]). *Measurement procedures were in accordance with ISO 7726*[4]*. Individual and Behavioral’ variables and subjective responses were collected through structured interviews and observations. The questionnaire was elaborated in accordance with ISO 10551*[5]*, ISO 7730*[6]*, ISO 8996*[7]*and ISO 9920*[8].
Data format	*Raw*
Experimental factors	*The sample frame considered the adult population (20–59 years-old) residing in Belo Horizonte for more than one year and that was in outdoors environments for more than 30 min. Field surveys were conducted on sunny days (no rain)*[1].
Experimental features	*Data collections occurred at Liberdade Square and at Sete de Setembro Square, in summer and in winter, Belo Horizonte, Brazil. Field surveys were conducted from 7.00 a.m. to 5.00 p.m., one day each area in each season. Microclimatic data were recorded every 5 min*[1].
Data source location	*Belo Horizonte, (19*°*55*′*S, 43*°*56*′*W), Brazil.*
Data accessibility	*Data are with this article.*

**Value of the data**

• This data can help researchers and practice professionals to evaluate the outdoors thermal conditions and can be used in the development of further experiments and/or future architectural intervention in these particular areas.
• Statistical treatment of this data may allow the comparison of thermal comfort conditions people experience in different or in similar climatic and/or cultural contexts.
• Statistical treatment of this data may allow the identification of potential thermal adaptation processes of population in different or in similar climatic and/or cultural contexts.

## 1 Data

This article contains a dataset on microclimatic variable, on values of the PET index, on subjective responses concerning perception of thermal sensation, thermal comfort evaluation and preferences of thermal sensation, and on individual and behavioral’ characteristics of interviewees.

## 2 Experimental design, materials and methods

### 2.1 Study areas and measurement points

**Fig. 1:**
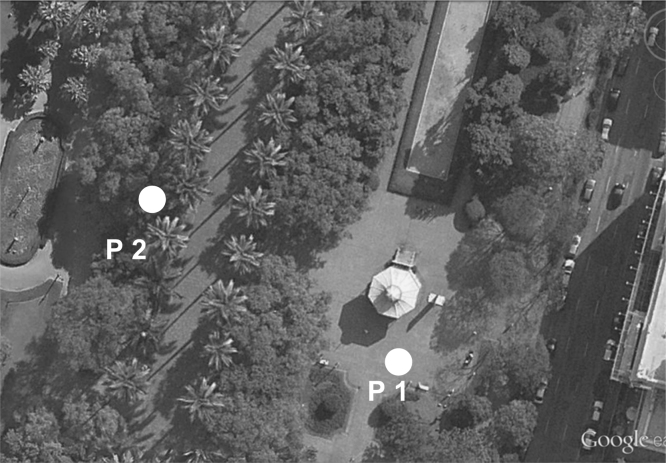
Location of Point 1 (P1) and Point 2 (P2) in Liberdade Square, in the sun and in the shade, respectively. Source of the image: GOOGLE EARTH, [200–]. Imagem adapted by authors to composition of the figure, 2012 [1].

**Fig. 2:**
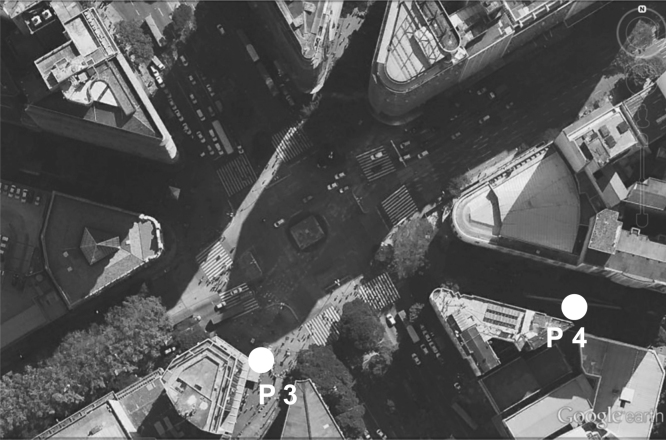
Location of Point 3 (P3) and Point 4 (P4) in Sete de Setembro Square, in the sun and in the shade, respectively. Source of the image: GOOGLE EARTH, [200–]. Imagem adapted by authors to composition of the figure, 2012 [1].

Data were collected in Liberdade square (see Fig. 1 in Ref. [2]) and Sete de Setembro square (see Fig. 2 in Ref. [2]). Further information on the climate of Belo Horizonte and on the urban characteristics of the squares, such as building height, vegetation indexes, permeable areas quantification and so on, is presented in [1]. In each square, two points were selected (one in the sun and another in the shade) for the measurements and for the administration of the questionnaires. Point 1 (P1), in Liberdade Square, and Point 3 (P3), in Sete de Setembro square are positioned in the sun, while Point 2 (P2), in Liberdade Square, and Point 4 (P4), in Sete de Setembro square are positioned in the shade (Fig. 1, Fig. 2).

### 2.2 Physical measurements and the calculation of Tmrt and of the PET index

The summer data collection was carried out in March (on 11th March 2013 at Liberdade square, and on 13th March 2013 at Sete de Setembro square), and the winter data collection occurred in July (on 08th July 2013 at Liberdade square, and on 09th July 2013 at Sete de Setembro square). Microclimatic data recorded was air temperature (Ta, in °C), globe temperature (Tg, in °C), mean radiant temperature (Tmrt, in °C), relative humidity (RH, in %), wind speed (WS, in m/s), wind direction (WD, in °). The instruments were assembled on tripods, 1.1 m high, 30 min before the start of the survey. All instruments were previously calibrated and tested [1]. The equation established by ISO 7726 [4] for forced convection was used to calculate the Tmrt. A software developed at the University of Freiburg, version Holst [9] was used in the calculation of the PET index (in °C). These data is presented in data file: Microclimatic Data. Further information on the climatic data measured by The Fifth Meteorology District of National Meteorology Institute, Brazil (5° DISME/INMET) for Belo Horizonte in these specific days, such as cloudiness values, air temperature, relative humidity and so on, is presented in [1].

### 2.3 Questionnaire survey

Individual and behavioral variables that directly or indirectly influence the thermal perception and/or preference were collected during field surveys (Table 1).

In addition to these individual and behavioral variables and the microclimatic variables measured, subjective responses concerning perception of thermal sensation, thermal comfort evaluation and preferences of thermal sensation were also collected (Table 2).

The administration of questionnaires occurred simultaneously with the microclimatic data measurements during field investigations. The sample frame considered the adult population (20–59 years-old) residing in the city for more than one year and that was in outdoors environments for more than 30 min. A total of 1693 questionnaires were administrated during the field surveys, 835 during the summer (359 in Liberdade Square and 476 in Sete de Setembro Square) and 858 questionnaires during the winter survey (389 in Liberdade Square and 469 in Sete de Setembro Square).

These data is presented in data file: Subjective_Individual_Behavioral Data.

### 2.4 Codes for data interpretation (Data file: Subjective_Individual_Behavioral Data)

We used the following codes (Table 3) to fill in the spreadsheet with the responses to the questionnaire. To all cells of this spreadsheet without answer, we used the number 99.

## Figures and Tables

**Table 1:** Individual and behavioral’ variables considered.

**Individual variables**	**Behavioral variables**
Height	Thermal insulation of clothing
Weigh	Physical activity
Age	Location of them while answering the questionnaires: if in the sun or in shade
Gender	

**Table 2:** Subjective responses considered.

	**Perception of thermal sensation**	**Evaluation of thermal comfort**	**Preference of thermal sensation**
**Question**	“How are you feeling at this precise moment?”	“Do you find this…”	“Please state how you would prefer to be now”
**Scale**	very hot, hot, warm, neutral (neither hot nor cold), cool, cold and very cold	comfortable, slightly uncomfortable, uncomfortable, very uncomfortable	much warmer, warmer, slightly warmer, no change – neither warmer nor cooler, slightly cooler, cooler, much cooler

**Table 3:** Codes for data interpretation (Data file: Subjective_Individual_Behavioral Data).

**Subjective data**	**Individual data**	**Behavioral data**
Perception of thermal sensation:	Height (m):	Thermal insulation of clothing (clo):
1- very cold	1- <1.50	1- 0.3
2- cold	2- 1.5–1.59	2- 0.5
3- cool	3- 1.6–1.69	3- 0.7
4- neutral (neither hot nor cold)	4- 1.7-1.79	4- 1
5- warm	5- >1.80	5- 1.5
6- hot	(P.S: in the summer field investigation was considered 4 to height >1.7 m)	
7- very hot		
Evaluation of thermal comfort:	Weight (kg):	Physical Activity:
1- comfortable	1- <50	1- Seated
2- slightly uncomfortable	2- 50–59	2- Standing
3- uncomfortable	3- 60–69	3- Walking
4- very uncomfortable	4- 70–79	4- Running
	5- >80	5- Riding a bike
Preference of Thermal Sensation:	Age (years-old):	Location of the respondent while answering the questionnaire:
1- much cooler	1- 20–29	1- sun
2- cooler	2- 30–39	2- shade
3- slightly cooler	3- 40–49	
4- no change – neither warmer nor cooler	4- 50–59	
5- slightly warmer	Sex:	
6- warmer	1- Male	
7- much warmer	2- Female	
