# Supplementary material for: Dataset on daytime outdoor thermal comfort for Belo Horizonte, Brazil
Source: Data Brief. 2016 Sep 20;9:530–5. doi: 10.1016/j.dib.2016.09.019 (PMC5054238; doi:10.1016/j.dib.2016.09.019)
Supplement: Supplementary file 1 — Supplementary material [file mmc1.pdf]

AUTHOR DECLARATION

We wish to confirm that there are no known conflicts of interest associated with this publication.

We confirm that the manuscript has been read and approved by all named authors and that there are no other persons who satisfied the criteria for authorship but are not listed. We further confirm that the order of authors listed in the manuscript has been approved by all of us.

We confirm that we have given due consideration to the protection of intellectual property associated with this work and that there are no impediments to publication.

We understand that the Corresponding Author is the sole contact for the Editorial process (including Editorial Manager and direct communications with the office). She is responsible for communicating with the other authors about progress, submissions of revisions and final approval of proofs. We confirm that we have provided a current, correct email address which is accessible by the Corresponding Author.

7th September 2016

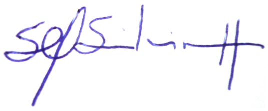

Simone Queiroz da Silveira Hirashima
(Signed by the corresponding author on behalf of all authors)
