# Supplementary material for: Dataset on daytime outdoor thermal comfort for Belo Horizonte, Brazil
Source: Data Brief. 2016 Sep 20;9:530–5. doi: 10.1016/j.dib.2016.09.019 (PMC5054238; doi:10.1016/j.dib.2016.09.019)
Supplement: Supplementary file 2 — Supplementary material [file mmc2.docx]

| **Microclimatic Data** | | | | | | | | | | | | | | | | |
| --- | --- | --- | --- | --- | --- | --- | --- | --- | --- | --- | --- | --- | --- | --- | --- | --- |
| **Liberdade Square – 11th March 2013** | | | | | | | | | | | | | | | | |
|  | **P1** | **P2** | **P1** | **P2** | **P1** | **P2** | **P1** | | **P2** | | **P1** | **P2** | **P1** | **P2** | **P1** | **P2** |
| **Time** | **Ta**  **(°C)** | **Ta**  **(°C)** | **RH**  **(%)** | **RH**  **(%)** | **Tg**  **(°C)** | **Tg**  **(°C)** | **WS**  **(m/s)** | | **WS**  **(m/s)** | | **WD**  **(°)** | **WD**  **(°)** | **Tmrt**  **(°C)** | **Tmrt**  **(°C)** | **PET**  **(°C)** | **PET**  **(°C)** |
| 7:00 | 24,5 | 25,6 | 69,2 | 65,0 | 25,2 | 26,2 | 0,2 | | 0,0 | | 180 | 90 | 26,4 | 26,2 | 25,4 | 27,2 |
| 7:05 | 24,6 | 25,1 | 68,1 | 65,0 | 25,5 | 25,8 | 0,3 | | 0,7 | | 180 | 90 | 27,2 | 27,9 | 25,4 | 24,9 |
| 7:10 | 24,7 | 24,8 | 65,9 | 66,5 | 25,5 | 25,4 | 0,6 | | 0,2 | | 90 | 90 | 28,1 | 26,3 | 25,0 | 25,5 |
| 7:15 | 24,8 | 24,5 | 65,8 | 67,3 | 25,9 | 25,2 | 0,3 | | 0,2 | | 45 | 90 | 27,9 | 26,2 | 25,9 | 25,3 |
| 7:20 | 24,9 | 24,4 | 65,2 | 67,8 | 25,7 | 25,1 | 0,2 | | 0,1 | | 135 | 90 | 26,9 | 25,6 | 25,8 | 25,4 |
| 7:25 | 24,9 | 24,4 | 65,6 | 68,0 | 25,7 | 25,0 | 0,3 | | 0,0 | | 270 | 90 | 27,4 | 25,4 | 25,7 | 26,3 |
| 7:30 | 24,7 | 24,3 | 70,3 | 72,1 | 24,9 | 24,9 | 0,8 | | 0,9 | | 0 | 90 | 25,4 | 27,4 | 23,4 | 23,7 |
| 7:35 | 24,5 | 24,0 | 72,0 | 73,8 | 25,0 | 24,6 | 1,5 | | 0,4 | | 45 | 90 | 27,8 | 26,2 | 23,1 | 24,3 |
| 7:40 | 24,3 | 23,8 | 72,6 | 74,8 | 25,0 | 24,4 | 0,2 | | 0,8 | | 0 | 45 | 26,2 | 26,8 | 25,2 | 23,3 |
| 7:45 | 24,4 | 23,8 | 72,7 | 74,4 | 25,4 | 24,5 | 0,4 | | 0,1 | | 270 | 45 | 27,4 | 25,0 | 25,1 | 24,9 |
| 7:50 | 24,3 | 23,8 | 73,7 | 76,1 | 24,9 | 24,5 | 0,2 | | 0,7 | | 315 | 90 | 25,8 | 26,6 | 25,0 | 23,5 |
| 7:55 | 24,2 | 23,7 | 74,2 | 76,4 | 25,2 | 24,3 | 0,3 | | 0,5 | | 0 | 90 | 27,3 | 26,1 | 25,3 | 23,7 |
| 8:00 | 24,2 | 23,7 | 73,7 | 75,8 | 25,3 | 24,4 | 0,9 | | 0,4 | | 0 | 90 | 29,4 | 26,0 | 24,5 | 24,0 |
| 8:05 | 24,4 | 23,9 | 73,4 | 75,3 | 25,4 | 24,5 | 0,7 | | 0,5 | | 0 | 315 | 28,8 | 26,3 | 24,9 | 24,0 |
| 8:10 | 24,4 | 24,0 | 72,7 | 75,0 | 25,4 | 24,7 | 1,7 | | 0,4 | | 45 | 315 | 31,0 | 26,3 | 23,8 | 24,3 |
| 8:15 | 24,2 | 24,1 | 73,2 | 75,2 | 25,0 | 24,7 | 1,0 | | 0,3 | | 45 | 45 | 28,5 | 26,1 | 23,9 | 24,7 |
| 8:20 | 24,3 | 24,0 | 72,9 | 74,7 | 25,6 | 24,6 | 0,5 | | 1,0 | | 90 | 90 | 29,1 | 27,3 | 25,5 | 23,3 |
| 8:25 | 24,6 | 24,1 | 72,3 | 73,8 | 26,1 | 24,8 | 0,9 | | 0,8 | | 0 | 90 | 31,7 | 27,1 | 25,8 | 23,7 |
| 8:30 | 24,8 | 24,4 | 70,4 | 73,7 | 28,1 | 25,1 | 0,5 | | 0,8 | | 45 | 90 | 36,6 | 27,4 | 29,3 | 24,0 |
| 8:35 | 26,0 | 24,7 | 68,4 | 72,1 | 33,1 | 25,4 | 0,3 | | 0,8 | | 45 | 45 | 46,5 | 27,8 | 36,2 | 24,5 |
| 8:40 | 27,2 | 25,1 | 64,3 | 72,1 | 31,6 | 25,8 | 0,2 | | 0,5 | | 225 | 90 | 37,7 | 27,5 | 32,7 | 25,4 |
| 8:45 | 27,8 | 25,4 | 62,8 | 70,9 | 31,9 | 26,1 | 1,2 | | 0,1 | | 0 | 180 | 48,0 | 26,6 | 34,8 | 26,5 |
| 8:50 | 28,6 | 25,8 | 61,4 | 64,8 | 33,5 | 26,5 | 0,9 | | 0,8 | | 315 | 270 | 50,0 | 28,7 | 37,2 | 25,6 |
| 8:55 | 28,8 | 26,0 | 56,0 | 60,6 | 30,6 | 26,7 | 1,4 | | 1,4 | | 45 | 45 | 39,1 | 29,8 | 31,4 | 25,2 |
| 9:00 | 28,2 | 26,2 | 55,3 | 59,7 | 29,8 | 26,9 | 0,5 | | 1,0 | | 135 | 270 | 33,8 | 29,4 | 30,2 | 25,8 |
| 9:05 | 27,9 | 26,4 | 54,2 | 56,5 | 29,3 | 27,1 | 1,2 | | 0,8 | | 315 | 270 | 35,4 | 29,4 | 29,4 | 26,3 |
| 9:10 | 27,4 | 26,6 | 56,5 | 58,2 | 29,3 | 27,2 | 1,7 | | 0,4 | | 0 | 270 | 38,9 | 28,8 | 29,6 | 27,1 |
| 9:15 | 27,6 | 26,7 | 55,0 | 56,3 | 30,2 | 27,3 | 1,6 | | 0,3 | | 315 | 270 | 43,3 | 28,6 | 31,7 | 27,3 |
| 9:20 | 27,7 | 26,8 | 54,6 | 56,2 | 29,2 | 27,5 | 1,0 | | 0,7 | | 0 | 315 | 35,2 | 29,6 | 29,4 | 26,9 |
| 9:25 | 27,9 | 27,0 | 54,9 | 55,3 | 29,5 | 27,6 | 1,1 | | 0,6 | | 0 | 45 | 36,1 | 29,4 | 29,8 | 27,1 |
| 9:30 | 27,9 | 27,0 | 55,6 | 56,0 | 29,7 | 27,7 | 0,7 | | 0,5 | | 180 | 270 | 35,0 | 29,5 | 30,1 | 27,4 |
| 9:35 | 27,7 | 27,1 | 55,3 | 56,2 | 29,2 | 27,7 | 1,4 | | 0,9 | | 180 | 270 | 36,3 | 30,2 | 29,3 | 27,1 |
| 9:40 | 28,2 | 27,1 | 57,1 | 58,3 | 31,0 | 27,8 | 0,3 | | 1,1 | | 180 | 270 | 36,2 | 30,5 | 32,0 | 26,9 |
| 9:45 | 28,6 | 27,2 | 54,2 | 57,1 | 31,7 | 27,8 | 0,6 | | 0,9 | | 270 | 180 | 40,2 | 30,3 | 33,3 | 27,2 |
| 9:50 | 29,5 | 27,4 | 52,6 | 55,6 | 34,2 | 28,0 | 2,3 | | 1,1 | | 0 | 45 | 60,3 | 30,7 | 39,3 | 27,2 |
| 9:55 | 29,5 | 27,6 | 54,8 | 56,4 | 33,5 | 28,2 | 0,4 | | 0,4 | | 0 | 270 | 41,8 | 29,8 | 35,3 | 28,2 |
| 10:00 | 30,0 | 27,7 | 52,6 | 55,8 | 32,7 | 28,3 | 1,4 | | 1,8 | | 90 | 225 | 44,9 | 32,0 | 34,9 | 27,2 |
| 10:05 | 29,9 | 27,6 | 52,3 | 56,5 | 32,3 | 28,3 | 0,5 | | 1,3 | | 180 | 270 | 38,3 | 31,3 | 33,5 | 27,4 |
| 10:10 | 29,8 | 27,9 | 54,1 | 54,8 | 33,3 | 28,6 | 0,3 | | 1,3 | | 45 | 270 | 39,8 | 31,5 | 34,8 | 27,7 |
| 10:15 | 29,9 | 28,0 | 50,4 | 54,3 | 31,3 | 28,6 | 0,4 | | 0,4 | | 0 | 180 | 34,3 | 30,1 | 31,8 | 28,6 |
| 10:20 | 29,5 | 28,0 | 51,6 | 54,6 | 31,1 | 28,7 | 0,5 | | 0,6 | | 0 | 225 | 35,0 | 30,5 | 31,7 | 28,4 |
| 10:25 | 29,4 | 28,2 | 57,3 | 54,4 | 31,5 | 28,8 | 0,3 | | 0,5 | | 180 | 90 | 35,6 | 30,5 | 32,5 | 28,7 |
| 10:30 | 29,5 | 28,3 | 54,6 | 54,2 | 31,4 | 28,9 | 1,1 | | 0,8 | | 180 | 180 | 39,5 | 31,2 | 32,7 | 28,6 |
| 10:35 | 29,9 | 28,3 | 51,6 | 54,8 | 32,3 | 29,0 | 1,1 | | 0,3 | | 180 | 270 | 41,5 | 30,3 | 33,8 | 29,1 |
| 10:40 | 30,2 | 28,4 | 50,0 | 54,1 | 32,7 | 29,1 | 0,4 | | 1,6 | | 225 | 180 | 38,3 | 32,5 | 34,0 | 28,2 |
| 10:45 | 30,2 | 28,3 | 51,6 | 54,4 | 32,3 | 28,9 | 0,4 | | 1,1 | | 0 | 45 | 36,8 | 31,7 | 33,2 | 28,4 |
| 10:50 | 29,8 | 28,2 | 52,4 | 54,6 | 31,3 | 28,8 | 0,4 | | 0,4 | | 0 | 0 | 34,7 | 30,4 | 32,0 | 28,9 |
| 10:55 | 29,6 | 28,4 | 52,6 | 54,5 | 31,2 | 29,1 | 0,3 | | 0,6 | | 225 | 270 | 34,3 | 30,9 | 31,9 | 28,9 |
| 11:00 | 29,4 | 28,5 | 54,2 | 55,0 | 31,6 | 29,2 | 0,4 | | 0,4 | | 180 | 270 | 36,6 | 30,5 | 32,6 | 29,1 |
| 11:05 | 29,8 | 28,2 | 53,3 | 56,9 | 31,3 | 28,9 | 1,3 | | 0,7 | | 0 | 45 | 37,9 | 31,0 | 32,0 | 28,6 |
| 11:10 | 29,6 | 28,3 | 53,9 | 56,9 | 31,4 | 28,9 | 0,4 | | 0,5 | | 315 | 0 | 35,3 | 30,6 | 32,2 | 28,9 |
| 11:15 | 29,9 | 28,4 | 53,9 | 56,6 | 31,9 | 29,1 | 0,2 | | 0,7 | | 0 | 45 | 35,0 | 31,2 | 32,7 | 28,8 |
| 11:20 | 30,3 | 28,5 | 53,9 | 56,3 | 32,3 | 29,1 | 1,2 | | 1,0 | | 0 | 0 | 40,6 | 31,8 | 33,7 | 28,8 |
| 11:25 | 30,2 | 28,6 | 54,4 | 54,8 | 32,3 | 29,3 | 1,1 | | 0,8 | | 0 | 270 | 40,8 | 31,4 | 33,8 | 28,9 |
| 11:30 | 30,0 | 28,6 | 52,8 | 54,1 | 32,0 | 29,3 | 1,0 | | 0,4 | | 180 | 180 | 39,3 | 30,6 | 33,1 | 29,2 |
| 11:35 | 30,0 | 28,7 | 54,0 | 57,0 | 31,5 | 29,3 | 0,2 | | 0,4 | | 180 | 180 | 33,7 | 30,8 | 32,1 | 29,5 |
| 11:40 | 30,2 | 28,8 | 52,1 | 55,1 | 32,5 | 29,5 | 1,3 | | 0,3 | | 315 | 180 | 42,7 | 30,8 | 34,3 | 29,7 |
| 11:45 | 29,8 | 28,8 | 55,2 | 54,5 | 32,4 | 29,4 | 0,6 | | 1,1 | | 315 | 225 | 39,6 | 32,1 | 33,9 | 29,0 |
| 11:50 | 30,3 | 29,0 | 52,2 | 53,5 | 33,4 | 29,6 | 0,7 | | 0,4 | | 315 | 180 | 42,6 | 31,1 | 35,4 | 29,7 |
| 11:55 | 30,6 | 29,1 | 52,2 | 54,2 | 32,6 | 29,7 | 0,5 | | 0,7 | | 225 | 270 | 37,3 | 31,8 | 33,6 | 29,7 |
| 12:00 | 30,1 | 29,2 | 53,3 | 55,7 | 32,5 | 29,9 | 1,6 | | 0,8 | | 315 | 0 | 43,6 | 32,0 | 34,2 | 29,7 |
| 12:05 | 30,7 | 29,3 | 52,0 | 53,1 | 33,8 | 29,9 | 0,2 | | 0,7 | | 270 | 135 | 37,9 | 32,0 | 34,6 | 29,9 |
| 12:10 | 31,1 | 29,0 | 49,5 | 52,9 | 36,3 | 29,6 | 0,5 | | 0,5 | | 270 | 135 | 48,4 | 31,4 | 39,3 | 29,7 |
| 12:15 | 31,0 | 29,0 | 50,6 | 54,1 | 32,1 | 29,6 | 1,4 | | 0,8 | | 225 | 135 | 37,5 | 31,7 | 32,8 | 29,4 |
| 12:20 | 30,8 | 28,9 | 50,7 | 54,6 | 33,1 | 29,5 | 3,9 | | 0,9 | | 225 | 135 | 51,0 | 31,8 | 35,4 | 29,2 |
| 12:25 | 30,9 | 28,8 | 50,3 | 54,4 | 33,4 | 29,5 | 2,8 | | 0,9 | | 225 | 135 | 49,9 | 31,8 | 36,0 | 29,1 |
| 12:30 | 30,6 | 28,8 | 54,7 | 55,9 | 33,2 | 29,5 | 3,5 | | 0,9 | | 315 | 315 | 51,9 | 31,8 | 35,8 | 29,1 |
| 12:35 | 30,3 | 28,8 | 52,1 | 54,9 | 33,3 | 29,5 | 1,3 | | 0,9 | | 225 | 225 | 45,7 | 31,8 | 35,6 | 29,1 |
| 12:40 | 30,7 | 28,8 | 51,8 | 53,6 | 32,7 | 29,5 | 0,8 | | 0,9 | | 225 | 135 | 39,2 | 31,9 | 34,0 | 29,1 |
| 12:45 | 30,7 | 28,9 | 50,6 | 53,9 | 32,9 | 29,6 | 4,6 | | 1,0 | | 315 | 315 | 52,0 | 32,0 | 35,0 | 29,2 |
| 12:50 | 30,1 | 28,8 | 52,7 | 55,2 | 33,2 | 29,5 | 4,5 | | 0,9 | | 225 | 225 | 58,8 | 31,9 | 36,7 | 29,2 |
| 12:55 | 30,8 | 28,9 | 52,2 | 56,1 | 34,5 | 29,5 | 1,7 | | 1,0 | | 315 | 225 | 52,2 | 32,0 | 38,2 | 29,2 |
| 13:00 | 31,6 | 29,0 | 48,7 | 54,8 | 34,7 | 29,6 | 3,4 | | 0,9 | | 315 | 315 | 56,3 | 31,9 | 38,6 | 29,3 |
| 13:05 | 31,8 | 29,1 | 50,6 | 55,3 | 35,3 | 29,7 | 1,2 | | 0,7 | | 180 | 315 | 49,2 | 31,8 | 38,7 | 29,7 |
| 13:10 | 32,2 | 29,4 | 50,1 | 55,4 | 35,6 | 30,1 | 1,4 | | 0,9 | | 315 | 315 | 50,0 | 32,3 | 39,0 | 29,8 |
| 13:15 | 32,6 | 29,6 | 50,3 | 54,9 | 35,3 | 30,2 | 4,7 | | 0,9 | | 315 | 315 | 58,3 | 32,6 | 39,4 | 30,1 |
| 13:20 | 32,3 | 29,6 | 49,8 | 54,5 | 35,2 | 30,3 | 1,4 | | 0,8 | | 225 | 225 | 47,5 | 32,5 | 38,0 | 30,2 |
| 13:25 | 32,6 | 30,0 | 50,2 | 51,8 | 35,7 | 30,6 | 2,6 | | 0,7 | | 225 | 315 | 54,9 | 32,6 | 40,0 | 30,7 |
| 13:30 | 32,0 | 30,0 | 47,5 | 51,2 | 34,8 | 30,7 | 1,1 | | 0,9 | | 225 | 225 | 45,6 | 33,0 | 37,3 | 30,6 |
| 13:35 | 32,3 | 29,8 | 47,4 | 51,2 | 36,0 | 30,5 | 3,8 | | 0,7 | | 315 | 45 | 62,8 | 32,5 | 41,4 | 30,4 |
| 13:40 | 32,3 | 29,6 | 48,1 | 52,8 | 36,2 | 30,2 | 1,4 | | 0,8 | | 315 | 225 | 52,6 | 32,5 | 40,2 | 30,2 |
| 13:45 | 32,2 | 29,9 | 48,1 | 51,9 | 34,6 | 30,5 | 3,4 | | 0,8 | | 315 | 315 | 52,6 | 32,7 | 37,9 | 30,5 |
| 13:50 | 31,9 | 30,2 | 47,8 | 50,0 | 34,6 | 30,8 | 0,6 | | 0,8 | | 315 | 315 | 41,7 | 32,9 | 36,3 | 30,8 |
| 13:55 | 31,5 | 30,1 | 50,3 | 51,5 | 33,5 | 30,8 | 0,5 | | 0,9 | | 225 | 315 | 38,8 | 33,2 | 34,9 | 30,7 |
| 14:00 | 32,2 | 30,1 | 48,0 | 50,7 | 34,9 | 30,8 | 0,5 | | 0,9 | | 0 | 315 | 41,6 | 33,1 | 36,7 | 30,7 |
| 14:05 | 31,7 | 29,9 | 48,9 | 51,5 | 34,8 | 30,6 | 2,5 | | 1,2 | | 225 | 180 | 53,4 | 33,3 | 38,5 | 30,3 |
| 14:10 | 32,0 | 30,2 | 47,8 | 51,3 | 35,7 | 30,8 | 1,8 | | 0,5 | | 315 | 270 | 53,8 | 32,5 | 39,8 | 31,0 |
| 14:15 | 31,5 | 30,0 | 49,3 | 52,4 | 34,5 | 30,7 | 3,2 | | 0,3 | | 225 | 180 | 54,9 | 31,9 | 38,1 | 30,9 |
| 14:20 | 31,5 | 29,9 | 49,5 | 50,4 | 33,8 | 30,5 | 1,7 | | 0,7 | | 315 | 315 | 45,4 | 32,6 | 36,1 | 30,6 |
| 14:25 | 31,8 | 29,8 | 47,8 | 51,2 | 36,0 | 30,4 | 2,6 | | 0,8 | | 315 | 315 | 60,8 | 32,6 | 41,4 | 30,3 |
| 14:30 | 32,1 | 30,1 | 47,7 | 53,1 | 35,7 | 30,8 | 4,5 | | 0,2 | | 225 | 315 | 64,8 | 31,6 | 41,2 | 31,1 |
| 14:35 | 32,0 | 30,2 | 46,8 | 51,3 | 34,1 | 30,8 | 2,9 | | 0,4 | | 225 | 225 | 48,3 | 32,3 | 36,5 | 31,1 |
| 14:40 | 32,2 | 30,0 | 46,6 | 50,7 | 34,5 | 30,6 | 4,4 | | 1,2 | | 225 | 225 | 53,8 | 33,4 | 37,6 | 30,4 |
| 14:45 | 31,7 | 29,9 | 47,9 | 50,4 | 34,5 | 30,6 | 1,1 | | 0,7 | | 315 | 315 | 44,7 | 32,6 | 36,7 | 30,6 |
| 14:50 | 31,9 | 30,0 | 46,9 | 49,9 | 34,7 | 30,6 | 2,8 | | 0,8 | | 180 | 225 | 53,1 | 32,8 | 38,3 | 30,6 |
| 14:55 | 32,2 | 30,1 | 47,5 | 49,9 | 35,7 | 30,8 | 1,3 | | 0,5 | | 315 | 315 | 49,9 | 32,4 | 39,1 | 30,9 |
| 15:00 | 32,2 | 30,4 | 47,8 | 51,9 | 34,3 | 31,0 | 0,4 | | 0,3 | | 225 | 315 | 39,1 | 32,2 | 35,6 | 31,3 |
| 15:05 | 32,1 | 30,6 | 48,9 | 51,1 | 35,1 | 31,3 | 0,4 | | 0,2 | | 225 | 225 | 40,8 | 32,3 | 36,4 | 31,7 |
| 15:10 | 32,0 | 30,6 | 47,7 | 49,7 | 32,6 | 31,2 | 0,3 | | 0,4 | | 225 | 315 | 33,8 | 32,7 | 33,1 | 31,5 |
| 15:15 | 31,7 | 30,5 | 49,1 | 50,2 | 33,9 | 31,1 | 0,6 | | 0,5 | | 225 | 315 | 40,1 | 32,8 | 35,4 | 31,4 |
| 15:20 | 32,6 | 30,7 | 46,5 | 49,5 | 36,6 | 31,4 | 1,4 | | 0,1 | | 225 | 225 | 53,6 | 32,1 | 40,9 | 31,8 |
| 15:25 | 33,4 | 31,0 | 45,8 | 50,1 | 35,6 | 31,6 | 0,8 | | 0,3 | | 315 | 225 | 42,4 | 32,8 | 37,4 | 32,0 |
| 15:30 | 33,1 | 30,8 | 46,9 | 50,9 | 32,7 | 31,4 | 3,5 | | 2,2 | | 315 | 225 | 30,0 | 35,4 | 31,7 | 31,2 |
| 15:35 | 33,0 | 30,5 | 46,8 | 51,6 | 35,3 | 31,1 | 1,5 | | 0,7 | | 135 | 225 | 45,8 | 33,1 | 37,8 | 31,3 |
| 15:40 | 33,2 | 30,6 | 46,2 | 51,0 | 36,6 | 31,2 | 1,9 | | 0,3 | | 225 | 225 | 53,7 | 32,4 | 40,8 | 31,5 |
| 15:45 | 33,4 | 30,7 | 48,2 | 52,0 | 34,8 | 31,3 | 1,5 | | 0,3 | | 225 | 225 | 41,4 | 32,5 | 36,4 | 31,7 |
| 15:50 | 33,6 | 30,8 | 47,4 | 51,2 | 36,3 | 31,5 | 1,3 | | 0,3 | | 225 | 315 | 47,5 | 32,6 | 39,3 | 31,8 |
| 15:55 | 33,6 | 30,9 | 46,1 | 54,1 | 35,3 | 31,5 | 0,9 | | 0,3 | | 225 | 315 | 41,2 | 32,6 | 37,0 | 31,9 |
| 16:00 | 33,3 | 30,7 | 46,7 | 51,0 | 36,0 | 31,3 | 0,9 | | 2,5 | | 225 | 315 | 45,3 | 35,6 | 38,6 | 31,0 |
| 16:05 | 32,9 | 30,6 | 47,9 | 52,6 | 34,3 | 31,2 | 1,9 | | 1,2 | | 180 | 315 | 41,4 | 34,0 | 35,7 | 31,2 |
| 16:10 | 32,5 | 30,6 | 48,4 | 52,8 | 32,9 | 31,2 | 1,6 | | 0,4 | | 180 | 45 | 34,7 | 32,8 | 32,9 | 31,6 |
| 16:15 | 32,6 | 30,3 | 48,0 | 52,4 | 33,5 | 30,9 | 1,1 | | 0,4 | | 135 | 45 | 36,9 | 32,3 | 34,2 | 31,2 |
| 16:20 | 33,1 | 30,3 | 48,4 | 54,0 | 35,0 | 30,9 | 0,6 | | 0,6 | | 225 | 45 | 40,5 | 32,7 | 36,6 | 31,1 |
| 16:25 | 32,5 | 30,3 | 47,9 | 54,0 | 31,6 | 31,0 | 0,5 | | 0,2 | | 225 | 45 | 29,3 | 31,9 | 31,2 | 31,4 |
| 16:30 | 32,0 | 30,4 | 50,1 | 52,6 | 31,7 | 31,0 | 0,5 | | 0,7 | | 315 | 45 | 30,8 | 33,0 | 31,5 | 31,2 |
| 16:35 | 31,6 | 30,4 | 50,4 | 53,2 | 31,4 | 31,1 | 0,2 | | 1,2 | | 315 | 45 | 31,1 | 33,9 | 31,7 | 31,0 |
| 16:40 | 31,4 | 30,5 | 53,0 | 53,3 | 31,5 | 31,2 | 1,1 | | 0,4 | | 315 | 315 | 32,2 | 32,6 | 31,3 | 31,5 |
| 16:45 | 30,9 | 30,6 | 50,5 | 51,4 | 30,7 | 31,2 | 2,0 | | 0,6 | | 315 | 315 | 29,6 | 33,1 | 29,4 | 31,5 |
| 16:50 | 30,8 | 30,5 | 50,6 | 51,1 | 31,0 | 31,2 | 2,8 | | 0,5 | | 180 | 315 | 32,9 | 32,9 | 30,1 | 31,4 |
| 16:55 | 30,2 | 30,2 | 51,6 | 53,3 | 30,1 | 30,9 | 1,2 | | 0,3 | | 180 | 315 | 29,5 | 32,1 | 29,1 | 31,2 |
| 17:00 | 30,2 | 30,1 | 52,2 | 52,5 | 30,3 | 30,7 | 2,3 | | 0,6 | | 315 | 315 | 31,3 | 32,6 | 29,2 | 30,9 |
| **Sete de Setembro Square – 13th March 2013** | | | | | | | | | | | | | | | | |
|  | **P3** | **P4** | **P3** | **P4** | **P3** | **P4** | **P3** | | **P4** | | **P3** | **P4** | **P3** | **P4** | **P3** | **P4** |
| **Time** | **Ta**  **(°C)** | **Ta**  **(°C)** | **RH**  **(%)** | **RH**  **(%)** | **Tg**  **(°C)** | **Tg**  **(°C)** | **WS**  **(m/s)** | | **WS**  **(m/s)** | | **WD**  **(°)** | **WD**  **(°)** | **Tmrt**  **(°C)** | **Tmrt**  **(°C)** | **PET**  **(°C)** | **PET**  **(°C)** |
| 7:00 | 25,2 | 25,2 | 67,2 | 66,9 | 26,1 | 25,4 | 0,1 | 0,0 | | 90 | | 135 | 26,9 | 25,4 | 26,5 | 26,6 |
| 7:05 | 25,4 | 25,3 | 66,2 | 66,8 | 25,9 | 25,9 | 1,7 | 0,1 | | 0 | | 90 | 29,0 | 26,7 | 24,0 | 26,5 |
| 7:10 | 25,5 | 25,4 | 65,9 | 66,1 | 26,3 | 25,9 | 1,6 | 0,5 | | 0 | | 135 | 30,5 | 27,3 | 24,8 | 25,4 |
| 7:15 | 25,6 | 25,5 | 64,8 | 65,6 | 26,4 | 25,9 | 0,6 | 0,1 | | 0 | | 225 | 28,8 | 26,4 | 25,9 | 26,4 |
| 7:20 | 25,7 | 25,6 | 64,9 | 65,0 | 26,3 | 26,1 | 0,7 | 0,3 | | 270 | | 0 | 28,3 | 27,3 | 25,6 | 26,1 |
| 7:25 | 26,0 | 25,7 | 64,0 | 64,6 | 26,8 | 26,1 | 1,5 | 0,1 | | 0 | | 270 | 30,8 | 26,6 | 25,5 | 26,6 |
| 7:30 | 26,3 | 25,8 | 62,0 | 63,4 | 27,2 | 26,3 | 2,7 | 0,3 | | 0 | | 315 | 33,6 | 27,1 | 25,5 | 26,1 |
| 7:35 | 26,4 | 25,9 | 62,0 | 63,9 | 27,1 | 26,5 | 1,7 | 0,2 | | 0 | | 315 | 30,8 | 27,4 | 25,6 | 26,7 |
| 7:40 | 26,6 | 25,9 | 62,4 | 63,8 | 27,8 | 26,3 | 0,8 | 0,1 | | 0 | | 315 | 32,1 | 26,7 | 27,7 | 26,7 |
| 7:45 | 26,9 | 26,1 | 61,5 | 63,5 | 28,0 | 27,0 | 0,1 | 0,0 | | 90 | | 0 | 29,3 | 27,5 | 28,6 | 28,1 |
| 7:50 | 27,1 | 26,3 | 60,5 | 63,1 | 28,0 | 27,2 | 1,5 | 0,1 | | 0 | | 315 | 32,5 | 27,8 | 27,2 | 27,5 |
| 7:55 | 27,3 | 26,4 | 59,9 | 62,1 | 28,6 | 26,9 | 0,3 | 0,1 | | 0 | | 90 | 31,1 | 27,4 | 29,0 | 27,3 |
| 8:00 | 27,5 | 26,5 | 58,7 | 62,4 | 30,2 | 27,4 | 2,6 | 0,1 | | 0 | | 315 | 48,1 | 28,5 | 31,9 | 28,0 |
| 8:05 | 27,6 | 26,7 | 58,5 | 60,9 | 30,8 | 27,4 | 1,7 | 0,1 | | 0 | | 0 | 47,1 | 28,0 | 33,1 | 27,8 |
| 8:10 | 28,2 | 26,8 | 57,7 | 60,9 | 33,0 | 27,4 | 0,2 | 0,1 | | 90 | | 90 | 39,2 | 28,1 | 34,0 | 27,9 |
| 8:15 | 28,4 | 26,9 | 56,7 | 60,3 | 30,7 | 27,5 | 1,1 | 0,1 | | 225 | | 90 | 40,3 | 28,0 | 32,1 | 27,9 |
| 8:20 | 28,6 | 27,0 | 54,2 | 59,2 | 32,1 | 27,6 | 2,3 | 0,1 | | 0 | | 45 | 52,1 | 28,3 | 35,1 | 28,1 |
| 8:25 | 28,7 | 27,1 | 53,7 | 58,3 | 31,6 | 27,6 | 0,9 | 0,1 | | 0 | | 0 | 41,9 | 28,1 | 33,4 | 28,0 |
| 8:30 | 29,1 | 27,2 | 54,3 | 58,4 | 32,8 | 27,9 | 1,4 | 0,1 | | 0 | | 90 | 48,7 | 28,4 | 35,7 | 28,2 |
| 8:35 | 29,3 | 27,3 | 54,2 | 57,0 | 32,6 | 27,8 | 1,9 | 0,2 | | 225 | | 90 | 49,4 | 28,5 | 35,3 | 27,9 |
| 8:40 | 29,4 | 27,3 | 53,0 | 57,8 | 33,4 | 28,1 | 1,4 | 0,1 | | 0 | | 90 | 50,6 | 29,0 | 36,8 | 28,6 |
| 8:45 | 30,1 | 27,5 | 52,3 | 57,6 | 34,6 | 28,3 | 0,7 | 0,1 | | 180 | | 270 | 47,4 | 29,0 | 37,6 | 28,7 |
| 8:50 | 30,5 | 27,7 | 50,7 | 56,5 | 34,4 | 28,6 | 1,9 | 0,1 | | 0 | | 315 | 54,0 | 29,4 | 38,3 | 29,0 |
| 8:55 | 30,4 | 27,9 | 49,7 | 56,0 | 33,2 | 28,7 | 1,4 | 0,3 | | 0 | | 315 | 45,7 | 30,2 | 35,5 | 28,8 |
| 9:00 | 30,0 | 28,0 | 51,0 | 56,3 | 32,0 | 28,7 | 0,8 | 0,1 | | 0 | | 315 | 38,5 | 29,2 | 33,1 | 29,0 |
| 9:05 | 30,1 | 28,2 | 50,2 | 56,3 | 31,4 | 29,2 | 2,4 | 0,1 | | 0 | | 0 | 39,6 | 30,3 | 31,8 | 29,7 |
| 9:10 | 30,2 | 28,4 | 50,4 | 54,4 | 31,8 | 29,2 | 1,4 | 0,2 | | 0 | | 45 | 39,0 | 30,5 | 32,6 | 29,5 |
| 9:15 | 30,2 | 28,6 | 50,9 | 54,0 | 32,0 | 29,2 | 2,4 | 0,1 | | 0 | | 315 | 43,8 | 29,8 | 33,4 | 29,6 |
| 9:20 | 30,2 | 28,5 | 50,0 | 53,4 | 32,1 | 28,9 | 0,4 | 0,3 | | 0 | | 0 | 36,2 | 29,7 | 32,9 | 28,9 |
| 9:25 | 30,4 | 28,5 | 49,1 | 53,3 | 33,1 | 29,2 | 1,8 | 0,0 | | 45 | | 90 | 46,7 | 29,5 | 35,4 | 30,0 |
| 9:30 | 31,1 | 28,5 | 47,9 | 52,9 | 34,5 | 29,3 | 0,7 | 0,0 | | 135 | | 270 | 44,6 | 29,7 | 36,9 | 30,1 |
| 9:35 | 31,6 | 28,8 | 47,1 | 54,1 | 35,0 | 30,1 | 1,6 | 0,1 | | 0 | | 180 | 50,9 | 31,5 | 38,5 | 30,6 |
| 9:40 | 31,8 | 29,1 | 44,7 | 52,1 | 33,9 | 30,3 | 1,4 | 0,2 | | 0 | | 0 | 43,2 | 32,2 | 35,7 | 30,8 |
| 9:45 | 31,3 | 29,3 | 46,2 | 51,5 | 31,8 | 29,8 | 1,9 | 0,1 | | 0 | | 0 | 34,2 | 30,1 | 31,4 | 30,1 |
| 9:50 | 30,8 | 29,3 | 47,3 | 50,3 | 31,0 | 29,5 | 1,8 | 0,1 | | 0 | | 45 | 32,4 | 29,8 | 30,3 | 29,9 |
| 9:55 | 30,6 | 29,3 | 47,7 | 51,2 | 31,0 | 30,0 | 1,5 | 0,1 | | 0 | | 0 | 33,0 | 30,6 | 30,5 | 30,4 |
| 10:00 | 30,4 | 29,3 | 48,0 | 50,2 | 31,0 | 29,5 | 2,0 | 0,4 | | 315 | | 0 | 34,7 | 29,9 | 30,6 | 29,3 |
| 10:05 | 30,1 | 29,2 | 48,6 | 50,9 | 31,1 | 29,7 | 1,8 | 0,1 | | 0 | | 0 | 36,4 | 30,2 | 31,1 | 30,1 |
| 10:10 | 30,3 | 29,2 | 47,8 | 50,3 | 31,6 | 29,7 | 1,8 | 0,2 | | 270 | | 270 | 38,3 | 30,4 | 32,0 | 29,9 |
| 10:15 | 30,6 | 29,3 | 47,4 | 49,9 | 31,3 | 30,0 | 2,0 | 0,3 | | 0 | | 315 | 35,4 | 31,2 | 31,1 | 30,1 |
| 10:20 | 30,6 | 29,2 | 47,4 | 50,4 | 31,7 | 29,6 | 3,4 | 0,2 | | 45 | | 315 | 39,6 | 30,2 | 31,6 | 29,8 |
| 10:25 | 30,6 | 29,2 | 47,5 | 50,2 | 31,4 | 29,6 | 2,5 | 0,2 | | 45 | | 315 | 36,6 | 30,2 | 31,2 | 29,8 |
| 10:30 | 30,5 | 29,2 | 45,7 | 49,3 | 30,9 | 29,8 | 3,0 | 0,2 | | 315 | | 315 | 34,3 | 30,7 | 30,0 | 30,0 |
| 10:35 | 30,2 | 29,3 | 46,6 | 49,1 | 31,3 | 29,9 | 2,7 | 0,3 | | 0 | | 0 | 38,5 | 31,0 | 31,2 | 30,0 |
| 10:40 | 30,3 | 29,3 | 46,9 | 49,6 | 32,1 | 30,2 | 1,3 | 0,0 | | 225 | | 90 | 39,5 | 30,6 | 33,0 | 30,9 |
| 10:45 | 30,5 | 29,4 | 46,7 | 48,7 | 31,2 | 29,7 | 1,5 | 0,4 | | 0 | | 270 | 34,9 | 30,2 | 31,1 | 29,5 |
| 10:50 | 30,4 | 29,4 | 46,5 | 48,8 | 31,2 | 29,9 | 0,2 | 0,2 | | 90 | | 315 | 32,6 | 30,6 | 31,7 | 30,1 |
| 10:55 | 30,4 | 29,3 | 47,1 | 49,4 | 31,3 | 29,7 | 1,8 | 0,2 | | 0 | | 270 | 36,5 | 30,2 | 31,4 | 29,8 |
| 11:00 | 30,3 | 29,3 | 47,5 | 49,4 | 31,5 | 29,8 | 0,2 | 0,1 | | 90 | | 315 | 33,0 | 30,3 | 31,8 | 30,2 |
| 11:05 | 30,5 | 29,3 | 47,0 | 49,2 | 31,2 | 29,6 | 2,5 | 0,1 | | 0 | | 315 | 35,8 | 29,8 | 30,8 | 29,9 |
| 11:10 | 30,2 | 29,3 | 47,8 | 50,2 | 30,8 | 30,0 | 1,5 | 0,1 | | 0 | | 270 | 33,8 | 30,6 | 30,5 | 30,3 |
| 11:15 | 30,3 | 29,5 | 47,9 | 49,8 | 31,3 | 30,2 | 2,5 | 0,3 | | 0 | | 270 | 38,1 | 31,5 | 31,4 | 30,4 |
| 11:20 | 30,3 | 29,6 | 48,1 | 49,7 | 31,5 | 30,3 | 2,2 | 0,2 | | 0 | | 315 | 38,9 | 31,4 | 31,9 | 30,6 |
| 11:25 | 30,7 | 29,6 | 47,9 | 50,0 | 32,5 | 30,1 | 1,1 | 0,2 | | 0 | | 315 | 39,6 | 30,9 | 33,6 | 30,4 |
| 11:30 | 30,8 | 29,5 | 47,1 | 50,0 | 31,5 | 30,2 | 1,4 | 0,2 | | 0 | | 315 | 35,1 | 31,4 | 31,6 | 30,6 |
| 11:35 | 30,6 | 29,6 | 47,9 | 50,2 | 32,2 | 30,5 | 1,8 | 0,1 | | 0 | | 270 | 40,2 | 31,5 | 33,0 | 31,0 |
| 11:40 | 30,9 | 29,6 | 47,6 | 50,5 | 33,3 | 30,5 | 2,7 | 0,2 | | 0 | | 315 | 48,9 | 31,8 | 35,7 | 30,8 |
| 11:45 | 31,0 | 29,6 | 47,3 | 50,4 | 32,9 | 30,7 | 0,8 | 0,2 | | 0 | | 315 | 39,2 | 32,1 | 34,1 | 31,0 |
| 11:50 | 31,0 | 29,8 | 46,8 | 49,3 | 32,7 | 30,5 | 2,9 | 0,1 | | 0 | | 315 | 44,4 | 31,4 | 34,0 | 31,0 |
| 11:55 | 31,4 | 29,9 | 46,8 | 49,9 | 33,5 | 31,3 | 0,5 | 0,1 | | 90 | | 270 | 38,5 | 32,5 | 34,6 | 31,7 |
| 12:00 | 31,8 | 30,1 | 45,1 | 49,5 | 33,5 | 32,1 | 1,5 | 0,1 | | 315 | | 225 | 41,6 | 34,3 | 34,9 | 32,7 |
| 12:05 | 31,5 | 30,1 | 46,5 | 49,2 | 34,2 | 31,0 | 1,3 | 0,2 | | 225 | | 270 | 45,6 | 32,6 | 36,6 | 31,5 |
| 12:10 | 32,0 | 30,4 | 43,9 | 46,7 | 33,6 | 31,5 | 4,6 | 0,3 | | 45 | | 270 | 47,4 | 33,4 | 35,1 | 31,9 |
| 12:15 | 32,3 | 30,5 | 44,2 | 47,8 | 34,2 | 31,3 | 0,5 | 0,2 | | 45 | | 315 | 39,1 | 32,3 | 35,5 | 31,6 |
| 12:20 | 32,2 | 30,6 | 43,0 | 46,7 | 33,7 | 31,4 | 2,4 | 0,1 | | 315 | | 315 | 42,7 | 32,4 | 35,0 | 31,9 |
| 12:25 | 32,7 | 30,8 | 43,4 | 46,3 | 36,6 | 31,8 | 0,4 | 0,2 | | 315 | | 270 | 44,4 | 33,5 | 38,6 | 32,3 |
| 12:30 | 33,1 | 31,1 | 43,3 | 46,8 | 34,9 | 31,9 | 0,3 | 0,3 | | 90 | | 315 | 38,4 | 33,3 | 36,0 | 32,2 |
| 12:35 | 32,4 | 30,9 | 44,1 | 47,9 | 32,2 | 31,1 | 1,3 | 0,0 | | 315 | | 0 | 31,4 | 31,2 | 31,7 | 31,8 |
| 12:40 | 32,1 | 30,7 | 44,4 | 47,7 | 33,6 | 31,1 | 1,9 | 0,2 | | 315 | | 270 | 41,8 | 31,5 | 35,0 | 31,3 |
| 12:45 | 32,7 | 31,0 | 43,5 | 47,2 | 35,8 | 32,3 | 1,1 | 0,1 | | 225 | | 315 | 47,6 | 33,7 | 38,8 | 32,8 |
| 12:50 | 32,9 | 31,4 | 41,7 | 44,9 | 35,7 | 32,4 | 0,8 | 0,2 | | 225 | | 135 | 44,9 | 33,8 | 38,1 | 32,8 |
| 12:55 | 33,7 | 31,4 | 41,5 | 45,1 | 37,2 | 31,8 | 0,6 | 0,1 | | 315 | | 180 | 45,9 | 32,1 | 39,5 | 32,1 |
| 13:00 | 33,6 | 31,5 | 41,0 | 45,2 | 35,0 | 32,0 | 1,2 | 0,1 | | 180 | | 0 | 40,6 | 32,4 | 36,4 | 32,3 |
| 13:05 | 33,7 | 31,7 | 39,3 | 43,7 | 35,7 | 32,6 | 0,7 | 0,0 | | 315 | | 225 | 41,8 | 33,1 | 37,4 | 33,1 |
| 13:10 | 33,1 | 32,1 | 40,5 | 42,5 | 34,4 | 32,7 | 0,8 | 0,2 | | 315 | | 270 | 38,3 | 33,6 | 35,3 | 33,1 |
| 13:15 | 33,4 | 32,2 | 40,5 | 42,7 | 36,0 | 32,9 | 5,3 | 0,2 | | 0 | | 315 | 59,4 | 34,0 | 40,3 | 33,3 |
| 13:20 | 33,4 | 32,2 | 40,5 | 42,9 | 35,4 | 34,1 | 0,7 | 0,3 | | 0 | | 270 | 41,3 | 37,3 | 37,0 | 34,8 |
| 13:25 | 33,7 | 32,5 | 42,2 | 43,8 | 36,6 | 34,2 | 0,9 | 0,3 | | 315 | | 315 | 46,5 | 37,1 | 39,4 | 34,9 |
| 13:30 | 33,8 | 32,5 | 41,9 | 44,3 | 34,9 | 33,0 | 0,9 | 0,4 | | 0 | | 315 | 38,5 | 34,1 | 35,9 | 33,4 |
| 13:35 | 34,0 | 32,4 | 41,7 | 44,9 | 36,2 | 33,0 | 0,3 | 0,6 | | 315 | | 270 | 40,1 | 34,7 | 37,3 | 33,4 |
| 13:40 | 33,9 | 32,5 | 42,3 | 44,7 | 36,1 | 32,7 | 0,4 | 1,4 | | 315 | | 0 | 40,6 | 33,8 | 37,4 | 32,6 |
| 13:45 | 33,6 | 32,3 | 42,9 | 45,1 | 33,7 | 32,7 | 0,7 | 1,3 | | 0 | | 270 | 33,9 | 34,4 | 33,9 | 32,8 |
| 13:50 | 33,4 | 32,4 | 42,7 | 45,5 | 36,3 | 33,3 | 1,4 | 0,8 | | 0 | | 270 | 48,6 | 36,3 | 39,4 | 34,0 |
| 13:55 | 33,9 | 33,1 | 42,1 | 44,2 | 36,2 | 36,0 | 0,8 | 0,8 | | 0 | | 0 | 43,8 | 45,2 | 38,4 | 38,5 |
| 14:00 | 33,9 | 32,9 | 42,4 | 44,1 | 35,6 | 34,4 | 0,9 | 1,2 | | 0 | | 270 | 41,4 | 40,2 | 37,2 | 35,6 |
| 14:05 | 33,9 | 32,8 | 42,3 | 44,7 | 36,7 | 36,2 | 0,4 | 0,3 | | 0 | | 135 | 42,3 | 41,9 | 38,3 | 37,6 |
| 14:10 | 34,0 | 33,5 | 42,5 | 44,1 | 36,8 | 37,2 | 0,7 | 0,3 | | 0 | | 315 | 44,7 | 43,1 | 39,0 | 38,6 |
| 14:15 | 34,4 | 32,9 | 41,4 | 44,1 | 36,9 | 32,9 | 2,1 | 0,2 | | 0 | | 270 | 50,0 | 32,8 | 40,2 | 33,1 |
| 14:20 | 34,6 | 32,6 | 40,5 | 44,1 | 37,2 | 32,8 | 0,5 | 0,1 | | 90 | | 90 | 43,3 | 32,9 | 39,0 | 33,1 |
| 14:25 | 34,6 | 32,7 | 40,9 | 44,7 | 35,7 | 32,9 | 0,8 | 0,3 | | 315 | | 315 | 39,0 | 33,3 | 36,8 | 33,2 |
| 14:30 | 34,7 | 32,8 | 41,0 | 44,2 | 36,7 | 33,5 | 0,6 | 0,2 | | 315 | | 270 | 42,1 | 34,5 | 38,4 | 34,0 |
| 14:35 | 34,2 | 32,7 | 41,4 | 44,5 | 35,1 | 33,1 | 2,6 | 0,2 | | 0 | | 270 | 40,9 | 33,5 | 36,4 | 33,4 |
| 14:40 | 34,3 | 32,6 | 41,5 | 44,7 | 37,1 | 32,9 | 2,2 | 0,1 | | 225 | | 0 | 52,8 | 33,1 | 41,1 | 33,2 |
| 14:45 | 35,1 | 32,8 | 39,8 | 43,7 | 37,5 | 33,3 | 1,3 | 0,3 | | 225 | | 315 | 47,3 | 34,1 | 40,4 | 33,6 |
| 14:50 | 35,1 | 32,8 | 40,7 | 44,8 | 38,0 | 33,0 | 2,1 | 0,2 | | 225 | | 90 | 53,6 | 33,1 | 42,4 | 33,2 |
| 14:55 | 35,3 | 32,7 | 40,3 | 44,8 | 36,8 | 32,9 | 0,4 | 0,1 | | 315 | | 270 | 40,1 | 33,1 | 38,0 | 33,3 |
| 15:00 | 34,7 | 32,8 | 41,4 | 44,9 | 35,2 | 33,5 | 0,5 | 0,1 | | 0 | | 315 | 36,5 | 34,0 | 35,9 | 33,8 |
| 15:05 | 34,8 | 33,1 | 41,2 | 46,1 | 37,7 | 34,6 | 0,5 | 0,4 | | 0 | | 315 | 44,8 | 37,6 | 39,9 | 35,5 |
| 15:10 | 34,6 | 33,2 | 40,8 | 43,3 | 36,1 | 33,8 | 0,5 | 0,2 | | 0 | | 270 | 39,6 | 34,6 | 37,3 | 34,2 |
| 15:15 | 34,7 | 33,2 | 41,0 | 43,6 | 37,5 | 33,3 | 0,6 | 0,6 | | 0 | | 135 | 45,1 | 33,5 | 39,8 | 33,4 |
| 15:20 | 34,8 | 33,1 | 41,3 | 44,1 | 36,2 | 33,1 | 0,9 | 0,7 | | 315 | | 270 | 41,3 | 33,1 | 37,9 | 33,1 |
| 15:25 | 34,9 | 33,0 | 41,0 | 44,4 | 36,1 | 33,2 | 0,3 | 0,3 | | 0 | | 315 | 38,3 | 33,5 | 37,0 | 33,5 |
| 15:30 | 34,6 | 32,9 | 41,4 | 44,3 | 33,9 | 32,7 | 1,5 | 0,1 | | 0 | | 315 | 30,3 | 32,6 | 33,2 | 33,1 |
| 15:35 | 34,5 | 32,8 | 41,5 | 44,6 | 34,9 | 32,8 | 0,3 | 0,9 | | 315 | | 315 | 35,8 | 32,8 | 35,5 | 32,7 |
| 15:40 | 33,9 | 32,5 | 42,5 | 45,2 | 34,8 | 32,5 | 2,1 | 0,2 | | 0 | | 270 | 40,0 | 32,4 | 36,0 | 32,7 |
| 15:45 | 34,0 | 32,5 | 42,3 | 44,6 | 35,0 | 32,6 | 0,3 | 1,0 | | 225 | | 315 | 36,8 | 32,7 | 35,7 | 32,4 |
| 15:50 | 33,9 | 32,4 | 43,2 | 45,7 | 33,6 | 32,4 | 0,3 | 1,1 | | 270 | | 315 | 32,9 | 32,6 | 33,7 | 32,2 |
| 15:55 | 33,5 | 32,4 | 43,3 | 45,8 | 34,1 | 32,3 | 0,2 | 0,2 | | 0 | | 315 | 34,9 | 32,3 | 34,5 | 32,6 |
| 16:00 | 33,8 | 32,3 | 42,9 | 45,9 | 36,0 | 32,9 | 1,3 | 0,9 | | 315 | | 315 | 44,9 | 34,9 | 38,3 | 33,2 |
| 16:05 | 34,2 | 32,5 | 43,1 | 46,2 | 37,1 | 32,6 | 1,8 | 0,2 | | 315 | | 315 | 51,7 | 32,9 | 41,0 | 33,0 |
| 16:10 | 34,6 | 32,5 | 41,6 | 45,3 | 36,3 | 32,7 | 0,7 | 1,0 | | 0 | | 315 | 41,6 | 33,5 | 38,0 | 32,7 |
| 16:15 | 34,2 | 32,5 | 41,8 | 45,1 | 35,6 | 32,5 | 0,3 | 0,8 | | 180 | | 225 | 38,3 | 32,5 | 36,6 | 32,4 |
| 16:20 | 34,2 | 32,4 | 41,7 | 44,6 | 36,0 | 32,5 | 2,1 | 0,8 | | 180 | | 225 | 46,0 | 32,8 | 38,5 | 32,4 |
| 16:25 | 34,3 | 32,5 | 40,5 | 44,0 | 35,3 | 32,7 | 1,1 | 0,4 | | 0 | | 315 | 39,4 | 33,1 | 36,5 | 32,9 |
| 16:30 | 34,1 | 32,6 | 39,7 | 42,1 | 35,2 | 32,8 | 2,0 | 1,5 | | 0 | | 315 | 41,0 | 34,0 | 36,5 | 32,7 |
| 16:35 | 34,7 | 32,8 | 39,5 | 43,3 | 37,2 | 33,2 | 0,2 | 0,4 | | 225 | | 315 | 41,0 | 34,2 | 38,3 | 33,6 |
| 16:40 | 34,6 | 32,8 | 40,0 | 43,2 | 35,2 | 32,9 | 1,5 | 0,4 | | 0 | | 315 | 37,8 | 33,0 | 36,0 | 33,0 |
| 16:45 | 34,8 | 32,8 | 40,6 | 44,4 | 36,8 | 32,8 | 1,4 | 0,9 | | 0 | | 315 | 45,4 | 32,8 | 39,3 | 32,7 |
| 16:50 | 34,5 | 32,5 | 41,3 | 45,3 | 34,7 | 32,3 | 1,8 | 0,6 | | 315 | | 315 | 35,9 | 32,0 | 35,2 | 32,3 |
| 16:55 | 34,2 | 32,5 | 41,5 | 45,0 | 33,7 | 32,6 | 0,2 | 0,3 | | 180 | | 315 | 33,0 | 32,7 | 33,9 | 32,8 |
| 17:00 | 33,7 | 32,4 | 43,2 | 45,6 | 33,1 | 32,6 | 2,7 | 0,6 | | 270 | | 315 | 28,9 | 33,1 | 32,0 | 32,7 |
| **Liberdade Square – 08th July 2013** | | | | | | | | | | | | | | | | |
|  | **P1** | **P2** | **P1** | **P2** | **P1** | **P2** | **P1** | | **P2** | | **P1** | **P2** | **P1** | **P2** | **P1** | **P2** |
| **Time** | **Ta**  **(°C)** | **Ta**  **(°C)** | **RH**  **(%)** | **RH**  **(%)** | **Tg**  **(°C)** | **Tg**  **(°C)** | **WS**  **(m/s)** | | **WS**  **(m/s)** | | **WD**  **(°)** | **WD**  **(°)** | **Tmrt**  **(°C)** | **Tmrt**  **(°C)** | **PET**  **(°C)** | **PET**  **(°C)** |
| 7:00 | 15,9 | 17,3 | 71,7 | 67,6 | 17,8 | 17,1 | 0,0 | 0,1 | | 45,0 | | 0,0 | 18,3 | 16,9 | 19,0 | 17,2 |
| 7:05 | 15,7 | 17,1 | 73,2 | 69,3 | 17,8 | 17,2 | 0,0 | 0,1 | | 315,0 | | 180,0 | 19,1 | 17,3 | 19,4 | 17,3 |
| 7:10 | 15,6 | 16,9 | 73,3 | 69,9 | 17,8 | 17,2 | 0,0 | 0,2 | | 270,0 | | 180,0 | 19,2 | 17,7 | 19,4 | 16,6 |
| 7:15 | 15,5 | 16,8 | 74,0 | 70,4 | 17,8 | 17,3 | 0,0 | 0,0 | | 315,0 | | 90,0 | 18,5 | 17,5 | 18,9 | 18,9 |
| 7:20 | 15,7 | 16,7 | 75,8 | 72,6 | 17,9 | 17,3 | 0,0 | 0,2 | | 45,0 | | 225,0 | 18,6 | 18,2 | 19,1 | 16,8 |
| 7:25 | 15,7 | 16,8 | 75,0 | 74,9 | 18,0 | 17,3 | 0,0 | 0,0 | | 315,0 | | 45,0 | 18,0 | 17,6 | 18,7 | 19,0 |
| 7:30 | 15,6 | 16,9 | 76,3 | 73,1 | 18,0 | 17,7 | 0,0 | 0,1 | | 270,0 | | 270,0 | 18,0 | 18,5 | 18,7 | 17,8 |
| 7:35 | 15,7 | 17,0 | 76,4 | 73,6 | 18,1 | 18,0 | 0,0 | 0,2 | | 315,0 | | 270,0 | 18,1 | 19,4 | 18,8 | 17,5 |
| 7:40 | 15,7 | 17,2 | 77,2 | 74,3 | 18,2 | 17,9 | 0,0 | 0,2 | | 315,0 | | 45,0 | 18,9 | 19,2 | 19,3 | 17,5 |
| 7:45 | 15,9 | 17,5 | 80,6 | 77,0 | 18,4 | 18,5 | 0,0 | 0,1 | | 90,0 | | 90,0 | 19,0 | 19,8 | 19,5 | 18,8 |
| 7:50 | 16,2 | 17,9 | 81,0 | 75,7 | 18,5 | 19,1 | 0,0 | 0,3 | | 45,0 | | 45,0 | 19,5 | 21,6 | 19,9 | 18,5 |
| 7:55 | 16,7 | 17,9 | 78,2 | 75,3 | 18,7 | 19,1 | 0,0 | 0,6 | | 45,0 | | 0,0 | 19,3 | 22,7 | 19,9 | 17,6 |
| 8:00 | 17,2 | 18,1 | 76,0 | 73,6 | 18,9 | 19,1 | 0,7 | 0,7 | | 180,0 | | 0,0 | 25,2 | 22,6 | 17,7 | 17,4 |
| 8:05 | 17,4 | 18,3 | 74,3 | 71,3 | 19,1 | 19,3 | 0,4 | 0,9 | | 180,0 | | 0,0 | 23,1 | 23,0 | 18,2 | 17,1 |
| 8:10 | 17,6 | 18,5 | 71,1 | 69,7 | 19,2 | 19,3 | 0,5 | 1,0 | | 0,0 | | 45,0 | 23,8 | 23,0 | 18,2 | 17,0 |
| 8:15 | 17,7 | 18,5 | 71,2 | 71,3 | 19,3 | 19,4 | 0,2 | 0,7 | | 0,0 | | 0,0 | 21,7 | 22,5 | 19,0 | 17,6 |
| 8:20 | 17,7 | 18,5 | 71,6 | 68,6 | 19,2 | 19,4 | 0,6 | 0,8 | | 180,0 | | 0,0 | 24,1 | 22,8 | 18,0 | 17,4 |
| 8:25 | 17,7 | 18,7 | 70,3 | 66,3 | 19,3 | 19,5 | 0,4 | 0,5 | | 0,0 | | 0,0 | 23,4 | 21,6 | 18,5 | 18,0 |
| 8:30 | 17,8 | 19,5 | 69,8 | 65,1 | 19,4 | 22,2 | 0,3 | 0,4 | | 45,0 | | 45,0 | 22,9 | 29,0 | 18,9 | 22,3 |
| 8:35 | 17,9 | 20,0 | 70,3 | 64,8 | 19,6 | 25,7 | 0,5 | 0,5 | | 45,0 | | 45,0 | 24,0 | 40,1 | 18,5 | 27,5 |
| 8:40 | 18,2 | 20,3 | 71,0 | 63,4 | 20,7 | 24,4 | 0,7 | 0,6 | | 0,0 | | 90,0 | 29,3 | 36,8 | 20,0 | 25,5 |
| 8:45 | 18,7 | 20,2 | 68,8 | 64,3 | 22,1 | 23,0 | 0,3 | 0,5 | | 315,0 | | 45,0 | 28,7 | 30,4 | 22,3 | 22,9 |
| 8:50 | 19,4 | 19,9 | 67,2 | 65,6 | 23,9 | 21,4 | 1,2 | 0,6 | | 0,0 | | 45,0 | 42,8 | 26,0 | 24,9 | 20,3 |
| 8:55 | 19,8 | 19,9 | 65,2 | 65,8 | 24,7 | 20,8 | 0,8 | 0,5 | | 0,0 | | 45,0 | 41,3 | 23,6 | 26,3 | 19,6 |
| 9:00 | 20,1 | 19,9 | 65,4 | 65,5 | 24,8 | 20,6 | 0,8 | 0,9 | | 0,0 | | 90,0 | 40,8 | 23,4 | 26,3 | 18,4 |
| 9:05 | 20,4 | 20,2 | 63,2 | 65,0 | 24,5 | 20,6 | 0,4 | 0,9 | | 45,0 | | 90,0 | 34,7 | 22,4 | 25,6 | 18,3 |
| 9:10 | 20,6 | 20,6 | 62,6 | 63,4 | 24,7 | 20,8 | 0,2 | 0,2 | | 315,0 | | 90,0 | 30,5 | 21,2 | 25,0 | 20,4 |
| 9:15 | 20,7 | 20,4 | 62,8 | 63,9 | 24,5 | 20,8 | 0,5 | 0,3 | | 315,0 | | 45,0 | 34,5 | 21,6 | 25,2 | 20,0 |
| 9:20 | 21,1 | 20,3 | 61,4 | 63,9 | 24,1 | 20,8 | 0,9 | 0,3 | | 0,0 | | 0,0 | 34,8 | 21,8 | 23,9 | 20,0 |
| 9:25 | 21,5 | 20,3 | 61,5 | 64,7 | 23,7 | 20,8 | 0,3 | 0,7 | | 0,0 | | 0,0 | 28,1 | 22,3 | 23,7 | 18,8 |
| 9:30 | 21,6 | 20,4 | 60,6 | 64,3 | 23,3 | 21,0 | 0,5 | 0,6 | | 180,0 | | 270,0 | 27,7 | 22,6 | 22,7 | 19,2 |
| 9:35 | 21,7 | 20,5 | 61,1 | 64,9 | 23,4 | 21,1 | 0,7 | 0,1 | | 0,0 | | 270,0 | 29,1 | 21,5 | 22,6 | 21,2 |
| 9:40 | 22,1 | 20,6 | 60,8 | 65,2 | 23,8 | 21,2 | 0,2 | 0,2 | | 0,0 | | 0,0 | 27,0 | 22,1 | 24,1 | 20,9 |
| 9:45 | 22,4 | 20,7 | 59,3 | 64,7 | 24,5 | 21,3 | 0,1 | 0,4 | | 0,0 | | 45,0 | 27,1 | 22,8 | 25,0 | 20,3 |
| 9:50 | 22,8 | 21,0 | 59,6 | 64,9 | 25,6 | 21,5 | 0,8 | 2,0 | | 45,0 | | 0,0 | 35,0 | 24,8 | 25,8 | 18,1 |
| 9:55 | 22,7 | 21,1 | 59,4 | 64,5 | 25,8 | 21,5 | 0,5 | 0,7 | | 0,0 | | 0,0 | 33,4 | 23,0 | 26,1 | 19,6 |
| 10:00 | 22,6 | 21,2 | 60,5 | 64,1 | 25,5 | 22,2 | 0,7 | 2,4 | | 270,0 | | 0,0 | 34,8 | 29,3 | 25,9 | 19,0 |
| 10:05 | 22,7 | 21,4 | 59,3 | 63,9 | 25,4 | 24,4 | 0,3 | 1,8 | | 0,0 | | 45,0 | 30,8 | 40,6 | 25,8 | 24,1 |
| 10:10 | 22,7 | 21,5 | 59,5 | 63,2 | 25,5 | 24,6 | 2,0 | 0,8 | | 0,0 | | 90,0 | 41,8 | 35,3 | 25,4 | 24,9 |
| 10:15 | 23,1 | 21,6 | 59,1 | 63,7 | 25,8 | 23,9 | 1,1 | 1,2 | | 225,0 | | 180,0 | 37,3 | 34,0 | 26,1 | 23,1 |
| 10:20 | 23,3 | 21,8 | 58,8 | 63,4 | 26,3 | 23,2 | 0,2 | 1,4 | | 315,0 | | 180,0 | 30,2 | 30,2 | 26,4 | 21,3 |
| 10:25 | 23,7 | 21,9 | 58,3 | 62,6 | 27,0 | 22,8 | 0,3 | 2,1 | | 315,0 | | 180,0 | 33,2 | 28,2 | 27,6 | 19,7 |
| 10:30 | 23,6 | 22,1 | 57,3 | 61,7 | 27,1 | 22,8 | 0,4 | 0,4 | | 0,0 | | 180,0 | 34,6 | 24,6 | 27,8 | 22,0 |
| 10:35 | 24,1 | 22,2 | 56,7 | 63,2 | 27,7 | 22,8 | 2,4 | 0,5 | | 0,0 | | 135,0 | 50,2 | 24,7 | 29,3 | 21,8 |
| 10:40 | 23,9 | 22,3 | 58,1 | 62,9 | 27,6 | 22,9 | 0,3 | 1,0 | | 0,0 | | 90,0 | 34,1 | 25,3 | 28,2 | 20,8 |
| 10:45 | 24,2 | 22,4 | 56,5 | 63,0 | 27,7 | 22,9 | 0,5 | 0,4 | | 45,0 | | 135,0 | 36,5 | 24,3 | 28,6 | 22,1 |
| 10:50 | 24,5 | 22,4 | 55,6 | 62,5 | 28,0 | 22,9 | 1,4 | 0,0 | | 0,0 | | 180,0 | 44,5 | 23,2 | 29,6 | 24,2 |
| 10:55 | 24,8 | 22,6 | 54,5 | 61,1 | 28,1 | 23,2 | 0,3 | 0,2 | | 90,0 | | 90,0 | 35,0 | 24,3 | 29,2 | 23,1 |
| 11:00 | 25,3 | 22,8 | 53,5 | 60,8 | 28,7 | 23,4 | 2,7 | 0,5 | | 0,0 | | 270,0 | 50,6 | 25,1 | 30,2 | 22,4 |
| 11:05 | 25,4 | 23,1 | 51,6 | 58,2 | 29,0 | 23,6 | 0,9 | 1,3 | | 135,0 | | 90,0 | 41,7 | 25,8 | 30,5 | 21,1 |
| 11:10 | 25,8 | 22,9 | 54,8 | 57,7 | 29,2 | 23,7 | 1,5 | 0,9 | | 180,0 | | 90,0 | 45,2 | 26,7 | 30,9 | 22,0 |
| 11:15 | 25,8 | 22,9 | 50,1 | 57,0 | 29,1 | 23,8 | 1,3 | 0,5 | | 45,0 | | 0,0 | 43,8 | 26,2 | 30,7 | 22,9 |
| 11:20 | 25,0 | 22,8 | 50,4 | 56,1 | 28,5 | 23,6 | 1,6 | 0,4 | | 315,0 | | 0,0 | 46,0 | 25,8 | 30,2 | 23,0 |
| 11:25 | 24,4 | 22,9 | 52,8 | 55,4 | 27,9 | 23,6 | 1,4 | 0,5 | | 0,0 | | 0,0 | 43,9 | 25,6 | 29,2 | 22,6 |
| 11:30 | 24,6 | 23,1 | 50,5 | 55,1 | 28,0 | 23,8 | 0,6 | 0,8 | | 0,0 | | 45,0 | 37,1 | 26,5 | 28,8 | 22,3 |
| 11:35 | 24,8 | 23,2 | 48,3 | 52,9 | 27,9 | 23,8 | 0,2 | 0,8 | | 45,0 | | 45,0 | 32,4 | 26,4 | 28,3 | 22,3 |
| 11:40 | 25,4 | 23,3 | 46,9 | 53,3 | 28,5 | 24,0 | 1,2 | 0,8 | | 45,0 | | 90,0 | 41,6 | 26,7 | 29,6 | 22,5 |
| 11:45 | 26,1 | 23,5 | 44,3 | 48,8 | 29,3 | 25,4 | 0,9 | 1,0 | | 45,0 | | 90,0 | 40,4 | 33,1 | 30,4 | 24,8 |
| 11:50 | 26,6 | 23,7 | 44,2 | 50,3 | 29,6 | 25,9 | 0,5 | 1,0 | | 225,0 | | 180,0 | 37,3 | 34,2 | 30,6 | 25,5 |
| 11:55 | 26,7 | 23,8 | 43,8 | 50,8 | 29,9 | 25,2 | 0,4 | 0,4 | | 135,0 | | 135,0 | 36,7 | 28,4 | 30,7 | 24,8 |
| 12:00 | 26,3 | 23,8 | 47,7 | 51,8 | 29,6 | 24,8 | 0,1 | 0,7 | | 135,0 | | 135,0 | 33,5 | 28,0 | 30,3 | 23,7 |
| 12:05 | 26,2 | 23,9 | 45,1 | 50,1 | 29,7 | 24,7 | 0,5 | 0,7 | | 270,0 | | 90,0 | 38,7 | 27,2 | 31,0 | 23,4 |
| 12:10 | 25,9 | 24,0 | 45,9 | 50,9 | 29,2 | 25,2 | 1,6 | 0,6 | | 225,0 | | 90,0 | 45,2 | 28,7 | 30,6 | 24,5 |
| 12:15 | 25,9 | 24,0 | 45,1 | 49,4 | 28,9 | 25,3 | 1,4 | 0,3 | | 315,0 | | 225,0 | 42,7 | 27,8 | 30,0 | 25,1 |
| 12:20 | 26,2 | 24,1 | 44,4 | 49,5 | 29,2 | 25,0 | 0,2 | 1,0 | | 315,0 | | 180,0 | 34,1 | 28,7 | 30,0 | 23,5 |
| 12:25 | 26,4 | 24,0 | 45,5 | 51,4 | 29,1 | 24,7 | 2,6 | 1,0 | | 0,0 | | 180,0 | 47,0 | 27,9 | 30,1 | 23,2 |
| 12:30 | 26,1 | 24,0 | 46,7 | 50,1 | 29,0 | 24,7 | 0,2 | 1,1 | | 0,0 | | 90,0 | 32,9 | 27,7 | 29,3 | 22,9 |
| 12:35 | 25,5 | 23,8 | 47,2 | 49,5 | 28,8 | 24,7 | 1,2 | 1,0 | | 45,0 | | 45,0 | 42,8 | 28,5 | 30,2 | 23,2 |
| 12:40 | 25,9 | 24,0 | 50,8 | 50,3 | 29,1 | 24,7 | 3,6 | 2,2 | | 45,0 | | 0,0 | 53,3 | 29,6 | 30,5 | 21,9 |
| 12:45 | 25,6 | 24,0 | 51,6 | 48,6 | 28,8 | 24,7 | 0,2 | 0,7 | | 45,0 | | 45,0 | 34,1 | 26,9 | 29,7 | 23,4 |
| 12:50 | 26,1 | 24,2 | 42,9 | 46,4 | 29,2 | 24,8 | 0,3 | 0,8 | | 270,0 | | 0,0 | 35,1 | 27,0 | 29,9 | 23,3 |
| 12:55 | 26,4 | 24,2 | 41,7 | 47,1 | 29,5 | 24,9 | 0,2 | 0,8 | | 270,0 | | 270,0 | 34,1 | 27,5 | 30,0 | 23,5 |
| 13:00 | 26,7 | 24,4 | 40,5 | 47,0 | 29,7 | 25,5 | 0,1 | 0,8 | | 0,0 | | 270,0 | 33,3 | 29,3 | 30,3 | 24,5 |
| 13:05 | 26,2 | 24,4 | 43,0 | 46,2 | 29,4 | 25,4 | 1,4 | 1,0 | | 0,0 | | 90,0 | 44,1 | 29,7 | 30,9 | 24,2 |
| 13:10 | 26,6 | 24,5 | 43,6 | 47,0 | 29,7 | 25,4 | 0,6 | 1,9 | | 315,0 | | 90,0 | 38,1 | 30,5 | 30,6 | 23,1 |
| 13:15 | 26,9 | 24,3 | 42,3 | 47,5 | 29,8 | 25,2 | 0,3 | 0,1 | | 0,0 | | 90,0 | 35,6 | 26,1 | 30,7 | 25,4 |
| 13:20 | 27,0 | 24,5 | 41,1 | 46,4 | 30,0 | 25,6 | 0,6 | 0,5 | | 315,0 | | 90,0 | 38,2 | 28,7 | 30,9 | 25,1 |
| 13:25 | 26,5 | 24,7 | 42,2 | 47,7 | 29,5 | 26,0 | 2,7 | 0,1 | | 0,0 | | 180,0 | 49,5 | 27,4 | 31,0 | 26,3 |
| 13:30 | 27,0 | 24,9 | 41,4 | 47,1 | 29,8 | 26,2 | 3,9 | 0,4 | | 315,0 | | 90,0 | 52,0 | 28,9 | 30,9 | 25,8 |
| 13:35 | 26,8 | 24,8 | 40,2 | 45,1 | 29,6 | 26,1 | 0,4 | 1,2 | | 0,0 | | 90,0 | 36,0 | 31,6 | 30,4 | 24,9 |
| 13:40 | 27,1 | 24,7 | 40,6 | 43,8 | 30,0 | 27,5 | 0,4 | 0,3 | | 90,0 | | 0,0 | 36,2 | 33,2 | 30,7 | 28,1 |
| 13:45 | 27,4 | 24,9 | 40,2 | 45,0 | 30,6 | 28,2 | 0,8 | 1,4 | | 315,0 | | 90,0 | 41,3 | 43,7 | 32,1 | 29,5 |
| 13:50 | 27,1 | 25,0 | 46,1 | 45,5 | 30,1 | 27,1 | 1,5 | 1,2 | | 135,0 | | 45,0 | 44,2 | 36,3 | 31,6 | 27,0 |
| 13:55 | 27,3 | 25,1 | 42,7 | 43,5 | 30,3 | 26,6 | 1,3 | 1,1 | | 135,0 | | 90,0 | 43,4 | 32,8 | 31,8 | 25,8 |
| 14:00 | 26,9 | 25,1 | 41,0 | 43,7 | 30,1 | 26,3 | 0,3 | 1,9 | | 315,0 | | 90,0 | 35,6 | 33,1 | 30,7 | 24,6 |
| 14:05 | 27,1 | 25,1 | 40,7 | 44,2 | 30,0 | 26,4 | 1,6 | 1,8 | | 315,0 | | 90,0 | 44,6 | 33,6 | 31,5 | 24,9 |
| 14:10 | 26,9 | 24,9 | 39,8 | 46,5 | 29,9 | 26,2 | 2,6 | 1,9 | | 315,0 | | 90,0 | 48,7 | 33,6 | 31,2 | 24,6 |
| 14:15 | 27,2 | 25,1 | 40,0 | 45,0 | 30,0 | 26,3 | 0,5 | 1,1 | | 45,0 | | 270,0 | 37,1 | 31,4 | 30,8 | 25,2 |
| 14:20 | 27,4 | 25,5 | 42,0 | 43,7 | 30,3 | 26,6 | 0,7 | 1,8 | | 45,0 | | 270,0 | 39,0 | 32,9 | 31,3 | 25,1 |
| 14:25 | 27,0 | 25,6 | 38,9 | 42,6 | 30,0 | 27,5 | 2,5 | 1,0 | | 315,0 | | 0,0 | 48,9 | 34,7 | 31,5 | 27,2 |
| 14:30 | 27,2 | 25,3 | 40,3 | 42,3 | 30,0 | 26,9 | 2,3 | 1,1 | | 0,0 | | 0,0 | 46,9 | 33,5 | 31,3 | 26,2 |
| 14:35 | 26,9 | 25,2 | 39,7 | 43,3 | 29,6 | 26,5 | 0,5 | 1,1 | | 135,0 | | 90,0 | 36,8 | 32,0 | 30,5 | 25,6 |
| 14:40 | 27,0 | 25,2 | 39,6 | 43,2 | 29,8 | 26,5 | 0,2 | 0,8 | | 225,0 | | 90,0 | 33,8 | 31,2 | 30,2 | 25,9 |
| 14:45 | 27,4 | 25,6 | 41,3 | 44,4 | 30,1 | 26,8 | 1,2 | 1,0 | | 135,0 | | 270,0 | 41,8 | 31,7 | 31,4 | 26,0 |
| 14:50 | 27,6 | 25,7 | 39,8 | 43,3 | 30,3 | 26,8 | 0,3 | 0,7 | | 315,0 | | 45,0 | 35,4 | 30,5 | 31,0 | 26,2 |
| 14:55 | 27,5 | 25,7 | 38,8 | 42,3 | 30,2 | 26,8 | 2,3 | 0,8 | | 135,0 | | 45,0 | 46,7 | 30,5 | 31,5 | 26,0 |
| 15:00 | 27,4 | 25,5 | 39,7 | 46,1 | 29,8 | 27,0 | 0,4 | 0,6 | | 135,0 | | 0,0 | 35,1 | 31,3 | 30,3 | 26,7 |
| 15:05 | 27,7 | 25,6 | 39,8 | 44,9 | 29,4 | 27,0 | 0,8 | 0,8 | | 0,0 | | 0,0 | 35,3 | 31,4 | 29,6 | 26,3 |
| 15:10 | 27,5 | 25,8 | 40,9 | 44,7 | 28,6 | 27,1 | 0,3 | 0,7 | | 90,0 | | 180,0 | 30,6 | 31,3 | 28,5 | 26,6 |
| 15:15 | 27,4 | 25,8 | 40,9 | 44,1 | 29,0 | 27,1 | 0,4 | 0,8 | | 135,0 | | 45,0 | 32,4 | 31,8 | 29,1 | 26,6 |
| 15:20 | 26,8 | 25,5 | 41,9 | 44,2 | 27,0 | 26,3 | 0,6 | 0,7 | | 90,0 | | 270,0 | 27,7 | 28,6 | 26,0 | 25,2 |
| 15:25 | 26,0 | 24,9 | 44,2 | 47,7 | 25,5 | 25,5 | 0,3 | 0,8 | | 225,0 | | 90,0 | 24,3 | 27,5 | 24,6 | 24,1 |
| 15:30 | 25,3 | 24,8 | 46,4 | 47,5 | 24,5 | 25,1 | 0,6 | 1,6 | | 270,0 | | 180,0 | 22,1 | 26,7 | 22,6 | 22,4 |
| 15:35 | 24,8 | 24,6 | 46,7 | 47,4 | 24,0 | 24,8 | 0,2 | 1,0 | | 0,0 | | 180,0 | 22,9 | 25,8 | 23,6 | 22,8 |
| 15:40 | 24,8 | 24,5 | 47,2 | 47,4 | 23,8 | 24,7 | 0,6 | 1,5 | | 0,0 | | 135,0 | 20,6 | 25,6 | 21,6 | 21,9 |
| 15:45 | 24,7 | 24,4 | 47,0 | 48,0 | 23,6 | 24,5 | 0,0 | 1,4 | | 90,0 | | 135,0 | 23,4 | 25,4 | 25,0 | 21,9 |
| 15:50 | 24,7 | 24,5 | 47,8 | 49,9 | 23,6 | 24,9 | 0,8 | 0,3 | | 0,0 | | 0,0 | 19,5 | 25,7 | 20,8 | 24,4 |
| 15:55 | 24,7 | 24,9 | 47,2 | 48,2 | 23,7 | 26,8 | 0,5 | 0,8 | | 315,0 | | 135,0 | 20,6 | 33,7 | 21,7 | 26,8 |
| 16:00 | 24,9 | 25,0 | 47,0 | 47,2 | 23,8 | 26,2 | 0,1 | 0,7 | | 0,0 | | 135,0 | 22,8 | 29,9 | 24,0 | 25,4 |
| 16:05 | 25,0 | 25,0 | 46,7 | 48,2 | 24,0 | 25,8 | 0,4 | 0,4 | | 135,0 | | 90,0 | 21,3 | 27,7 | 22,4 | 25,3 |
| 16:10 | 25,0 | 24,8 | 46,9 | 46,9 | 24,2 | 25,5 | 0,2 | 1,1 | | 0,0 | | 90,0 | 23,2 | 28,1 | 23,8 | 23,7 |
| 16:15 | 25,2 | 24,8 | 46,1 | 46,8 | 24,6 | 25,2 | 0,3 | 1,2 | | 135,0 | | 135,0 | 23,3 | 26,8 | 23,7 | 23,0 |
| 16:20 | 25,0 | 24,7 | 47,4 | 48,3 | 24,5 | 25,0 | 0,7 | 0,6 | | 45,0 | | 90,0 | 22,6 | 25,9 | 22,4 | 23,8 |
| 16:25 | 25,1 | 24,7 | 47,5 | 48,9 | 24,5 | 24,9 | 1,1 | 0,6 | | 0,0 | | 135,0 | 21,7 | 25,7 | 21,6 | 23,7 |
| 16:30 | 24,8 | 24,4 | 52,3 | 53,8 | 24,1 | 24,8 | 0,1 | 0,5 | | 225,0 | | 135,0 | 23,3 | 25,9 | 24,2 | 23,9 |
| 16:35 | 24,7 | 24,2 | 53,2 | 55,6 | 23,9 | 24,6 | 0,2 | 1,4 | | 0,0 | | 315,0 | 22,8 | 26,5 | 23,5 | 22,2 |
| 16:40 | 24,5 | 24,0 | 54,0 | 56,9 | 23,6 | 24,5 | 1,4 | 1,2 | | 0,0 | | 315,0 | 19,3 | 26,6 | 19,9 | 22,4 |
| 16:45 | 24,4 | 24,0 | 54,7 | 55,7 | 23,5 | 24,3 | 0,5 | 0,8 | | 180,0 | | 135,0 | 21,2 | 25,6 | 21,8 | 22,7 |
| 16:50 | 24,2 | 24,0 | 54,6 | 55,1 | 23,4 | 24,3 | 0,2 | 0,2 | | 315,0 | | 0,0 | 21,9 | 24,7 | 22,8 | 24,1 |
| 16:55 | 24,1 | 23,9 | 54,7 | 55,5 | 23,3 | 24,1 | 0,4 | 0,1 | | 315,0 | | 0,0 | 21,0 | 24,4 | 21,8 | 24,4 |
| 17:00 | 24,1 | 23,8 | 54,9 | 56,6 | 23,1 | 23,9 | 1,6 | 0,3 | | 315,0 | | 90,0 | 17,9 | 24,1 | 19,1 | 23,3 |
| **Sete de Setembro Square – 09th July 2013** | | | | | | | | | | | | | | | | |
|  | **P3** | **P4** | **P3** | **P4** | **P3** | **P4** | **P3** | | **P4** | | **P3** | **P4** | **P3** | **P4** | **P3** | **P4** |
| **Time** | **Ta**  **(°C)** | **Ta**  **(°C)** | **RH**  **(%)** | **RH**  **(%)** | **Tg**  **(°C)** | **Tg**  **(°C)** | **WS**  **(m/s)** | | **WS**  **(m/s)** | | **WD**  **(°)** | **WD**  **(°)** | **Tmrt**  **(°C)** | **Tmrt**  **(°C)** | **PET**  **(°C)** | **PET**  **(°C)** |
| 7:00 | 17,6 | 17,4 | 61,1 | 62,1 | 18,1 | 17,6 | 1,1 | 0,2 | | 0,0 | | 135,0 | 20,7 | 17,9 | 15,3 | 16,9 |
| 7:05 | 17,6 | 17,5 | 61,5 | 61,5 | 18,0 | 17,7 | 1,8 | 0,4 | | 180,0 | | 135,0 | 20,5 | 18,2 | 14,3 | 16,2 |
| 7:10 | 17,5 | 17,5 | 61,1 | 61,5 | 18,0 | 17,8 | 0,8 | 0,3 | | 180,0 | | 135,0 | 19,8 | 18,3 | 15,6 | 16,6 |
| 7:15 | 17,6 | 17,7 | 60,9 | 61,4 | 18,1 | 18,0 | 0,7 | 0,2 | | 135,0 | | 135,0 | 20,0 | 18,5 | 16,0 | 17,4 |
| 7:20 | 17,7 | 17,7 | 61,1 | 61,0 | 18,4 | 18,0 | 1,4 | 0,4 | | 135,0 | | 135,0 | 21,9 | 18,7 | 15,3 | 16,5 |
| 7:25 | 17,9 | 17,8 | 60,2 | 61,4 | 18,6 | 18,1 | 1,3 | 0,4 | | 180,0 | | 135,0 | 22,1 | 18,8 | 15,6 | 16,6 |
| 7:30 | 18,0 | 17,8 | 60,4 | 61,4 | 18,5 | 18,1 | 2,6 | 0,2 | | 180,0 | | 135,0 | 22,7 | 18,6 | 14,4 | 17,5 |
| 7:35 | 18,1 | 17,9 | 60,6 | 61,7 | 18,8 | 18,2 | 1,9 | 0,3 | | 180,0 | | 135,0 | 23,6 | 18,9 | 15,4 | 17,1 |
| 7:40 | 18,0 | 17,9 | 60,6 | 61,4 | 18,6 | 18,2 | 0,2 | 0,3 | | 225,0 | | 135,0 | 19,6 | 18,8 | 18,1 | 17,1 |
| 7:45 | 18,0 | 18,0 | 60,3 | 60,7 | 18,5 | 18,2 | 1,4 | 0,3 | | 180,0 | | 135,0 | 21,1 | 18,8 | 15,3 | 17,2 |
| 7:50 | 18,0 | 18,0 | 60,5 | 60,9 | 18,7 | 18,2 | 1,1 | 0,1 | | 0,0 | | 135,0 | 21,8 | 18,5 | 16,0 | 18,3 |
| 7:55 | 18,2 | 18,0 | 60,7 | 61,2 | 18,9 | 18,3 | 1,2 | 0,2 | | 180,0 | | 135,0 | 22,8 | 18,7 | 16,3 | 17,6 |
| 8:00 | 18,4 | 18,1 | 60,1 | 61,5 | 21,9 | 18,3 | 1,3 | 0,3 | | 135,0 | | 135,0 | 38,3 | 18,7 | 21,6 | 17,2 |
| 8:05 | 18,7 | 18,1 | 59,9 | 61,8 | 22,3 | 18,4 | 0,3 | 0,3 | | 135,0 | | 135,0 | 29,4 | 18,9 | 22,5 | 17,3 |
| 8:10 | 19,0 | 18,1 | 58,9 | 61,8 | 22,2 | 18,4 | 1,9 | 0,1 | | 180,0 | | 135,0 | 40,7 | 18,8 | 21,3 | 18,5 |
| 8:15 | 19,1 | 18,2 | 59,2 | 61,8 | 22,6 | 18,5 | 1,5 | 0,2 | | 180,0 | | 135,0 | 39,9 | 19,0 | 22,2 | 17,9 |
| 8:20 | 19,2 | 18,3 | 59,2 | 61,8 | 22,1 | 18,6 | 1,9 | 0,2 | | 180,0 | | 135,0 | 39,1 | 19,2 | 20,9 | 18,1 |
| 8:25 | 19,1 | 18,3 | 59,2 | 61,6 | 22,8 | 18,6 | 2,6 | 0,5 | | 180,0 | | 135,0 | 47,5 | 19,4 | 22,3 | 16,8 |
| 8:30 | 19,5 | 18,4 | 58,4 | 61,4 | 24,4 | 18,6 | 1,2 | 0,2 | | 180,0 | | 135,0 | 45,5 | 19,1 | 26,1 | 18,1 |
| 8:35 | 20,0 | 18,5 | 57,3 | 62,0 | 23,8 | 18,9 | 1,3 | 0,5 | | 225,0 | | 135,0 | 40,6 | 20,2 | 24,0 | 17,3 |
| 8:40 | 19,9 | 18,5 | 58,3 | 61,4 | 23,6 | 18,9 | 0,3 | 0,3 | | 225,0 | | 135,0 | 31,2 | 19,7 | 24,2 | 17,9 |
| 8:45 | 20,0 | 18,6 | 57,6 | 61,4 | 23,4 | 19,0 | 1,5 | 0,7 | | 180,0 | | 135,0 | 40,6 | 20,4 | 23,4 | 16,8 |
| 8:50 | 20,3 | 18,7 | 57,5 | 61,4 | 23,8 | 19,0 | 1,2 | 0,4 | | 180,0 | | 135,0 | 39,5 | 19,9 | 24,2 | 17,7 |
| 8:55 | 20,1 | 18,8 | 58,0 | 61,4 | 24,0 | 19,1 | 2,8 | 0,6 | | 180,0 | | 135,0 | 50,7 | 20,1 | 24,2 | 17,1 |
| 9:00 | 20,6 | 18,8 | 56,7 | 61,5 | 24,9 | 19,2 | 0,5 | 0,2 | | 180,0 | | 135,0 | 35,5 | 19,7 | 25,5 | 18,6 |
| 9:05 | 21,1 | 19,0 | 55,7 | 61,3 | 25,9 | 19,4 | 0,2 | 0,7 | | 135,0 | | 135,0 | 34,1 | 20,8 | 27,1 | 17,3 |
| 9:10 | 20,8 | 19,1 | 56,5 | 61,0 | 23,9 | 19,3 | 2,0 | 0,3 | | 180,0 | | 135,0 | 41,8 | 20,0 | 23,3 | 18,4 |
| 9:15 | 20,8 | 19,2 | 56,3 | 60,7 | 25,2 | 19,6 | 0,2 | 0,2 | | 45,0 | | 135,0 | 32,5 | 20,3 | 26,1 | 19,1 |
| 9:20 | 21,4 | 19,3 | 54,6 | 60,5 | 24,8 | 19,9 | 1,6 | 0,2 | | 180,0 | | 135,0 | 42,5 | 21,1 | 25,2 | 19,5 |
| 9:25 | 21,6 | 19,4 | 54,1 | 59,8 | 26,1 | 19,8 | 1,1 | 0,4 | | 135,0 | | 135,0 | 43,8 | 20,7 | 27,6 | 18,4 |
| 9:30 | 21,9 | 19,5 | 53,4 | 59,9 | 25,0 | 20,1 | 2,4 | 0,3 | | 180,0 | | 135,0 | 44,9 | 21,3 | 24,8 | 19,2 |
| 9:35 | 21,7 | 19,7 | 53,2 | 59,0 | 25,0 | 20,1 | 0,8 | 0,7 | | 180,0 | | 135,0 | 36,2 | 21,7 | 25,3 | 18,1 |
| 9:40 | 22,0 | 19,7 | 52,8 | 58,7 | 26,0 | 20,1 | 0,5 | 0,7 | | 45,0 | | 135,0 | 36,9 | 21,5 | 27,2 | 18,0 |
| 9:45 | 22,2 | 19,9 | 52,7 | 58,8 | 25,4 | 20,4 | 0,8 | 0,4 | | 135,0 | | 135,0 | 37,0 | 21,5 | 26,1 | 19,1 |
| 9:50 | 22,1 | 20,0 | 52,8 | 58,3 | 25,4 | 20,3 | 0,5 | 0,3 | | 0,0 | | 135,0 | 33,9 | 20,9 | 25,8 | 19,3 |
| 9:55 | 22,3 | 20,0 | 52,7 | 58,4 | 25,3 | 20,1 | 0,9 | 0,3 | | 135,0 | | 135,0 | 36,3 | 20,2 | 25,5 | 19,0 |
| 10:00 | 22,0 | 20,1 | 53,8 | 59,2 | 22,1 | 20,6 | 1,5 | 0,1 | | 0,0 | | 315,0 | 22,7 | 20,9 | 18,8 | 20,6 |
| 10:05 | 21,6 | 20,3 | 54,8 | 58,8 | 21,9 | 20,9 | 0,6 | 0,0 | | 135,0 | | 90,0 | 23,1 | 21,1 | 20,2 | 22,2 |
| 10:10 | 21,5 | 20,3 | 55,3 | 58,5 | 22,1 | 20,4 | 0,5 | 0,1 | | 0,0 | | 315,0 | 23,7 | 20,6 | 20,8 | 20,6 |
| 10:15 | 21,5 | 20,3 | 55,7 | 58,5 | 22,2 | 20,6 | 0,3 | 0,4 | | 0,0 | | 135,0 | 23,9 | 21,4 | 21,7 | 19,3 |
| 10:20 | 21,5 | 20,4 | 55,4 | 58,9 | 22,2 | 20,7 | 0,8 | 0,1 | | 180,0 | | 135,0 | 25,0 | 21,2 | 20,4 | 20,9 |
| 10:25 | 21,5 | 20,4 | 55,8 | 58,5 | 22,1 | 20,7 | 0,2 | 0,3 | | 90,0 | | 45,0 | 23,1 | 21,3 | 21,8 | 19,8 |
| 10:30 | 21,6 | 20,4 | 55,7 | 58,9 | 22,4 | 20,9 | 0,5 | 0,2 | | 0,0 | | 135,0 | 24,6 | 21,6 | 21,2 | 20,4 |
| 10:35 | 21,7 | 20,5 | 55,5 | 58,7 | 23,3 | 21,0 | 1,4 | 0,1 | | 135,0 | | 135,0 | 31,1 | 21,5 | 21,5 | 21,1 |
| 10:40 | 21,8 | 20,6 | 55,5 | 59,3 | 22,7 | 21,1 | 1,5 | 0,1 | | 45,0 | | 90,0 | 27,3 | 21,5 | 20,0 | 21,2 |
| 10:45 | 21,5 | 20,7 | 55,9 | 58,6 | 22,4 | 21,5 | 0,3 | 0,3 | | 0,0 | | 135,0 | 24,1 | 23,1 | 21,8 | 20,8 |
| 10:50 | 21,6 | 20,8 | 55,8 | 58,1 | 22,5 | 21,3 | 1,8 | 0,3 | | 0,0 | | 135,0 | 27,8 | 22,3 | 19,6 | 20,5 |
| 10:55 | 22,0 | 20,8 | 55,2 | 58,5 | 24,7 | 21,2 | 0,7 | 0,2 | | 135,0 | | 135,0 | 33,2 | 21,9 | 24,6 | 20,8 |
| 11:00 | 22,6 | 20,8 | 53,4 | 58,4 | 26,0 | 21,1 | 0,8 | 0,2 | | 45,0 | | 90,0 | 37,8 | 21,7 | 26,8 | 20,7 |
| 11:05 | 23,1 | 20,9 | 52,0 | 58,2 | 26,3 | 21,5 | 0,4 | 0,1 | | 45,0 | | 90,0 | 33,2 | 22,3 | 26,6 | 21,7 |
| 11:10 | 23,4 | 21,0 | 50,8 | 57,3 | 26,1 | 21,4 | 0,4 | 0,3 | | 90,0 | | 135,0 | 32,2 | 22,2 | 26,4 | 20,6 |
| 11:15 | 23,5 | 21,2 | 50,6 | 56,4 | 25,2 | 21,7 | 1,5 | 0,3 | | 270,0 | | 90,0 | 34,0 | 23,0 | 24,1 | 21,0 |
| 11:20 | 23,4 | 21,2 | 51,2 | 57,0 | 26,8 | 21,6 | 0,7 | 0,2 | | 135,0 | | 135,0 | 37,4 | 22,2 | 27,6 | 21,2 |
| 11:25 | 23,6 | 21,4 | 51,3 | 56,9 | 26,7 | 22,2 | 1,5 | 0,2 | | 0,0 | | 90,0 | 42,3 | 23,3 | 27,5 | 21,8 |
| 11:30 | 24,2 | 21,6 | 50,0 | 56,4 | 27,9 | 22,0 | 0,3 | 0,3 | | 315,0 | | 90,0 | 34,6 | 23,0 | 28,6 | 21,3 |
| 11:35 | 24,5 | 21,6 | 48,7 | 56,8 | 26,3 | 22,0 | 1,3 | 0,1 | | 0,0 | | 90,0 | 34,6 | 22,4 | 25,7 | 22,2 |
| 11:40 | 23,8 | 21,6 | 50,4 | 56,1 | 25,5 | 22,2 | 0,5 | 0,0 | | 135,0 | | 90,0 | 29,8 | 22,6 | 25,1 | 23,5 |
| 11:45 | 24,1 | 21,7 | 49,2 | 55,2 | 28,2 | 22,2 | 2,3 | 0,2 | | 0,0 | | 315,0 | 52,3 | 23,3 | 30,3 | 22,0 |
| 11:50 | 23,7 | 21,8 | 49,7 | 55,2 | 23,4 | 22,2 | 1,7 | 0,0 | | 135,0 | | 90,0 | 21,4 | 22,4 | 19,6 | 23,4 |
| 11:55 | 23,5 | 21,8 | 50,9 | 54,9 | 26,3 | 22,4 | 1,7 | 0,1 | | 0,0 | | 90,0 | 41,2 | 22,9 | 26,5 | 22,5 |
| 12:00 | 24,1 | 21,8 | 48,2 | 53,9 | 26,8 | 22,3 | 0,2 | 0,2 | | 90,0 | | 90,0 | 31,2 | 23,0 | 27,3 | 21,9 |
| 12:05 | 24,7 | 22,0 | 46,3 | 53,3 | 27,0 | 22,8 | 0,6 | 0,3 | | 45,0 | | 315,0 | 34,1 | 24,4 | 27,4 | 22,2 |
| 12:10 | 24,3 | 22,1 | 47,1 | 53,4 | 27,1 | 22,9 | 1,6 | 0,1 | | 315,0 | | 90,0 | 41,3 | 23,7 | 27,5 | 23,0 |
| 12:15 | 24,9 | 22,3 | 45,7 | 52,0 | 27,8 | 23,0 | 0,3 | 0,6 | | 45,0 | | 90,0 | 33,0 | 25,2 | 28,1 | 21,6 |
| 12:20 | 24,9 | 22,3 | 45,9 | 52,8 | 27,5 | 23,0 | 0,3 | 0,1 | | 90,0 | | 90,0 | 32,5 | 23,7 | 27,9 | 23,1 |
| 12:25 | 25,3 | 22,3 | 45,4 | 52,4 | 28,0 | 23,0 | 2,4 | 0,7 | | 0,0 | | 90,0 | 45,7 | 25,4 | 28,7 | 21,4 |
| 12:30 | 24,9 | 22,4 | 45,6 | 51,8 | 27,1 | 23,0 | 1,1 | 0,2 | | 135,0 | | 270,0 | 36,5 | 24,0 | 27,2 | 22,7 |
| 12:35 | 24,8 | 22,4 | 45,2 | 50,9 | 25,5 | 22,8 | 0,6 | 0,1 | | 135,0 | | 90,0 | 27,7 | 23,2 | 24,6 | 22,9 |
| 12:40 | 24,3 | 22,4 | 46,0 | 51,9 | 24,5 | 22,9 | 2,4 | 0,2 | | 0,0 | | 90,0 | 26,2 | 23,8 | 20,8 | 22,6 |
| 12:45 | 24,3 | 22,5 | 45,5 | 51,2 | 25,2 | 23,3 | 2,1 | 0,2 | | 135,0 | | 135,0 | 31,0 | 24,5 | 22,8 | 23,0 |
| 12:50 | 24,8 | 22,5 | 44,5 | 50,5 | 27,9 | 23,0 | 2,9 | 0,0 | | 0,0 | | 270,0 | 49,2 | 23,3 | 28,7 | 24,1 |
| 12:55 | 25,1 | 22,6 | 43,8 | 49,9 | 27,3 | 23,0 | 2,0 | 0,1 | | 315,0 | | 135,0 | 40,0 | 23,4 | 27,0 | 23,1 |
| 13:00 | 25,4 | 22,6 | 44,0 | 50,1 | 28,4 | 23,1 | 1,8 | 0,2 | | 0,0 | | 45,0 | 44,6 | 23,8 | 29,5 | 22,7 |
| 13:05 | 25,8 | 22,6 | 42,8 | 50,2 | 29,2 | 23,1 | 2,3 | 0,6 | | 0,0 | | 90,0 | 49,5 | 24,6 | 30,9 | 21,6 |
| 13:10 | 25,0 | 22,6 | 44,5 | 49,8 | 24,5 | 22,8 | 1,6 | 0,2 | | 90,0 | | 315,0 | 21,8 | 23,0 | 20,9 | 22,3 |
| 13:15 | 24,0 | 22,5 | 46,8 | 50,2 | 26,4 | 22,9 | 0,9 | 0,3 | | 180,0 | | 90,0 | 35,1 | 23,7 | 26,4 | 22,2 |
| 13:20 | 24,8 | 22,6 | 45,1 | 50,6 | 29,1 | 23,3 | 1,4 | 0,1 | | 45,0 | | 315,0 | 48,3 | 24,3 | 31,4 | 23,6 |
| 13:25 | 25,6 | 22,8 | 43,5 | 50,1 | 28,1 | 23,4 | 2,0 | 0,5 | | 135,0 | | 90,0 | 42,7 | 25,4 | 28,5 | 22,4 |
| 13:30 | 24,5 | 22,9 | 46,2 | 50,0 | 24,5 | 23,4 | 2,3 | 0,1 | | 180,0 | | 45,0 | 24,5 | 24,0 | 20,6 | 23,6 |
| 13:35 | 24,9 | 23,0 | 45,2 | 50,2 | 28,4 | 24,4 | 0,7 | 0,1 | | 135,0 | | 45,0 | 39,0 | 26,0 | 29,4 | 24,7 |
| 13:40 | 25,1 | 23,3 | 45,1 | 49,3 | 26,7 | 24,6 | 0,9 | 0,0 | | 45,0 | | 315,0 | 32,5 | 25,4 | 26,1 | 25,6 |
| 13:45 | 24,9 | 23,4 | 45,5 | 48,8 | 26,2 | 24,1 | 2,3 | 0,1 | | 180,0 | | 315,0 | 34,7 | 24,9 | 24,4 | 24,3 |
| 13:50 | 24,5 | 23,4 | 46,5 | 48,5 | 25,0 | 24,0 | 0,6 | 0,2 | | 0,0 | | 90,0 | 26,7 | 25,0 | 23,9 | 23,8 |
| 13:55 | 24,3 | 23,3 | 46,5 | 49,8 | 25,4 | 23,7 | 0,2 | 0,1 | | 315,0 | | 45,0 | 27,2 | 24,2 | 25,4 | 23,9 |
| 14:00 | 24,7 | 23,3 | 46,3 | 50,2 | 25,8 | 24,2 | 0,1 | 0,1 | | 135,0 | | 135,0 | 27,1 | 24,8 | 26,1 | 24,2 |
| 14:05 | 24,7 | 23,4 | 46,7 | 50,4 | 25,2 | 24,3 | 1,3 | 0,1 | | 135,0 | | 90,0 | 28,1 | 25,3 | 23,3 | 24,5 |
| 14:10 | 24,4 | 23,5 | 47,2 | 49,5 | 25,4 | 24,3 | 0,7 | 0,5 | | 45,0 | | 90,0 | 28,6 | 26,8 | 24,4 | 23,5 |
| 14:15 | 24,6 | 23,4 | 47,4 | 50,2 | 25,8 | 24,0 | 0,3 | 0,1 | | 45,0 | | 90,0 | 28,2 | 24,5 | 25,6 | 24,1 |
| 14:20 | 24,5 | 23,5 | 47,1 | 49,8 | 25,4 | 24,3 | 1,4 | 0,3 | | 135,0 | | 135,0 | 30,0 | 25,7 | 23,6 | 23,7 |
| 14:25 | 24,5 | 23,4 | 47,7 | 50,2 | 25,1 | 23,9 | 0,6 | 0,1 | | 0,0 | | 0,0 | 27,0 | 24,4 | 24,0 | 24,0 |
| 14:30 | 24,2 | 23,4 | 48,5 | 50,0 | 24,9 | 23,5 | 1,1 | 0,1 | | 135,0 | | 135,0 | 27,5 | 23,6 | 23,0 | 23,6 |
| 14:35 | 23,9 | 23,2 | 49,5 | 50,5 | 24,2 | 23,3 | 0,8 | 0,0 | | 180,0 | | 135,0 | 25,4 | 23,3 | 22,4 | 24,4 |
| 14:40 | 23,8 | 23,1 | 49,7 | 51,4 | 24,4 | 23,5 | 0,4 | 0,1 | | 0,0 | | 90,0 | 25,7 | 23,8 | 23,6 | 23,6 |
| 14:45 | 23,8 | 23,0 | 49,8 | 50,8 | 24,4 | 23,4 | 0,4 | 0,3 | | 135,0 | | 135,0 | 25,8 | 24,3 | 23,6 | 22,8 |
| 14:50 | 23,6 | 23,0 | 50,8 | 51,8 | 24,0 | 23,2 | 0,9 | 0,6 | | 135,0 | | 90,0 | 25,8 | 23,7 | 22,2 | 21,5 |
| 14:55 | 23,8 | 22,9 | 50,1 | 52,2 | 24,5 | 23,3 | 1,3 | 0,3 | | 180,0 | | 135,0 | 28,1 | 23,9 | 22,5 | 22,5 |
| 15:00 | 23,7 | 22,9 | 50,4 | 52,5 | 24,1 | 23,2 | 0,9 | 0,4 | | 180,0 | | 135,0 | 25,6 | 23,9 | 22,2 | 22,2 |
| 15:05 | 23,7 | 22,9 | 50,7 | 52,8 | 24,2 | 23,2 | 0,5 | 0,1 | | 90,0 | | 90,0 | 25,8 | 23,4 | 23,3 | 23,3 |
| 15:10 | 23,8 | 22,9 | 50,6 | 52,8 | 24,3 | 23,4 | 0,4 | 0,0 | | 45,0 | | 90,0 | 25,7 | 23,7 | 23,6 | 24,6 |
| 15:15 | 23,8 | 22,9 | 50,3 | 52,4 | 24,3 | 23,2 | 0,6 | 0,4 | | 135,0 | | 135,0 | 25,8 | 24,0 | 23,0 | 22,2 |
| 15:20 | 23,8 | 22,9 | 50,7 | 53,0 | 24,2 | 23,4 | 0,5 | 0,5 | | 135,0 | | 135,0 | 25,4 | 24,7 | 23,1 | 22,2 |
| 15:25 | 23,8 | 22,9 | 50,2 | 52,5 | 24,1 | 23,3 | 0,8 | 0,3 | | 135,0 | | 90,0 | 25,3 | 24,2 | 22,3 | 22,7 |
| 15:30 | 23,6 | 23,0 | 50,8 | 52,3 | 24,2 | 23,5 | 1,5 | 0,0 | | 45,0 | | 90,0 | 27,5 | 23,7 | 21,7 | 24,6 |
| 15:35 | 23,7 | 23,0 | 50,2 | 52,2 | 24,5 | 23,6 | 0,4 | 0,0 | | 45,0 | | 90,0 | 26,4 | 23,8 | 23,8 | 24,6 |
| 15:40 | 23,8 | 23,0 | 50,4 | 52,8 | 24,2 | 23,5 | 1,2 | 0,2 | | 135,0 | | 135,0 | 26,0 | 24,4 | 21,9 | 23,3 |
| 15:45 | 23,7 | 23,0 | 51,0 | 52,9 | 24,2 | 23,4 | 0,2 | 0,2 | | 0,0 | | 135,0 | 24,8 | 24,0 | 23,9 | 23,1 |
| 15:50 | 23,6 | 22,9 | 51,5 | 53,5 | 24,1 | 23,2 | 0,4 | 0,1 | | 90,0 | | 45,0 | 25,2 | 23,4 | 23,2 | 23,3 |
| 15:55 | 23,6 | 22,9 | 52,1 | 53,8 | 23,9 | 23,1 | 2,2 | 0,0 | | 90,0 | | 90,0 | 25,8 | 23,2 | 20,3 | 24,3 |
| 16:00 | 23,3 | 22,8 | 52,8 | 54,4 | 23,7 | 23,0 | 0,7 | 0,1 | | 180,0 | | 135,0 | 25,1 | 23,2 | 22,1 | 23,2 |
| 16:05 | 22,8 | 22,8 | 54,3 | 54,7 | 22,9 | 22,9 | 3,4 | 0,1 | | 180,0 | | 135,0 | 23,5 | 23,1 | 18,4 | 23,1 |
| 16:10 | 22,6 | 22,7 | 54,5 | 54,5 | 22,9 | 22,8 | 2,3 | 0,2 | | 180,0 | | 135,0 | 25,5 | 23,0 | 19,3 | 22,5 |
| 16:15 | 22,4 | 22,5 | 54,5 | 54,2 | 22,8 | 22,6 | 1,5 | 0,0 | | 180,0 | | 90,0 | 25,0 | 22,7 | 19,8 | 23,8 |
| 16:20 | 22,2 | 22,5 | 55,3 | 54,8 | 22,7 | 22,7 | 0,9 | 0,1 | | 180,0 | | 135,0 | 24,5 | 23,0 | 20,5 | 22,9 |
| 16:25 | 22,4 | 22,4 | 54,9 | 55,1 | 23,1 | 22,6 | 1,2 | 0,4 | | 135,0 | | 135,0 | 26,3 | 22,9 | 20,7 | 21,4 |
| 16:30 | 22,1 | 22,3 | 55,7 | 55,3 | 22,3 | 22,3 | 1,5 | 0,9 | | 135,0 | | 135,0 | 23,5 | 22,3 | 19,1 | 19,8 |
| 16:35 | 21,9 | 22,2 | 56,2 | 55,9 | 22,2 | 22,2 | 1,1 | 0,2 | | 180,0 | | 135,0 | 23,8 | 22,3 | 19,6 | 21,8 |
| 16:40 | 22,0 | 22,1 | 56,0 | 55,8 | 22,3 | 22,0 | 0,3 | 0,0 | | 180,0 | | 135,0 | 23,1 | 21,9 | 21,6 | 23,3 |
| 16:45 | 22,1 | 21,9 | 55,8 | 56,3 | 22,3 | 21,9 | 0,3 | 0,2 | | 0,0 | | 135,0 | 22,9 | 21,9 | 21,6 | 21,5 |
| 16:50 | 21,9 | 21,8 | 56,4 | 56,7 | 22,2 | 21,8 | 0,4 | 0,2 | | 315,0 | | 90,0 | 22,8 | 21,8 | 21,0 | 21,3 |
| 16:55 | 21,8 | 21,7 | 56,7 | 56,7 | 22,1 | 21,7 | 1,8 | 0,2 | | 135,0 | | 135,0 | 24,2 | 21,6 | 18,8 | 21,2 |
| 17:00 | 21,6 | 21,7 | 57,2 | 57,1 | 21,8 | 21,7 | 1,2 | 0,1 | | 135,0 | | 90,0 | 22,9 | 21,8 | 18,9 | 21,9 |
