# Supplementary material for: Dataset on daytime outdoor thermal comfort for Belo Horizonte, Brazil
Source: Data Brief. 2016 Sep 20;9:530–5. doi: 10.1016/j.dib.2016.09.019 (PMC5054238; doi:10.1016/j.dib.2016.09.019)
Supplement: Supplementary file 3 — Supplementary material [file mmc3.docx]

| **Liberdade Square**  **11th March 2013** | | | | | | | | | | | |
| --- | --- | --- | --- | --- | --- | --- | --- | --- | --- | --- | --- |
| **Time** | **PET (°C)** | **Perception of Thermal**  **Sensation** | **Thermal**  **Comfort**  **Evaluation** | **Preference**  **of Thermal**  **Sensation** | **Height** | **Weight** | **Age** | **Sex** | **thermal insulation of clothing** | **Physical Activity** | **Location of respondent (sun/shadow)** |
| 07:15 | 25,9 | 5 | 1 | 4 | 2 | 1 | 1 | 2 | 2 | 1 | 1 |
| 07:20 | 25,8 | 5 | 1 | 3 | 3 | 2 | 1 | 1 | 2 | 2 | 1 |
| 07:24 | 25,8 | 5 | 1 | 3 | 3 | 4 | 3 | 1 | 2 | 2 | 1 |
| 07:39 | 24,3 | 5 | 1 | 3 | 3 | 1 | 1 | 2 | 2 | 1 | 2 |
| 07:33 | 23,7 | 4 | 1 | 4 | 2 | 1 | 1 | 2 | 2 | 2 | 2 |
| 07:37 | 23,1 | 7 | 3 | 1 | 4 | 2 | 4 | 1 | 2 | 2 | 1 |
| 07:40 | 25,2 | 5 | 1 | 4 | 4 | 4 | 5 | 1 | 1 | 2 | 1 |
| 07:48 | 25,1 | 4 | 1 | 4 | 2 | 2 | 2 | 2 | 2 | 1 | 1 |
| 07:52 | 23,5 | 6 | 2 | 4 | 4 | 2 | 4 | 1 | 2 | 2 | 2 |
| 08:00 | 24,0 | 4 | 1 | 4 | 4 | 4 | 2 | 1 | 2 | 2 | 2 |
| 07:10 | 25,5 | 4 | 1 | 4 | 4 | 5 | 4 | 1 | 3 | 2 | 2 |
| 07:15 | 25,3 | 3 | 1 | 4 | 4 | 4 | 3 | 1 | 1 | 2 | 2 |
| 07:17 | 25,3 | 4 | 1 | 4 | 4 | 5 | 3 | 1 | 2 | 2 | 2 |
| 07:20 | 25,4 | 4 | 1 | 1 | 3 | 4 | 1 | 1 | 2 | 2 | 2 |
| 07:24 | 25,4 | 6 | 3 | 1 | 3 | 4 | 2 | 2 | 2 | 2 | 2 |
| 07:30 | 23,4 | 4 | 1 | 4 | 2 | 3 | 1 | 2 | 2 | 2 | 1 |
| 07:35 | 23,1 | 4 | 3 | 1 | 3 | 4 | 2 | 2 | 1 | 2 | 1 |
| 07:40 | 25,2 | 5 | 1 | 4 | 3 | 2 | 1 | 2 | 2 | 2 | 1 |
| 07:45 | 25,1 | 5 | 3 | 2 | 2 | 3 | 3 | 2 | 2 | 2 | 1 |
| 07:50 | 23,5 | 4 | 1 | 5 | 5 | 4 | 7 | 1 | 1 | 99 | 2 |
| 08:05 | 24,0 | 4 | 1 | 2 | 4 | 1 | 4 | 1 | 2 | 1 | 2 |
| 08:08 | 24,9 | 4 | 1 | 4 | 2 | 1 | 4 | 2 | 2 | 3 | 1 |
| 08:12 | 23,8 | 4 | 1 | 4 | 4 | 4 | 5 | 1 | 1 | 2 | 1 |
| 08:15 | 23,9 | 6 | 1 | 4 | 4 | 1 | 4 | 1 | 1 | 3 | 1 |
| 08:30 | 29,3 | 6 | 1 | 3 | 4 | 1 | 5 | 1 | 1 | 1 | 1 |
| 08:34 | 29,3 | 7 | 3 | 3 | 4 | 5 | 2 | 1 | 4 | 2 | 1 |
| 08:37 | 36,2 | 7 | 1 | 1 | 3 | 3 | 1 | 2 | 99 | 2 | 1 |
| 08:40 | 32,7 | 4 | 1 | 3 | 4 | 1 | 5 | 1 | 3 | 2 | 1 |
| 08:50 | 37,2 | 4 | 1 | 4 | 3 | 2 | 3 | 2 | 2 | 2 | 1 |
| 08:55 | 25,2 | 7 | 3 | 3 | 4 | 2 | 3 | 2 | 2 | 1 | 2 |
| 08:03 | 24,5 | 4 | 1 | 2 | 3 | 4 | 3 | 2 | 1 | 2 | 1 |
| 08:10 | 23,8 | 6 | 3 | 2 | 5 | 4 | 1 | 1 | 1 | 2 | 1 |
| 08:14 | 23,8 | 4 | 2 | 1 | 3 | 4 | 2 | 2 | 2 | 1 | 1 |
| 08:18 | 23,9 | 5 | 3 | 1 | 4 | 4 | 1 | 1 | 3 | 1 | 1 |
| 08:22 | 25,5 | 4 | 1 | 3 | 4 | 5 | 2 | 1 | 3 | 1 | 1 |
| 08:26 | 25,8 | 5 | 1 | 2 | 3 | 3 | 1 | 2 | 1 | 2 | 1 |
| 08:30 | 29,3 | 5 | 1 | 3 | 2 | 4 | 5 | 2 | 3 | 1 | 1 |
| 08:34 | 29,3 | 6 | 3 | 2 | 2 | 3 | 3 | 1 | 3 | 2 | 1 |
| 08:38 | 24,5 | 4 | 1 | 1 | 2 | 2 | 2 | 2 | 3 | 2 | 2 |
| 08:47 | 34,8 | 6 | 3 | 2 | 2 | 1 | 3 | 2 | 3 | 1 | 1 |
| 09:05 | 29,4 | 4 | 1 | 3 | 4 | 2 | 99 | 1 | 1 | 3 | 1 |
| 09:08 | 26,3 | 4 | 1 | 3 | 3 | 2 | 1 | 2 | 2 | 1 | 2 |
| 09:12 | 29,6 | 5 | 2 | 1 | 3 | 3 | 2 | 2 | 2 | 1 | 1 |
| 09:15 | 27,3 | 4 | 1 | 4 | 2 | 1 | 3 | 2 | 1 | 1 | 2 |
| 09:20 | 29,4 | 4 | 1 | 4 | 4 | 1 | 3 | 2 | 2 | 3 | 1 |
| 09:25 | 27,1 | 4 | 1 | 4 | 2 | 1 | 2 | 2 | 2 | 1 | 2 |
| 09:30 | 27,4 | 6 | 2 | 4 | 3 | 3 | 1 | 1 | 2 | 1 | 2 |
| 09:34 | 30,1 | 6 | 1 | 4 | 3 | 3 | 1 | 2 | 2 | 3 | 1 |
| 09:38 | 27,1 | 5 | 1 | 4 | 3 | 3 | 3 | 2 | 1 | 3 | 2 |
| 09:57 | 28,2 | 5 | 1 | 4 | 4 | 4 | 1 | 1 | 2 | 1 | 2 |
| 09:10 | 29,6 | 4 | 1 | 3 | 3 | 3 | 1 | 1 | 1 | 1 | 1 |
| 09:15 | 31,7 | 7 | 3 | 2 | 2 | 2 | 1 | 2 | 3 | 1 | 1 |
| 09:21 | 29,4 | 7 | 3 | 2 | 4 | 4 | 2 | 1 | 3 | 2 | 1 |
| 09:27 | 27,1 | 5 | 1 | 3 | 2 | 3 | 1 | 2 | 2 | 1 | 2 |
| 09:30 | 30,1 | 6 | 1 | 2 | 4 | 5 | 1 | 1 | 2 | 2 | 1 |
| 09:36 | 29,3 | 5 | 2 | 3 | 3 | 3 | 2 | 2 | 3 | 2 | 1 |
| 09:41 | 32,0 | 4 | 2 | 2 | 4 | 5 | 3 | 1 | 3 | 2 | 1 |
| 09:46 | 33,3 | 4 | 1 | 3 | 3 | 4 | 4 | 1 | 3 | 1 | 1 |
| 09:52 | 39,3 | 7 | 3 | 1 | 4 | 4 | 4 | 1 | 3 | 1 | 1 |
| 09:58 | 35,3 | 5 | 1 | 2 | 3 | 5 | 3 | 1 | 3 | 2 | 1 |
| 10:06 | 33,5 | 5 | 2 | 3 | 4 | 2 | 4 | 1 | 1 | 3 | 1 |
| 10:11 | 27,7 | 4 | 1 | 1 | 3 | 3 | 1 | 1 | 2 | 1 | 2 |
| 10:15 | 31,8 | 7 | 2 | 3 | 3 | 2 | 2 | 2 | 1 | 3 | 1 |
| 10:19 | 28,6 | 7 | 3 | 2 | 4 | 1 | 2 | 2 | 2 | 1 | 2 |
| 10:23 | 31,7 | 7 | 3 | 1 | 4 | 1 | 4 | 1 | 2 | 3 | 1 |
| 10:50 | 32,0 | 5 | 2 | 3 | 3 | 3 | 4 | 2 | 1 | 3 | 1 |
| 10:38 | 29,1 | 6 | 1 | 2 | 3 | 2 | 3 | 2 | 2 | 1 | 2 |
| 10:42 | 28,2 | 6 | 2 | 3 | 4 | 3 | 1 | 2 | 1 | 3 | 2 |
| 10:50 | 28,9 | 7 | 4 | 1 | 4 | 5 | 3 | 1 | 2 | 3 | 2 |
| 10:59 | 31,9 | 4 | 1 | 3 | 4 | 4 | 2 | 1 | 2 | 3 | 1 |
| 10:15 | 31,8 | 5 | 2 | 2 | 2 | 3 | 1 | 2 | 3 | 2 | 1 |
| 10:22 | 28,4 | 5 | 1 | 2 | 4 | 4 | 3 | 1 | 3 | 1 | 2 |
| 10:26 | 32,5 | 6 | 3 | 2 | 2 | 3 | 4 | 2 | 3 | 2 | 1 |
| 10:30 | 28,6 | 7 | 3 | 1 | 4 | 5 | 2 | 1 | 3 | 1 | 2 |
| 10:35 | 29,1 | 4 | 1 | 2 | 2 | 2 | 1 | 2 | 2 | 2 | 2 |
| 10:41 | 28,2 | 5 | 2 | 3 | 3 | 4 | 2 | 2 | 3 | 1 | 2 |
| 10:45 | 28,4 | 5 | 2 | 3 | 2 | 3 | 1 | 1 | 3 | 1 | 2 |
| 10:51 | 28,9 | 5 | 3 | 2 | 4 | 4 | 1 | 1 | 3 | 2 | 2 |
| 10:55 | 31,9 | 7 | 3 | 1 | 3 | 3 | 2 | 1 | 3 | 2 | 1 |
| 11:00 | 29,1 | 7 | 4 | 1 | 3 | 4 | 4 | 2 | 1 | 1 | 2 |
| 11:10 | 32,2 | 7 | 3 | 3 | 4 | 1 | 4 | 2 | 2 | 3 | 1 |
| 11:17 | 32,7 | 4 | 2 | 3 | 4 | 1 | 3 | 1 | 2 | 3 | 1 |
| 11:23 | 28,8 | 6 | 2 | 2 | 3 | 5 | 2 | 2 | 2 | 1 | 2 |
| 11:29 | 33,8 | 6 | 3 | 3 | 2 | 1 | 1 | 2 | 2 | 3 | 1 |
| 11:34 | 33,1 | 7 | 3 | 1 | 2 | 2 | 3 | 2 | 1 | 3 | 1 |
| 11:40 | 29,7 | 4 | 2 | 4 | 2 | 2 | 2 | 2 | 2 | 1 | 2 |
| 11:44 | 29,7 | 7 | 3 | 3 | 2 | 3 | 3 | 2 | 2 | 1 | 2 |
| 11:47 | 29,0 | 7 | 3 | 2 | 4 | 2 | 2 | 2 | 2 | 1 | 2 |
| 11:50 | 29,7 | 7 | 2 | 3 | 3 | 2 | 2 | 2 | 2 | 1 | 2 |
| 12:00 | 29,7 | 7 | 2 | 2 | 2 | 4 | 2 | 2 | 2 | 1 | 2 |
| 11:10 | 29,8 | 7 | 4 | 1 | 4 | 4 | 2 | 1 | 2 | 2 | 2 |
| 11:15 | 28,8 | 5 | 2 | 2 | 3 | 2 | 1 | 2 | 2 | 1 | 2 |
| 11:19 | 32,7 | 4 | 1 | 2 | 4 | 5 | 2 | 2 | 2 | 2 | 1 |
| 11:23 | 33,7 | 4 | 1 | 4 | 4 | 4 | 2 | 1 | 3 | 2 | 1 |
| 11:27 | 28,9 | 7 | 4 | 2 | 3 | 3 | 2 | 2 | 3 | 1 | 2 |
| 11:32 | 29,2 | 7 | 2 | 1 | 3 | 4 | 1 | 1 | 3 | 1 | 2 |
| 11:41 | 34,3 | 7 | 2 | 1 | 3 | 4 | 1 | 1 | 3 | 2 | 1 |
| 11:45 | 33,9 | 7 | 3 | 3 | 2 | 2 | 1 | 2 | 2 | 2 | 1 |
| 11:50 | 35,4 | 6 | 3 | 4 | 3 | 5 | 3 | 1 | 3 | 2 | 1 |
| 11:58 | 33,6 | 7 | 2 | 4 | 4 | 5 | 2 | 1 | 3 | 2 | 1 |
| 12:21 | 35,4 | 7 | 4 | 2 | 3 | 2 | 1 | 2 | 1 | 3 | 1 |
| 12:29 | 29,1 | 7 | 3 | 3 | 4 | 4 | 3 | 1 | 2 | 3 | 3 |
| 12:33 | 29,1 | 7 | 3 | 2 | 3 | 4 | 1 | 2 | 2 | 3 | 3 |
| 12:38 | 35,8 | 5 | 1 | 3 | 3 | 2 | 2 | 2 | 2 | 3 | 1 |
| 12:42 | 34,0 | 7 | 3 | 4 | 3 | 4 | 3 | 2 | 2 | 3 | 1 |
| 12:51 | 29,2 | 4 | 1 | 3 | 3 | 3 | 1 | 2 | 2 | 1 | 3 |
| 12:58 | 29,2 | 5 | 1 | 3 | 3 | 1 | 3 | 2 | 2 | 1 | 3 |
| 13:01 | 38,6 | 7 | 4 | 2 | 3 | 2 | 1 | 2 | 2 | 3 | 1 |
| 13:12 | 29,8 | 7 | 4 | 3 | 3 | 4 | 2 | 2 | 2 | 1 | 2 |
| 13:20 | 30,2 | 5 | 1 | 4 | 4 | 4 | 4 | 1 | 2 | 1 | 2 |
| 13:27 | 30,7 | 7 | 2 | 3 | 3 | 3 | 3 | 2 | 1 | 3 | 2 |
| 13:31 | 30,6 | 7 | 2 | 3 | 3 | 2 | 1 | 2 | 3 | 1 | 2 |
| 13:35 | 30,4 | 7 | 3 | 3 | 4 | 4 | 3 | 1 | 3 | 3 | 2 |
| 12:00 | 29,7 | 4 | 2 | 5 | 4 | 4 | 4 | 1 | 2 | 1 | 2 |
| 12:05 | 29,9 | 4 | 2 | 3 | 3 | 3 | 2 | 1 | 2 | 3 | 2 |
| 12:15 | 29,4 | 7 | 3 | 3 | 4 | 5 | 2 | 2 | 2 | 3 | 2 |
| 12:25 | 29,1 | 7 | 3 | 1 | 3 | 3 | 1 | 2 | 1 | 1 | 2 |
| 12:45 | 29,2 | 7 | 1 | 2 | 4 | 3 | 1 | 1 | 2 | 1 | 2 |
| 12:49 | 29,2 | 6 | 1 | 1 | 4 | 2 | 1 | 2 | 3 | 3 | 2 |
| 12:53 | 29,2 | 4 | 1 | 4 | 4 | 4 | 2 | 2 | 3 | 3 | 2 |
| 12:57 | 29,2 | 7 | 3 | 1 | 3 | 3 | 1 | 1 | 3 | 1 | 2 |
| 13:01 | 29,3 | 7 | 3 | 1 | 4 | 4 | 4 | 1 | 3 | 3 | 2 |
| 13:04 | 29,3 | 4 | 1 | 4 | 2 | 2 | 1 | 1 | 3 | 1 | 2 |
| 13:10 | 29,8 | 7 | 4 | 2 | 2 | 4 | 2 | 2 | 3 | 3 | 2 |
| 13:15 | 30,1 | 7 | 4 | 2 | 2 | 4 | 4 | 2 | 3 | 3 | 2 |
| 13:20 | 30,2 | 7 | 4 | 1 | 4 | 4 | 2 | 1 | 4 | 1 | 2 |
| 13:30 | 30,6 | 5 | 2 | 1 | 4 | 4 | 1 | 1 | 1 | 4 | 2 |
| 13:35 | 41,4 | 4 | 1 | 3 | 4 | 5 | 2 | 1 | 3 | 1 | 1 |
| 13:40 | 30,2 | 6 | 1 | 4 | 4 | 2 | 2 | 1 | 3 | 1 | 2 |
| 13:45 | 30,5 | 4 | 1 | 3 | 4 | 3 | 1 | 1 | 1 | 1 | 2 |
| 13:49 | 30,5 | 6 | 1 | 3 | 3 | 4 | 1 | 2 | 2 | 1 | 2 |
| 13:55 | 30,7 | 7 | 3 | 2 | 4 | 4 | 1 | 1 | 3 | 3 | 2 |
| 14:00 | 30,7 | 7 | 4 | 2 | 4 | 2 | 1 | 1 | 3 | 3 | 2 |
| 13:43 | 30,2 | 7 | 2 | 3 | 3 | 3 | 3 | 2 | 2 | 1 | 2 |
| 13:47 | 30,5 | 7 | 2 | 3 | 3 | 3 | 3 | 2 | 2 | 3 | 2 |
| 13:52 | 30,8 | 7 | 3 | 4 | 3 | 3 | 2 | 2 | 1 | 3 | 2 |
| 14:01 | 30,7 | 7 | 4 | 2 | 3 | 4 | 1 | 2 | 2 | 1 | 2 |
| 14:06 | 30,3 | 7 | 4 | 3 | 3 | 3 | 2 | 2 | 2 | 1 | 2 |
| 14:10 | 31,0 | 7 | 3 | 3 | 3 | 3 | 3 | 2 | 2 | 3 | 2 |
| 14:25 | 41,4 | 7 | 2 | 3 | 4 | 4 | 1 | 1 | 2 | 3 | 1 |
| 14:31 | 31,1 | 7 | 1 | 4 | 3 | 4 | 1 | 2 | 2 | 1 | 2 |
| 14:20 | 30,6 | 7 | 3 | 3 | 3 | 3 | 2 | 2 | 2 | 1 | 2 |
| 14:25 | 30,3 | 7 | 1 | 2 | 4 | 5 | 2 | 1 | 3 | 1 | 2 |
| 14:30 | 31,1 | 7 | 4 | 2 | 3 | 4 | 1 | 1 | 1 | 1 | 2 |
| 14:35 | 31,1 | 7 | 4 | 2 | 3 | 4 | 1 | 2 | 2 | 1 | 2 |
| 14:40 | 30,4 | 4 | 2 | 2 | 4 | 5 | 2 | 1 | 1 | 1 | 2 |
| 14:45 | 30,6 | 7 | 2 | 2 | 3 | 3 | 4 | 1 | 3 | 3 | 2 |
| 14:50 | 30,6 | 7 | 3 | 2 | 4 | 4 | 1 | 1 | 2 | 1 | 2 |
| 14:55 | 39,1 | 7 | 4 | 1 | 4 | 5 | 3 | 1 | 3 | 3 | 1 |
| 14:59 | 30,9 | 4 | 3 | 3 | 2 | 3 | 2 | 2 | 3 | 99 | 2 |
| 15:03 | 31,3 | 7 | 3 | 1 | 4 | 5 | 1 | 1 | 3 | 3 | 2 |
| 14:38 | 31,1 | 6 | 1 | 3 | 3 | 2 | 1 | 2 | 1 | 3 | 2 |
| 14:52 | 30,6 | 7 | 2 | 3 | 4 | 5 | 2 | 1 | 2 | 3 | 2 |
| 14:56 | 39,1 | 7 | 2 | 2 | 3 | 4 | 1 | 2 | 2 | 3 | 1 |
| 15:02 | 31,3 | 6 | 1 | 3 | 2 | 1 | 1 | 2 | 2 | 3 | 2 |
| 15:08 | 36,4 | 7 | 3 | 2 | 2 | 2 | 2 | 1 | 1 | 3 | 1 |
| 15:23 | 31,8 | 7 | 4 | 2 | 2 | 1 | 1 | 2 | 1 | 1 | 2 |
| 15:23 | 31,8 | 7 | 3 | 3 | 3 | 2 | 1 | 2 | 1 | 1 | 2 |
| 15:31 | 31,2 | 6 | 2 | 3 | 3 | 2 | 1 | 2 | 2 | 3 | 2 |
| 15:35 | 31,3 | 4 | 1 | 4 | 3 | 3 | 3 | 2 | 2 | 3 | 2 |
| 15:41 | 40,8 | 5 | 1 | 3 | 4 | 4 | 3 | 1 | 2 | 3 | 1 |
| 15:10 | 31,5 | 7 | 3 | 3 | 3 | 3 | 2 | 2 | 3 | 1 | 2 |
| 15:15 | 31,4 | 7 | 1 | 3 | 2 | 3 | 4 | 2 | 3 | 3 | 2 |
| 15:20 | 31,8 | 5 | 2 | 4 | 3 | 4 | 1 | 2 | 3 | 1 | 2 |
| 15:24 | 31,8 | 7 | 2 | 3 | 3 | 3 | 1 | 2 | 3 | 1 | 2 |
| 15:28 | 37,4 | 7 | 2 | 3 | 4 | 4 | 1 | 1 | 1 | 1 | 1 |
| 15:32 | 31,7 | 7 | 4 | 1 | 4 | 3 | 1 | 1 | 3 | 3 | 1 |
| 15:36 | 37,8 | 7 | 1 | 3 | 3 | 2 | 2 | 1 | 3 | 3 | 1 |
| 15:40 | 40,8 | 7 | 2 | 1 | 4 | 4 | 2 | 1 | 3 | 3 | 1 |
| 16:21 | 31,1 | 5 | 1 | 3 | 2 | 2 | 3 | 1 | 2 | 3 | 2 |
| 16:25 | 31,4 | 4 | 1 | 2 | 4 | 4 | 1 | 1 | 3 | 1 | 2 |
| 16:52 | 31,4 | 4 | 1 | 4 | 3 | 2 | 2 | 2 | 2 | 3 | 2 |
| 17:00 | 30,9 | 4 | 1 | 4 | 2 | 2 | 1 | 2 | 2 | 3 | 2 |
| 16:40 | 31,3 | 4 | 1 | 2 | 4 | 5 | 1 | 1 | 1 | 1 | 1 |
| 16:45 | 29,4 | 7 | 3 | 3 | 4 | 2 | 3 | 1 | 3 | 3 | 1 |
| 16:50 | 31,4 | 5 | 1 | 4 | 4 | 2 | 1 | 1 | 3 | 3 | 2 |
| 16:55 | 31,2 | 7 | 2 | 3 | 3 | 3 | 3 | 2 | 3 | 3 | 2 |
| 17:00 | 30,9 | 7 | 2 | 3 | 3 | 2 | 3 | 2 | 3 | 1 | 2 |
| 17:00 | 30,9 | 7 | 1 | 4 | 4 | 5 | 4 | 1 | 1 | 1 | 2 |
| 15:49 | 36,4 | 5 | 1 | 4 | 3 | 3 | 1 | 2 | 2 | 3 | 1 |
| 15:59 | 31,9 | 7 | 2 | 3 | 3 | 2 | 1 | 2 | 2 | 1 | 2 |
| 16:08 | 31,2 | 7 | 4 | 3 | 3 | 3 | 3 | 2 | 2 | 3 | 2 |
| 16:20 | 36,6 | 5 | 1 | 4 | 3 | 3 | 3 | 2 | 2 | 3 | 1 |
| 16:34 | 31,2 | 5 | 1 | 4 | 3 | 2 | 1 | 2 | 1 | 3 | 2 |
| 16:34 | 31,2 | 6 | 2 | 3 | 3 | 3 | 1 | 2 | 2 | 3 | 2 |
| 16:42 | 31,5 | 1 | 4 | 2 | 3 | 3 | 4 | 2 | 1 | 3 | 2 |
| 07:36 | 24,3 | 7 | 2 | 3 | 4 | 4 | 2 | 2 | 2 | 1 | 2 |
| 07:43 | 23,3 | 7 | 2 | 3 | 4 | 3 | 2 | 1 | 1 | 1 | 2 |
| 07:47 | 24,9 | 7 | 2 | 4 | 4 | 3 | 2 | 1 | 1 | 3 | 2 |
| 07:53 | 25,0 | 4 | 2 | 1 | 4 | 3 | 1 | 1 | 2 | 3 | 1 |
| 07:59 | 23,7 | 6 | 1 | 3 | 3 | 4 | 4 | 2 | 2 | 3 | 2 |
| 08:08 | 24,9 | 4 | 2 | 3 | 3 | 4 | 4 | 1 | 1 | 3 | 1 |
| 08:19 | 24,7 | 3 | 1 | 2 | 3 | 3 | 4 | 1 | 2 | 3 | 2 |
| 08:45 | 34,8 | 4 | 3 | 3 | 3 | 2 | 4 | 2 | 2 | 3 | 1 |
| 08:49 | 26,5 | 4 | 1 | 4 | 3 | 1 | 1 | 2 | 2 | 1 | 2 |
| 08:50 | 25,6 | 5 | 2 | 1 | 3 | 4 | 4 | 2 | 2 | 3 | 2 |
| 07:14 | 25,5 | 5 | 2 | 3 | 4 | 3 | 2 | 1 | 2 | 1 | 2 |
| 07:17 | 25,3 | 6 | 1 | 3 | 2 | 2 | 1 | 2 | 2 | 3 | 2 |
| 07:23 | 25,4 | 6 | 1 | 3 | 4 | 5 | 3 | 1 | 2 | 1 | 2 |
| 07:28 | 26,3 | 7 | 2 | 4 | 1 | 1 | 2 | 2 | 2 | 3 | 2 |
| 07:32 | 23,7 | 4 | 1 | 4 | 4 | 4 | 4 | 1 | 1 | 1 | 2 |
| 07:40 | 23,3 | 4 | 1 | 4 | 3 | 2 | 1 | 2 | 2 | 1 | 2 |
| 07:47 | 24,9 | 6 | 2 | 1 | 4 | 5 | 2 | 1 | 3 | 2 | 2 |
| 07:52 | 23,5 | 5 | 1 | 3 | 4 | 5 | 3 | 1 | 1 | 2 | 2 |
| 07:56 | 23,7 | 3 | 1 | 4 | 3 | 4 | 3 | 2 | 4 | 1 | 2 |
| 07:59 | 25,3 | 7 | 3 | 3 | 3 | 3 | 3 | 2 | 2 | 99 | 1 |
| 08:05 | 24,0 | 7 | 3 | 3 | 4 | 5 | 3 | 1 | 1 | 4 | 2 |
| 08:10 | 24,3 | 4 | 1 | 4 | 4 | 4 | 1 | 1 | 1 | 1 | 2 |
| 08:15 | 23,9 | 4 | 1 | 4 | 2 | 5 | 4 | 2 | 2 | 1 | 1 |
| 08:20 | 23,3 | 5 | 1 | 1 | 4 | 5 | 1 | 1 | 1 | 1 | 2 |
| 08:23 | 25,5 | 4 | 1 | 4 | 4 | 4 | 1 | 1 | 1 | 3 | 1 |
| 08:27 | 25,8 | 7 | 4 | 3 | 3 | 2 | 1 | 2 | 2 | 3 | 1 |
| 08:30 | 29,3 | 4 | 2 | 3 | 2 | 2 | 2 | 2 | 2 | 3 | 1 |
| 08:40 | 25,4 | 6 | 2 | 3 | 3 | 3 | 1 | 1 | 4 | 1 | 2 |
| 08:44 | 25,4 | 6 | 1 | 3 | 3 | 3 | 3 | 2 | 3 | 1 | 2 |
| 08:50 | 25,6 | 6 | 2 | 3 | 3 | 3 | 2 | 2 | 2 | 1 | 2 |
| 09:02 | 25,8 | 4 | 1 | 3 | 4 | 5 | 3 | 1 | 2 | 1 | 2 |
| 09:05 | 26,3 | 6 | 1 | 1 | 4 | 5 | 1 | 1 | 1 | 1 | 2 |
| 09:09 | 26,3 | 5 | 2 | 2 | 3 | 3 | 3 | 1 | 3 | 1 | 2 |
| 09:14 | 29,6 | 4 | 2 | 3 | 3 | 2 | 2 | 2 | 1 | 3 | 1 |
| 10:00 | 27,2 | 4 | 2 | 3 | 3 | 3 | 3 | 1 | 2 | 3 | 2 |
| 09:21 | 26,9 | 4 | 1 | 3 | 3 | 2 | 1 | 1 | 2 | 1 | 2 |
| 09:56 | 28,2 | 7 | 1 | 3 | 4 | 4 | 3 | 1 | 2 | 3 | 2 |
| 09:56 | 28,2 | 7 | 2 | 3 | 4 | 3 | 3 | 1 | 2 | 3 | 2 |
| 08:56 | 31,4 | 7 | 3 | 2 | 4 | 5 | 2 | 1 | 2 | 3 | 1 |
| 08:59 | 31,4 | 6 | 2 | 3 | 4 | 4 | 1 | 1 | 2 | 3 | 1 |
| 09:05 | 29,4 | 4 | 2 | 3 | 4 | 4 | 1 | 1 | 1 | 3 | 1 |
| 09:10 | 27,1 | 4 | 1 | 2 | 4 | 5 | 1 | 1 | 1 | 1 | 2 |
| 09:12 | 27,1 | 4 | 1 | 3 | 4 | 4 | 1 | 1 | 1 | 1 | 2 |
| 09:21 | 29,4 | 4 | 1 | 6 | 4 | 5 | 2 | 1 | 1 | 3 | 1 |
| 09:25 | 29,8 | 6 | 2 | 3 | 3 | 3 | 1 | 2 | 2 | 3 | 1 |
| 09:30 | 30,1 | 7 | 2 | 3 | 3 | 3 | 1 | 2 | 2 | 3 | 1 |
| 09:33 | 30,1 | 7 | 2 | 4 | 4 | 4 | 2 | 1 | 3 | 3 | 1 |
| 09:36 | 29,3 | 7 | 2 | 3 | 4 | 5 | 4 | 1 | 1 | 3 | 1 |
| 10:04 | 27,2 | 6 | 2 | 2 | 3 | 1 | 2 | 2 | 2 | 3 | 2 |
| 10:09 | 27,4 | 4 | 1 | 4 | 3 | 2 | 1 | 1 | 1 | 3 | 2 |
| 10:13 | 27,7 | 5 | 2 | 3 | 4 | 5 | 3 | 1 | 2 | 2 | 2 |
| 10:24 | 31,7 | 6 | 2 | 3 | 2 | 1 | 1 | 2 | 2 | 3 | 1 |
| 10:40 | 28,2 | 7 | 3 | 3 | 3 | 3 | 3 | 2 | 2 | 3 | 2 |
| 10:46 | 28,4 | 5 | 2 | 3 | 3 | 2 | 4 | 1 | 2 | 1 | 2 |
| 10:53 | 28,9 | 7 | 2 | 3 | 3 | 2 | 2 | 2 | 2 | 3 | 2 |
| 09:43 | 26,9 | 4 | 1 | 3 | 4 | 5 | 3 | 1 | 2 | 1 | 2 |
| 09:49 | 27,2 | 7 | 2 | 4 | 3 | 3 | 1 | 2 | 2 | 1 | 2 |
| 09:55 | 28,2 | 4 | 1 | 4 | 4 | 5 | 3 | 1 | 1 | 1 | 2 |
| 10:00 | 34,9 | 5 | 2 | 2 | 4 | 4 | 1 | 1 | 2 | 3 | 1 |
| 10:10 | 34,8 | 7 | 2 | 3 | 4 | 4 | 1 | 1 | 2 | 3 | 1 |
| 10:13 | 34,8 | 7 | 2 | 3 | 4 | 5 | 1 | 1 | 2 | 3 | 1 |
| 10:17 | 31,8 | 6 | 1 | 3 | 4 | 4 | 3 | 1 | 2 | 3 | 1 |
| 10:29 | 32,5 | 7 | 1 | 2 | 2 | 3 | 3 | 2 | 1 | 3 | 1 |
| 10:35 | 33,8 | 7 | 2 | 3 | 2 | 3 | 1 | 2 | 2 | 3 | 1 |
| 10:40 | 34,0 | 6 | 2 | 4 | 4 | 4 | 3 | 1 | 2 | 3 | 1 |
| 10:53 | 32,0 | 7 | 4 | 2 | 3 | 4 | 4 | 1 | 2 | 3 | 1 |
| 10:57 | 31,9 | 4 | 1 | 3 | 3 | 4 | 1 | 1 | 2 | 3 | 1 |
| 11:01 | 29,1 | 7 | 3 | 1 | 3 | 3 | 1 | 1 | 2 | 3 | 2 |
| 11:06 | 32,0 | 5 | 2 | 2 | 3 | 4 | 1 | 1 | 1 | 3 | 1 |
| 11:13 | 32,2 | 7 | 3 | 4 | 4 | 5 | 2 | 1 | 2 | 3 | 1 |
| 11:19 | 28,8 | 7 | 2 | 3 | 3 | 4 | 3 | 2 | 2 | 1 | 2 |
| 11:22 | 28,8 | 6 | 2 | 3 | 4 | 4 | 1 | 1 | 2 | 3 | 2 |
| 11:27 | 28,9 | 7 | 2 | 2 | 4 | 5 | 4 | 1 | 2 | 3 | 2 |
| 11:31 | 29,2 | 6 | 2 | 3 | 4 | 3 | 3 | 2 | 2 | 3 | 2 |
| 11:38 | 29,5 | 6 | 2 | 2 | 4 | 4 | 4 | 1 | 1 | 1 | 2 |
| 11:05 | 32,0 | 7 | 1 | 5 | 3 | 3 | 4 | 2 | 2 | 3 | 1 |
| 11:10 | 28,9 | 7 | 4 | 2 | 2 | 2 | 1 | 2 | 2 | 1 | 2 |
| 11:15 | 28,8 | 5 | 1 | 4 | 3 | 3 | 4 | 2 | 2 | 1 | 2 |
| 11:19 | 28,8 | 6 | 2 | 3 | 3 | 4 | 1 | 2 | 2 | 3 | 2 |
| 11:31 | 29,2 | 7 | 2 | 3 | 3 | 3 | 3 | 2 | 2 | 1 | 2 |
| 11:37 | 32,1 | 7 | 1 | 3 | 3 | 2 | 2 | 2 | 2 | 3 | 1 |
| 11:50 | 35,4 | 4 | 1 | 3 | 4 | 4 | 1 | 1 | 2 | 3 | 1 |
| 12:01 | 29,7 | 5 | 1 | 4 | 3 | 5 | 3 | 1 | 2 | 1 | 2 |
| 12:06 | 34,6 | 7 | 3 | 1 | 4 | 4 | 1 | 1 | 2 | 3 | 1 |
| 12:08 | 34,6 | 6 | 3 | 1 | 4 | 4 | 1 | 1 | 2 | 3 | 1 |
| 12:11 | 29,7 | 4 | 1 | 2 | 3 | 3 | 2 | 2 | 2 | 1 | 2 |
| 12:15 | 29,4 | 7 | 1 | 3 | 3 | 4 | 2 | 2 | 2 | 1 | 2 |
| 12:19 | 32,8 | 7 | 3 | 3 | 3 | 3 | 1 | 2 | 2 | 3 | 1 |
| 12:24 | 35,4 | 6 | 3 | 4 | 3 | 3 | 2 | 2 | 2 | 3 | 1 |
| 12:29 | 36,0 | 7 | 4 | 1 | 3 | 4 | 3 | 1 | 2 | 3 | 1 |
| 12:35 | 35,6 | 7 | 4 | 3 | 3 | 4 | 2 | 2 | 2 | 3 | 1 |
| 12:40 | 34,0 | 5 | 2 | 2 | 3 | 4 | 3 | 1 | 2 | 1 | 1 |
| 12:45 | 35,0 | 7 | 4 | 3 | 3 | 3 | 1 | 2 | 2 | 3 | 1 |
| 12:51 | 36,7 | 7 | 3 | 3 | 4 | 5 | 2 | 1 | 3 | 3 | 1 |
| 12:55 | 38,2 | 7 | 3 | 2 | 4 | 4 | 2 | 1 | 2 | 3 | 1 |
| 13:37 | 30,4 | 6 | 2 | 3 | 3 | 3 | 1 | 2 | 2 | 3 | 2 |
| 13:45 | 30,5 | 7 | 1 | 4 | 3 | 4 | 1 | 1 | 1 | 1 | 2 |
| 13:50 | 30,8 | 7 | 4 | 1 | 3 | 2 | 1 | 2 | 2 | 1 | 2 |
| 13:50 | 30,8 | 7 | 4 | 1 | 3 | 3 | 1 | 2 | 2 | 1 | 2 |
| 13:58 | 30,7 | 4 | 1 | 4 | 3 | 2 | 1 | 2 | 2 | 1 | 2 |
| 13:58 | 30,7 | 4 | 1 | 3 | 3 | 3 | 1 | 2 | 1 | 1 | 2 |
| 14:05 | 38,5 | 7 | 4 | 3 | 3 | 3 | 2 | 2 | 1 | 3 | 1 |
| 14:18 | 38,1 | 7 | 3 | 2 | 2 | 3 | 1 | 2 | 2 | 3 | 1 |
| 14:26 | 41,4 | 7 | 3 | 3 | 3 | 3 | 1 | 2 | 2 | 3 | 1 |
| 14:30 | 41,2 | 7 | 3 | 3 | 2 | 4 | 3 | 1 | 2 | 3 | 1 |
| 14:37 | 36,5 | 7 | 2 | 2 | 1 | 2 | 3 | 2 | 2 | 3 | 1 |
| 14:45 | 36,7 | 6 | 2 | 2 | 1 | 4 | 1 | 1 | 2 | 3 | 1 |
| 14:50 | 38,3 | 5 | 2 | 3 | 2 | 4 | 2 | 1 | 2 | 3 | 1 |
| 14:59 | 39,1 | 7 | 4 | 3 | 3 | 5 | 3 | 1 | 2 | 3 | 1 |
| 15:05 | 36,4 | 7 | 3 | 3 | 4 | 5 | 2 | 1 | 2 | 3 | 1 |
| 15:09 | 36,4 | 6 | 2 | 2 | 4 | 4 | 1 | 1 | 2 | 3 | 1 |
| 15:12 | 33,1 | 7 | 4 | 1 | 3 | 4 | 1 | 2 | 2 | 3 | 1 |
| 15:21 | 40,9 | 6 | 2 | 3 | 4 | 4 | 4 | 1 | 2 | 3 | 1 |
| 15:26 | 37,4 | 6 | 3 | 3 | 3 | 2 | 1 | 2 | 2 | 3 | 1 |
| 15:30 | 31,2 | 7 | 4 | 3 | 4 | 4 | 4 | 1 | 2 | 3 | 2 |
| 15:35 | 37,8 | 4 | 1 | 3 | 4 | 4 | 2 | 1 | 3 | 3 | 1 |
| 15:39 | 31,3 | 7 | 3 | 3 | 4 | 4 | 4 | 1 | 1 | 9 | 2 |
| 15:45 | 36,4 | 7 | 3 | 3 | 3 | 3 | 2 | 2 | 2 | 3 | 1 |
| 15:50 | 31,8 | 7 | 2 | 3 | 4 | 4 | 1 | 1 | 2 | 3 | 2 |
| 11:45 | 29,0 | 7 | 1 | 2 | 3 | 3 | 4 | 2 | 1 | 1 | 2 |
| 11:51 | 29,7 | 7 | 1 | 3 | 3 | 4 | 4 | 1 | 1 | 1 | 2 |
| 12:07 | 29,9 | 5 | 1 | 4 | 4 | 4 | 1 | 1 | 2 | 1 | 2 |
| 12:21 | 29,2 | 5 | 2 | 2 | 4 | 3 | 3 | 1 | 2 | 2 | 2 |
| 12:34 | 29,1 | 6 | 3 | 3 | 4 | 2 | 1 | 2 | 1 | 3 | 2 |
| 12:46 | 29,2 | 7 | 2 | 3 | 4 | 4 | 2 | 1 | 2 | 1 | 2 |
| 12:53 | 29,2 | 6 | 1 | 3 | 2 | 2 | 3 | 2 | 2 | 1 | 2 |
| 13:02 | 29,3 | 6 | 1 | 4 | 3 | 4 | 3 | 1 | 2 | 1 | 2 |
| 13:00 | 38,6 | 7 | 2 | 4 | 2 | 2 | 4 | 2 | 2 | 3 | 1 |
| 13:19 | 30,1 | 6 | 2 | 2 | 4 | 5 | 1 | 1 | 2 | 3 | 2 |
| 13:25 | 40,0 | 6 | 2 | 3 | 4 | 5 | 4 | 1 | 2 | 3 | 1 |
| 13:31 | 30,6 | 6 | 2 | 3 | 4 | 3 | 4 | 1 | 1 | 3 | 2 |
| 13:50 | 30,8 | 6 | 3 | 2 | 4 | 5 | 2 | 1 | 2 | 1 | 2 |
| 13:38 | 30,4 | 6 | 3 | 3 | 3 | 2 | 2 | 2 | 2 | 3 | 2 |
| 13:38 | 30,4 | 6 | 2 | 3 | 3 | 4 | 3 | 2 | 1 | 3 | 2 |
| 13:59 | 30,7 | 4 | 1 | 4 | 3 | 2 | 1 | 2 | 2 | 1 | 2 |
| 14:02 | 30,7 | 4 | 1 | 2 | 4 | 4 | 1 | 1 | 2 | 3 | 2 |
| 14:07 | 30,3 | 6 | 2 | 2 | 4 | 5 | 1 | 1 | 3 | 3 | 2 |
| 14:18 | 30,9 | 7 | 3 | 1 | 4 | 5 | 1 | 1 | 4 | 2 | 2 |
| 14:24 | 30,6 | 4 | 3 | 4 | 4 | 5 | 2 | 1 | 2 | 3 | 2 |
| 14:36 | 31,1 | 4 | 2 | 2 | 3 | 4 | 1 | 1 | 2 | 3 | 2 |
| 14:55 | 30,9 | 7 | 2 | 3 | 4 | 2 | 4 | 1 | 2 | 1 | 2 |
| 14:52 | 30,6 | 7 | 2 | 3 | 3 | 4 | 4 | 2 | 2 | 3 | 2 |
| 17:00 | 30,9 | 7 | 2 | 4 | 2 | 3 | 4 | 2 | 1 | 3 | 2 |
| 14:59 | 30,9 | 6 | 2 | 3 | 2 | 5 | 4 | 2 | 2 | 3 | 2 |
| 15:04 | 31,3 | 7 | 1 | 4 | 4 | 5 | 1 | 1 | 2 | 3 | 2 |
| 15:10 | 31,5 | 7 | 4 | 3 | 4 | 5 | 4 | 1 | 2 | 3 | 2 |
| 15:19 | 31,4 | 7 | 2 | 3 | 2 | 2 | 4 | 2 | 1 | 2 | 2 |
| 15:25 | 32,0 | 7 | 3 | 2 | 3 | 4 | 1 | 2 | 1 | 3 | 2 |
| 15:32 | 31,2 | 7 | 1 | 4 | 4 | 5 | 4 | 1 | 2 | 3 | 2 |
| 15:48 | 31,7 | 7 | 3 | 1 | 4 | 5 | 2 | 1 | 2 | 1 | 2 |
| 15:52 | 31,8 | 7 | 4 | 3 | 3 | 4 | 4 | 2 | 2 | 3 | 2 |
| 16:07 | 35,7 | 7 | 1 | 1 | 3 | 2 | 1 | 2 | 2 | 3 | 1 |
| 16:07 | 35,7 | 7 | 3 | 3 | 4 | 4 | 1 | 1 | 1 | 3 | 1 |
| 15:59 | 37,0 | 7 | 2 | 4 | 4 | 4 | 2 | 1 | 3 | 3 | 1 |
| 16:11 | 31,6 | 7 | 3 | 2 | 3 | 3 | 2 | 2 | 2 | 1 | 2 |
| 16:20 | 36,6 | 6 | 2 | 3 | 3 | 2 | 1 | 2 | 1 | 3 | 1 |
| 16:28 | 31,2 | 7 | 2 | 3 | 3 | 3 | 1 | 2 | 1 | 3 | 1 |
| 16:35 | 31,7 | 7 | 2 | 3 | 4 | 4 | 1 | 1 | 2 | 3 | 1 |
| 16:37 | 31,0 | 4 | 1 | 3 | 4 | 4 | 1 | 1 | 1 | 3 | 2 |
| 16:40 | 31,5 | 5 | 2 | 3 | 4 | 4 | 2 | 1 | 3 | 99 | 2 |
| 16:51 | 31,4 | 5 | 2 | 3 | 4 | 4 | 1 | 1 | 2 | 3 | 2 |
| 17:00 | 30,9 | 6 | 1 | 3 | 4 | 5 | 1 | 1 | 1 | 3 | 2 |
| 17:00 | 30,9 | 7 | 4 | 2 | 3 | 4 | 1 | 2 | 2 | 3 | 2 |
| 16:15 | 34,2 | 6 | 2 | 4 | 2 | 3 | 4 | 2 | 2 | 3 | 1 |
| 16:20 | 36,6 | 6 | 2 | 4 | 3 | 4 | 1 | 1 | 1 | 3 | 1 |
| 16:24 | 31,1 | 7 | 2 | 3 | 4 | 3 | 1 | 1 | 3 | 3 | 2 |
| 16:31 | 31,2 | 6 | 2 | 3 | 4 | 3 | 1 | 1 | 2 | 3 | 2 |
| 16:39 | 31,0 | 7 | 2 | 4 | 2 | 2 | 1 | 2 | 2 | 3 | 2 |
| 16:39 | 31,0 | 5 | 2 | 1 | 3 | 3 | 1 | 2 | 2 | 3 | 2 |
| 16:43 | 31,5 | 4 | 2 | 4 | 3 | 4 | 3 | 1 | 2 | 3 | 2 |
| 16:47 | 31,5 | 4 | 1 | 3 | 3 | 1 | 4 | 2 | 2 | 1 | 2 |
| 16:51 | 31,4 | 7 | 2 | 3 | 4 | 5 | 3 | 1 | 3 | 3 | 2 |
| 16:54 | 31,4 | 7 | 4 | 2 | 4 | 5 | 1 | 1 | 2 | 3 | 2 |
| **Sete de Setembro Square**  **13th March 2013** | | | | | | | | | | | |
| **Time** | **PET (°C)** | **Thermal**  **Perception** | **Thermal**  **Comfort**  **Evaluation** | **Preference**  **of Thermal**  **Sensation** | **Height** | **Weigh** | **Age** | **Gender** | **Clothing** | **Physical Activity** | **Position (sun/shadow)** |
| 07:00 | 26,5 | 7 | 4 | 1 | 4 | 4 | 1 | 1 | 1 | 3 | 1 |
| 07:05 | 24,0 | 6 | 2 | 2 | 4 | 1 | 3 | 1 | 2 | 2 | 1 |
| 07:08 | 24,0 | 4 | 1 | 4 | 3 | 2 | 2 | 2 | 2 | 1 | 1 |
| 07:18 | 25,9 | 6 | 2 | 3 | 1 | 3 | 3 | 1 | 2 | 99 | 1 |
| 07:30 | 25,5 | 6 | 1 | 2 | 3 | 4 | 3 | 2 | 1 | 3 | 1 |
| 07:35 | 25,6 | 5 | 1 | 3 | 3 | 2 | 3 | 2 | 2 | 3 | 1 |
| 07:38 | 25,6 | 4 | 2 | 2 | 3 | 3 | 3 | 2 | 2 | 3 | 1 |
| 07:41 | 27,7 | 5 | 2 | 3 | 4 | 1 | 5 | 1 | 2 | 1 | 1 |
| 07:44 | 27,7 | 6 | 2 | 3 | 3 | 3 | 3 | 2 | 2 | 3 | 1 |
| 07:50 | 27,2 | 7 | 3 | 3 | 4 | 1 | 4 | 1 | 3 | 1 | 1 |
| 07:05 | 26,5 | 7 | 4 | 3 | 4 | 5 | 2 | 1 | 2 | 2 | 2 |
| 07:10 | 25,4 | 7 | 2 | 3 | 4 | 3 | 3 | 1 | 3 | 2 | 2 |
| 07:10 | 25,4 | 7 | 3 | 3 | 4 | 5 | 3 | 1 | 3 | 2 | 2 |
| 07:14 | 25,4 | 4 | 1 | 3 | 4 | 4 | 2 | 1 | 2 | 3 | 2 |
| 07:18 | 26,4 | 7 | 2 | 3 | 3 | 2 | 1 | 2 | 2 | 2 | 2 |
| 07:24 | 26,1 | 7 | 2 | 3 | 3 | 5 | 2 | 1 | 1 | 2 | 2 |
| 07:24 | 26,1 | 7 | 2 | 3 | 4 | 4 | 2 | 1 | 1 | 2 | 2 |
| 07:27 | 26,6 | 7 | 1 | 3 | 4 | 4 | 3 | 1 | 2 | 2 | 2 |
| 07:31 | 26,1 | 7 | 4 | 4 | 3 | 4 | 3 | 2 | 1 | 2 | 2 |
| 07:34 | 26,1 | 7 | 2 | 3 | 2 | 2 | 2 | 2 | 2 | 2 | 2 |
| 07:38 | 26,7 | 6 | 1 | 2 | 3 | 3 | 3 | 2 | 2 | 2 | 2 |
| 07:43 | 26,7 | 7 | 2 | 1 | 3 | 3 | 2 | 2 | 2 | 2 | 2 |
| 07:47 | 28,1 | 6 | 3 | 3 | 3 | 3 | 1 | 2 | 2 | 2 | 2 |
| 07:51 | 27,5 | 7 | 4 | 3 | 2 | 3 | 4 | 2 | 2 | 2 | 2 |
| 07:55 | 27,3 | 5 | 2 | 3 | 4 | 4 | 2 | 1 | 1 | 2 | 2 |
| 07:58 | 27,3 | 7 | 3 | 3 | 3 | 2 | 2 | 2 | 2 | 2 | 2 |
| 08:01 | 28,0 | 6 | 2 | 3 | 3 | 3 | 2 | 2 | 2 | 2 | 2 |
| 08:05 | 27,8 | 7 | 3 | 2 | 4 | 3 | 2 | 2 | 2 | 2 | 2 |
| 08:10 | 27,9 | 7 | 2 | 3 | 4 | 5 | 2 | 1 | 3 | 2 | 2 |
| 08:10 | 27,9 | 6 | 2 | 2 | 4 | 4 | 2 | 1 | 2 | 2 | 2 |
| 08:00 | 31,9 | 6 | 2 | 2 | 4 | 1 | 4 | 1 | 1 | 2 | 1 |
| 08:03 | 31,9 | 7 | 3 | 2 | 4 | 1 | 4 | 1 | 2 | 1 | 1 |
| 08:06 | 33,1 | 6 | 2 | 3 | 3 | 1 | 2 | 2 | 2 | 1 | 1 |
| 08:10 | 34,0 | 5 | 2 | 4 | 3 | 5 | 1 | 1 |  | 1 | 1 |
| 08:13 | 34,0 | 7 | 3 | 3 | 2 | 1 | 1 | 2 | 2 | 1 | 1 |
| 08:16 | 32,1 | 5 | 2 | 2 | 3 | 1 | 3 | 2 | 2 | 1 | 1 |
| 08:21 | 35,1 | 4 | 1 | 3 | 3 | 1 | 4 | 1 | 1 | 1 | 1 |
| 08:25 | 33,4 | 7 | 4 | 1 | 3 | 2 | 3 | 2 | 2 | 1 | 1 |
| 08:30 | 35,7 | 7 | 2 | 3 | 4 | 1 | 3 | 1 | 2 | 1 | 1 |
| 08:35 | 35,3 | 4 | 1 | 4 | 4 | 1 | 3 | 1 | 2 | 1 | 1 |
| 08:13 | 27,9 | 4 | 1 | 4 | 4 | 4 | 3 | 1 | 2 | 2 | 2 |
| 08:16 | 27,9 | 6 | 3 | 3 | 4 | 4 | 1 | 1 | 2 | 2 | 2 |
| 08:20 | 28,1 | 7 | 3 | 3 | 3 | 5 | 2 | 2 | 1 | 2 | 2 |
| 08:24 | 28,1 | 7 | 1 | 6 | 3 | 3 | 1 | 2 | 2 | 2 | 2 |
| 08:26 | 28,0 | 5 | 1 | 2 | 3 | 3 | 1 | 2 | 1 | 2 | 2 |
| 08:30 | 28,2 | 4 | 1 | 4 | 4 | 4 | 3 | 2 | 1 | 2 | 2 |
| 08:34 | 28,2 | 7 | 4 | 2 | 2 | 3 | 3 | 2 | 2 | 2 | 2 |
| 08:36 | 27,9 | 7 | 3 | 2 | 4 | 4 | 2 | 1 | 2 | 2 | 2 |
| 08:40 | 28,6 | 7 | 2 | 4 | 3 | 4 | 4 | 2 | 2 | 2 | 2 |
| 08:45 | 28,7 | 4 | 1 | 4 | 4 | 4 | 3 | 1 | 2 | 2 | 2 |
| 08:33 | 28,2 | 6 | 2 | 3 | 3 | 3 | 3 | 2 | 2 | 3 | 3 |
| 08:49 | 28,7 | 5 | 2 | 3 | 3 | 2 | 3 | 2 | 2 | 3 | 3 |
| 09:00 | 33,1 | 4 | 1 | 4 | 3 | 3 | 2 | 2 | 2 | 1 | 1 |
| 09:08 | 31,8 | 6 | 1 | 4 | 3 | 1 | 3 | 2 | 1 | 1 | 1 |
| 09:12 | 32,6 | 5 | 1 | 2 | 4 | 1 | 5 | 1 | 2 | 1 | 1 |
| 09:15 | 33,4 | 7 | 2 | 2 | 4 | 1 | 99 | 1 | 1 | 1 | 1 |
| 09:18 | 33,4 | 7 | 2 | 2 | 2 | 1 | 2 | 2 | 1 | 1 | 1 |
| 09:23 | 32,9 | 7 | 2 | 4 | 1 | 1 | 1 | 1 | 2 | 1 | 1 |
| 09:29 | 35,4 | 7 | 2 | 2 | 4 | 1 | 3 | 1 | 2 | 1 | 1 |
| 09:33 | 36,9 | 7 | 2 | 2 | 4 | 3 | 5 | 1 | 3 | 3 | 1 |
| 09:40 | 35,7 | 6 | 2 | 3 | 4 | 5 | 1 | 1 | 3 | 3 | 1 |
| 09:45 | 31,4 | 7 | 3 | 4 | 4 | 2 | 5 | 1 | 2 | 3 | 1 |
| 08:51 | 38,3 | 7 | 2 | 3 | 4 | 5 | 4 | 1 | 2 | 3 | 1 |
| 08:54 | 29,0 | 7 | 2 | 3 | 3 | 4 | 3 | 2 | 2 | 2 | 2 |
| 08:58 | 28,8 | 7 | 4 | 2 | 4 | 4 | 4 | 1 | 2 | 2 | 2 |
| 09:00 | 29,0 | 7 | 2 | 3 | 3 | 3 | 3 | 2 | 2 | 2 | 2 |
| 09:05 | 29,7 | 7 | 4 | 3 | 3 | 3 | 3 | 2 | 1 | 2 | 2 |
| 09:09 | 29,7 | 7 | 3 | 3 | 3 | 3 | 4 | 2 | 2 | 2 | 2 |
| 09:12 | 29,5 | 7 | 3 | 3 | 4 | 5 | 2 | 1 | 1 | 2 | 2 |
| 09:15 | 29,6 | 7 | 3 | 3 | 3 | 2 | 1 | 2 | 1 | 2 | 2 |
| 09:19 | 29,6 | 7 | 2 | 4 | 4 | 3 | 1 | 2 | 2 | 2 | 2 |
| 09:23 | 28,9 | 6 | 2 | 3 | 3 | 4 | 3 | 1 | 2 | 2 | 2 |
| 09:27 | 30,0 | 7 | 4 | 2 | 4 | 5 | 1 | 1 | 1 | 2 | 2 |
| 09:32 | 30,1 | 7 | 1 | 4 | 4 | 3 | 2 | 2 | 2 | 2 | 2 |
| 09:36 | 30,6 | 5 | 2 | 3 | 4 | 3 | 3 | 1 | 2 | 2 | 2 |
| 09:39 | 30,6 | 7 | 2 | 3 | 4 | 4 | 1 | 1 | 3 | 2 | 2 |
| 09:46 | 30,1 | 7 | 3 | 2 | 4 | 4 | 1 | 2 | 2 | 1 | 2 |
| 09:51 | 29,9 | 7 | 3 | 4 | 3 | 5 | 4 | 2 | 2 | 1 | 2 |
| 09:56 | 30,4 | 6 | 3 | 4 | 3 | 6 | 1 | 2 | 2 | 1 | 2 |
| 10:00 | 29,3 | 7 | 4 | 2 | 3 | 3 | 1 | 2 | 2 | 2 | 2 |
| 10:05 | 30,1 | 5 | 1 | 3 | 3 | 3 | 2 | 2 | 2 | 2 | 2 |
| 10:00 | 30,6 | 6 | 2 | 3 | 2 | 1 | 3 | 1 | 2 | 3 | 1 |
| 10:05 | 31,1 | 7 | 3 | 4 | 4 | 2 | 5 | 1 | 2 | 1 | 1 |
| 10:09 | 31,1 | 7 | 2 | 1 | 4 | 2 | 99 | 1 | 1 | 1 | 1 |
| 10:15 | 31,1 | 6 | 3 | 3 | 2 | 4 | 1 | 2 | 2 | 3 | 1 |
| 10:23 | 31,6 | 5 | 1 | 4 | 3 | 2 | 1 | 2 | 1 | 1 | 1 |
| 10:26 | 31,2 | 7 | 3 | 2 | 3 | 1 | 3 | 2 | 2 | 3 | 1 |
| 10:33 | 30,0 | 7 | 3 | 2 | 4 | 3 | 5 | 1 | 2 | 3 | 1 |
| 10:40 | 33,0 | 6 | 3 | 3 | 4 | 1 | 4 | 1 | 1 | 3 | 1 |
| 10:44 | 33,0 | 7 | 3 | 4 | 1 | 4 | 4 | 1 | 2 | 1 | 1 |
| 10:50 | 31,7 | 7 | 3 | 4 | 4 | 1 | 4 | 1 | 1 | 1 | 1 |
| 10:09 | 30,1 | 6 | 1 | 3 | 4 | 5 | 3 | 1 | 2 | 2 | 2 |
| 10:13 | 29,9 | 5 | 2 | 3 | 4 | 5 | 2 | 1 | 1 | 2 | 2 |
| 10:17 | 30,1 | 7 | 3 | 2 | 4 | 5 | 3 | 1 | 2 | 2 | 2 |
| 10:23 | 29,8 | 7 | 2 | 3 | 3 | 3 | 2 | 2 | 2 | 2 | 2 |
| 10:26 | 29,8 | 6 | 2 | 3 | 4 | 5 | 3 | 1 | 2 | 3 | 2 |
| 10:28 | 29,8 | 7 | 4 | 2 | 99 | 99 | 99 | 2 | 1 | 2 | 2 |
| 10:32 | 30,0 | 7 | 1 | 2 | 4 | 4 | 3 | 1 | 2 | 2 | 2 |
| 10:40 | 30,9 | 6 | 2 | 3 | 4 | 4 | 2 | 1 | 2 | 3 | 2 |
| 10:44 | 30,9 | 7 | 4 | 3 | 3 | 4 | 3 | 2 | 2 | 2 | 2 |
| 10:47 | 29,5 | 5 | 2 | 2 | 3 | 4 | 1 | 1 | 2 | 2 | 2 |
| 11:00 | 30,2 | 7 | 1 | 4 | 3 | 5 | 3 | 1 | 2 | 2 | 2 |
| 11:00 | 30,2 | 7 | 1 | 4 | 4 | 4 | 3 | 1 | 2 | 2 | 2 |
| 11:03 | 30,2 | 6 | 3 | 2 | 4 | 4 | 2 | 1 | 3 | 2 | 2 |
| 11:07 | 29,9 | 7 | 3 | 3 | 3 | 3 | 2 | 2 | 1 | 2 | 2 |
| 11:10 | 30,3 | 5 | 1 | 3 | 4 | 5 | 1 | 1 | 1 | 2 | 2 |
| 11:14 | 30,3 | 7 | 3 | 3 | 4 | 4 | 2 | 2 | 2 | 3 | 2 |
| 11:19 | 30,4 | 7 | 4 | 3 | 4 | 5 | 1 | 1 | 2 | 3 | 2 |
| 11:23 | 30,6 | 6 | 1 | 3 | 4 | 3 | 3 | 2 | 2 | 2 | 2 |
| 11:28 | 30,4 | 7 | 4 | 2 | 4 | 4 | 4 | 1 | 2 | 2 | 2 |
| 11:33 | 30,6 | 7 | 3 | 3 | 4 | 4 | 2 | 2 | 1 | 3 | 2 |
| 11:44 | 35,7 | 7 | 2 | 3 | 4 | 5 | 3 | 1 | 2 | 2 | 1 |
| 11:49 | 34,1 | 6 | 2 | 4 | 4 | 4 | 1 | 1 | 1 | 2 | 1 |
| 11:51 | 34,0 | 7 | 4 | 2 | 4 | 3 | 1 | 2 | 2 | 2 | 1 |
| 11:57 | 31,7 | 7 | 2 | 3 | 4 | 4 | 3 | 1 | 1 | 3 | 2 |
| 11:00 | 31,8 | 4 | 1 | 3 | 2 | 1 | 3 | 2 | 2 | 3 | 1 |
| 11:08 | 30,8 | 6 | 1 | 4 | 4 | 2 | 4 | 2 | 2 | 3 | 1 |
| 11:13 | 30,5 | 6 | 2 | 2 | 3 | 1 | 3 | 2 | 1 | 1 | 1 |
| 11:19 | 31,4 | 6 | 2 | 2 | 3 | 5 | 2 | 1 | 2 | 3 | 1 |
| 11:32 | 31,6 | 5 | 3 | 4 | 4 | 3 | 2 | 2 | 2 | 3 | 1 |
| 11:36 | 33,0 | 7 | 3 | 4 | 4 | 3 | 1 | 2 | 2 | 1 | 1 |
| 11:40 | 35,7 | 7 | 2 | 3 | 4 | 3 | 1 | 1 | 2 | 3 | 1 |
| 11:45 | 34,1 | 4 | 1 | 3 | 4 | 5 | 1 | 1 | 2 | 1 | 1 |
| 11:50 | 34,0 | 7 | 1 | 4 | 4 | 4 | 1 | 1 | 2 | 1 | 1 |
| 11:58 | 34,6 | 7 | 3 | 2 | 4 | 4 | 2 | 2 | 2 | 3 | 1 |
| 11:08 | 29,9 | 7 | 3 | 3 | 3 | 4 | 2 | 2 | 2 | 1 | 2 |
| 11:13 | 30,3 | 7 | 2 | 1 | 4 | 4 | 2 | 2 | 2 | 1 | 2 |
| 11:16 | 30,4 | 5 | 2 | 6 | 3 | 3 | 1 | 2 | 1 | 1 | 2 |
| 11:22 | 30,6 | 7 | 3 | 3 | 2 | 1 | 1 | 2 | 2 | 2 | 2 |
| 11:22 | 30,6 | 4 | 1 | 3 | 2 | 2 | 1 | 2 | 2 | 2 | 2 |
| 11:28 | 30,4 | 7 | 2 | 4 | 2 | 2 | 1 | 2 | 2 | 2 | 2 |
| 11:36 | 31,0 | 4 | 2 | 4 | 3 | 3 | 1 | 2 | 2 | 3 | 2 |
| 11:36 | 31,0 | 7 | 2 | 4 | 3 | 4 | 1 | 2 | 2 | 3 | 2 |
| 11:40 | 30,8 | 7 | 2 | 3 | 4 | 5 | 2 | 1 | 2 | 3 | 2 |
| 11:45 | 31,0 | 7 | 3 | 4 | 3 | 3 | 3 | 2 | 2 | 3 | 2 |
| 11:45 | 31,0 | 7 | 2 | 4 | 2 | 3 | 3 | 2 | 2 | 3 | 2 |
| 12:05 | 36,6 | 7 | 3 | 4 | 3 | 4 | 2 | 2 | 2 | 3 | 1 |
| 12:10 | 35,1 | 7 | 4 | 2 | 3 | 3 | 4 | 2 | 2 | 3 | 1 |
| 12:15 | 35,5 | 7 | 3 | 2 | 3 | 4 | 2 | 2 | 1 | 3 | 1 |
| 12:19 | 35,5 | 7 | 3 | 3 | 3 | 3 | 3 | 2 | 2 | 3 | 1 |
| 12:24 | 31,9 | 7 | 3 | 3 | 3 | 3 | 3 | 2 | 2 | 2 | 2 |
| 12:30 | 32,2 | 7 | 2 | 3 | 3 | 3 | 1 | 2 | 2 | 2 | 2 |
| 12:34 | 32,2 | 7 | 4 | 3 | 3 | 3 | 3 | 2 | 2 | 2 | 2 |
| 12:39 | 31,7 | 7 | 4 | 3 | 3 | 1 | 1 | 2 | 2 | 2 | 1 |
| 12:43 | 35,0 | 7 | 3 | 3 | 3 | 1 | 3 | 2 | 2 | 1 | 1 |
| 12:52 | 38,1 | 7 | 3 | 3 | 4 | 4 | 3 | 1 | 2 | 1 | 1 |
| 12:53 | 38,1 | 7 | 3 | 2 | 4 | 4 | 3 | 2 | 2 | 2 | 1 |
| 12:58 | 39,5 | 7 | 3 | 3 | 4 | 3 | 2 | 2 | 2 | 2 | 1 |
| 13:00 | 36,4 | 7 | 3 | 3 | 4 | 4 | 2 | 1 | 2 | 2 | 1 |
| 13:05 | 33,1 | 6 | 2 | 3 | 4 | 4 | 3 | 1 | 2 | 2 | 2 |
| 13:08 | 33,1 | 7 | 4 | 2 | 3 | 3 | 1 | 2 | 2 | 2 | 2 |
| 13:10 | 35,3 | 6 | 2 | 3 | 3 | 4 | 3 | 2 | 2 | 2 | 1 |
| 13:15 | 33,3 | 7 | 4 | 2 | 4 | 3 | 1 | 2 | 1 | 2 | 2 |
| 13:18 | 33,3 | 6 | 3 | 4 | 4 | 4 | 2 | 1 | 2 | 2 | 2 |
| 13:20 | 34,8 | 7 | 4 | 1 | 4 | 4 | 1 | 1 | 2 | 2 | 2 |
| 13:25 | 39,4 | 7 | 3 | 2 | 3 | 4 | 3 | 2 | 2 | 2 | 1 |
| 13:29 | 39,4 | 7 | 3 | 3 | 3 | 3 | 1 | 2 | 2 | 2 | 1 |
| 13:31 | 33,4 | 7 | 1 | 4 | 4 | 5 | 3 | 1 | 2 | 2 | 2 |
| 13:34 | 33,4 | 7 | 2 | 3 | 3 | 3 | 1 | 2 | 2 | 2 | 2 |
| 13:00 | 32,3 | 7 | 4 | 2 | 3 | 3 | 3 | 2 | 2 | 2 | 2 |
| 13:05 | 33,1 | 7 | 4 | 3 | 3 | 3 | 3 | 2 | 2 | 2 | 2 |
| 13:10 | 33,1 | 7 | 4 | 2 | 4 | 4 | 1 | 1 | 2 | 2 | 2 |
| 13:15 | 33,3 | 6 | 3 | 2 | 4 | 5 | 1 | 1 | 2 | 1 | 2 |
| 13:20 | 34,8 | 6 | 3 | 3 | 4 | 5 | 3 | 1 | 3 | 2 | 2 |
| 13:25 | 34,9 | 7 | 3 | 3 | 3 | 5 | 3 | 1 | 2 | 1 | 2 |
| 13:30 | 33,4 | 6 | 2 | 1 | 4 | 5 | 1 | 1 | 2 | 1 | 2 |
| 13:45 | 32,8 | 7 | 3 | 1 | 4 | 5 | 1 | 1 | 2 | 1 | 2 |
| 13:50 | 34,0 | 6 | 2 | 3 | 4 | 5 | 2 | 1 | 3 | 1 | 2 |
| 13:57 | 38,5 | 6 | 2 | 3 | 4 | 5 | 3 | 1 | 2 | 2 | 2 |
| 13:38 | 33,4 | 6 | 1 | 4 | 4 | 5 | 1 | 2 | 2 | 2 | 2 |
| 13:38 | 33,4 | 7 | 2 | 3 | 4 | 3 | 1 | 2 | 2 | 2 | 2 |
| 13:43 | 32,6 | 7 | 1 | 4 | 4 | 4 | 1 | 1 | 1 | 2 | 2 |
| 13:45 | 32,8 | 7 | 2 | 4 | 4 | 4 | 1 | 2 | 2 | 2 | 2 |
| 13:50 | 34,0 | 7 | 2 | 4 | 4 | 4 | 1 | 1 | 2 | 2 | 2 |
| 13:54 | 34,0 | 7 | 2 | 3 | 4 | 4 | 1 | 1 | 1 | 3 | 2 |
| 13:57 | 38,5 | 7 | 4 | 3 | 4 | 4 | 2 | 2 | 2 | 2 | 2 |
| 13:59 | 38,5 | 7 | 3 | 3 | 3 | 4 | 3 | 2 | 1 | 2 | 2 |
| 14:05 | 37,6 | 7 | 4 | 2 | 4 | 5 | 2 | 1 | 2 | 1 | 2 |
| 14:10 | 38,6 | 7 | 4 | 2 | 4 | 4 | 1 | 1 | 2 | 2 | 2 |
| 14:00 | 35,6 | 7 | 4 | 2 | 3 | 3 | 2 | 2 | 2 | 1 | 2 |
| 14:05 | 37,6 | 7 | 4 | 3 | 3 | 3 | 2 | 2 | 2 | 2 | 2 |
| 14:09 | 37,6 | 7 | 4 | 3 | 3 | 3 | 1 | 2 | 2 | 1 | 2 |
| 14:15 | 33,1 | 7 | 4 | 3 | 4 | 5 | 3 | 1 | 2 | 2 | 2 |
| 14:30 | 34,0 | 7 | 4 | 2 | 3 | 4 | 3 | 2 | 2 | 1 | 2 |
| 14:35 | 33,4 | 7 | 1 | 4 | 4 | 4 | 1 | 1 | 1 | 1 | 2 |
| 14:40 | 33,2 | 7 | 4 | 2 | 4 | 5 | 1 | 1 | 2 | 1 | 2 |
| 14:45 | 33,6 | 7 | 4 | 3 | 3 | 3 | 2 | 2 | 2 | 1 | 2 |
| 14:50 | 33,2 | 6 | 3 | 3 | 4 | 5 | 2 | 1 | 2 | 1 | 2 |
| 14:55 | 33,3 | 7 | 3 | 3 | 3 | 4 | 2 | 2 | 2 | 1 | 2 |
| 14:20 | 33,1 | 7 | 2 | 2 | 4 | 4 | 2 | 1 | 2 | 2 | 2 |
| 14:24 | 39,0 | 7 | 3 | 4 | 3 | 3 | 1 | 2 | 2 | 2 | 1 |
| 14:27 | 33,2 | 7 | 4 | 3 | 4 | 4 | 2 | 1 | 2 | 3 | 2 |
| 14:31 | 34,0 | 7 | 3 | 2 | 4 | 5 | 3 | 1 | 3 | 2 | 2 |
| 14:36 | 33,4 | 7 | 2 | 2 | 3 | 3 | 2 | 2 | 2 | 2 | 2 |
| 14:39 | 36,4 | 7 | 3 | 3 | 2 | 4 | 1 | 2 | 2 | 2 | 1 |
| 14:44 | 33,2 | 7 | 4 | 2 | 3 | 4 | 1 | 2 | 3 | 2 | 2 |
| 14:48 | 33,6 | 7 | 4 | 3 | 3 | 4 | 2 | 2 | 2 | 2 | 2 |
| 14:48 | 33,6 | 7 | 4 | 3 | 3 | 3 | 3 | 2 | 2 | 2 | 2 |
| 14:51 | 33,2 | 7 | 3 | 3 | 4 | 4 | 2 | 1 | 2 | 3 | 2 |
| 14:55 | 33,3 | 7 | 4 | 3 | 99 | 99 | 99 | 2 | 99 | 2 | 2 |
| 15:00 | 33,8 | 7 | 4 | 2 | 3 | 3 | 2 | 2 | 2 | 2 | 2 |
| 15:06 | 35,5 | 7 | 3 | 2 | 3 | 3 | 3 | 2 | 3 | 2 | 2 |
| 15:11 | 34,2 | 7 | 4 | 2 | 2 | 3 | 3 | 2 | 2 | 2 | 2 |
| 15:14 | 37,3 | 7 | 2 | 3 | 3 | 3 | 1 | 2 | 2 | 2 | 1 |
| 15:19 | 39,8 | 7 | 2 | 2 | 4 | 5 | 2 | 1 | 2 | 2 | 1 |
| 15:23 | 33,1 | 7 | 4 | 2 | 4 | 5 | 3 | 1 | 2 | 2 | 2 |
| 15:28 | 33,5 | 7 | 3 | 2 | 4 | 4 | 1 | 1 | 2 | 2 | 2 |
| 15:31 | 33,2 | 7 | 3 | 4 | 4 | 5 | 1 | 1 | 2 | 2 | 1 |
| 15:34 | 33,1 | 7 | 3 | 3 | 3 | 4 | 1 | 1 | 2 | 2 | 2 |
| 15:05 | 35,5 | 7 | 4 | 2 | 3 | 3 | 3 | 2 | 2 | 3 | 2 |
| 15:10 | 34,2 | 7 | 2 | 2 | 4 | 4 | 1 | 1 | 3 | 2 | 2 |
| 15:15 | 33,4 | 6 | 3 | 3 | 4 | 5 | 2 | 1 | 2 | 2 | 2 |
| 15:20 | 33,1 | 6 | 2 | 3 | 3 | 2 | 1 | 2 | 2 | 1 | 2 |
| 15:25 | 33,5 | 7 | 2 | 3 | 4 | 4 | 2 | 1 | 2 | 1 | 2 |
| 15:30 | 33,1 | 7 | 3 | 2 | 3 | 4 | 1 | 2 | 1 | 2 | 2 |
| 15:35 | 32,7 | 7 | 3 | 3 | 3 | 3 | 3 | 2 | 2 | 2 | 2 |
| 15:40 | 32,7 | 7 | 4 | 1 | 4 | 3 | 5 | 1 | 3 | 3 | 2 |
| 15:45 | 32,4 | 7 | 4 | 3 | 2 | 3 | 3 | 2 | 2 | 2 | 2 |
| 15:57 | 32,6 | 7 | 3 | 3 | 3 | 4 | 2 | 2 | 2 | 2 | 2 |
| 15:38 | 32,7 | 7 | 4 | 2 | 4 | 5 | 1 | 1 | 2 | 3 | 2 |
| 15:49 | 32,4 | 7 | 3 | 3 | 4 | 4 | 2 | 2 | 2 | 2 | 2 |
| 15:54 | 33,7 | 7 | 2 | 3 | 4 | 4 | 1 | 1 | 2 | 2 | 1 |
| 15:58 | 32,6 | 7 | 3 | 2 | 4 | 4 | 2 | 2 | 1 | 2 | 2 |
| 16:02 | 33,2 | 7 | 3 | 3 | 4 | 4 | 2 | 1 | 2 | 2 | 2 |
| 16:10 | 32,7 | 7 | 2 | 3 | 4 | 5 | 3 | 2 | 2 | 2 | 2 |
| 16:14 | 32,7 | 7 | 3 | 3 | 4 | 3 | 1 | 2 | 2 | 2 | 2 |
| 16:20 | 38,5 | 7 | 4 | 3 | 3 | 3 | 2 | 2 | 2 | 2 | 1 |
| 16:25 | 32,9 | 7 | 3 | 3 | 4 | 4 | 2 | 1 | 2 | 3 | 2 |
| 16:28 | 32,9 | 7 | 1 | 4 | 4 | 5 | 2 | 1 | 1 | 2 | 2 |
| 16:00 | 33,2 | 7 | 3 | 3 | 2 | 5 | 3 | 2 | 2 | 1 | 2 |
| 16:05 | 33,0 | 7 | 4 | 1 | 4 | 5 | 1 | 1 | 2 | 1 | 2 |
| 16:10 | 32,7 | 7 | 4 | 3 | 2 | 2 | 1 | 2 | 2 | 1 | 2 |
| 16:15 | 32,4 | 7 | 3 | 2 | 3 | 3 | 1 | 2 | 2 | 2 | 2 |
| 16:20 | 32,4 | 5 | 1 | 4 | 4 | 4 | 3 | 2 | 2 | 2 | 2 |
| 16:25 | 32,9 | 6 | 1 | 3 | 4 | 5 | 2 | 1 | 2 | 2 | 2 |
| 16:30 | 32,7 | 7 | 4 | 3 | 4 | 2 | 4 | 2 | 2 | 3 | 2 |
| 16:35 | 33,6 | 7 | 4 | 2 | 3 | 2 | 3 | 1 | 1 | 3 | 2 |
| 16:40 | 33,0 | 7 | 3 | 3 | 3 | 4 | 3 | 2 | 2 | 3 | 2 |
| 16:45 | 32,7 | 7 | 4 | 3 | 5 | 4 | 3 | 1 | 2 | 2 | 2 |
| 16:50 | 32,3 | 7 | 4 | 3 | 4 | 5 | 4 | 1 | 2 | 3 | 2 |
| 16:30 | 32,7 | 7 | 2 | 3 | 3 | 3 | 3 | 2 | 1 | 2 | 2 |
| 16:36 | 33,6 | 7 | 2 | 3 | 4 | 4 | 2 | 1 | 2 | 3 | 2 |
| 16:41 | 33,0 | 7 | 4 | 1 | 4 | 3 | 2 | 2 | 2 | 2 | 2 |
| 16:45 | 32,7 | 7 | 2 | 4 | 3 | 3 | 2 | 2 | 2 | 2 | 2 |
| 16:49 | 32,7 | 7 | 4 | 4 | 4 | 5 | 2 | 1 | 2 | 2 | 2 |
| 16:52 | 32,3 | 7 | 4 | 3 | 4 | 5 | 1 | 1 | 2 | 2 | 2 |
| 14:45 | 40,4 | 7 | 3 | 3 | 3 | 3 | 1 | 1 | 2 | 3 | 1 |
| 08:55 | 35,5 | 7 | 4 | 3 | 4 | 2 | 1 | 2 | 2 | 2 | 1 |
| 08:55 | 35,5 | 7 | 3 | 3 | 2 | 1 | 1 | 2 | 2 | 2 | 1 |
| 09:01 | 33,1 | 7 | 2 | 2 | 2 | 3 | 2 | 2 | 2 | 2 | 1 |
| 10:11 | 32,0 | 7 | 2 | 2 | 4 | 5 | 1 | 1 | 1 | 3 | 1 |
| 10:36 | 30,0 | 7 | 2 | 4 | 4 | 5 | 4 | 1 | 2 | 3 | 2 |
| 10:46 | 29,5 | 4 | 1 | 4 | 3 | 4 | 3 | 1 | 2 | 2 | 2 |
| 11:31 | 30,6 | 7 | 3 | 2 | 4 | 1 | 4 | 1 | 2 | 2 | 2 |
| 11:44 | 30,8 | 7 | 3 | 2 | 4 | 4 | 4 | 1 | 1 | 2 | 2 |
| 08:35 | 27,9 | 5 | 2 | 2 | 2 | 1 | 2 | 2 | 2 | 3 | 2 |
| 08:50 | 29,0 | 5 | 1 | 4 | 4 | 4 | 4 | 1 | 2 | 2 | 2 |
| 07:04 | 26,6 | 4 | 1 | 4 | 4 | 5 | 1 | 1 | 3 | 3 | 2 |
| 07:07 | 26,5 | 7 | 1 | 4 | 3 | 4 | 4 | 1 | 3 | 3 | 2 |
| 07:10 | 25,4 | 7 | 3 | 4 | 2 | 3 | 4 | 1 | 3 | 3 | 2 |
| 07:15 | 26,4 | 4 | 1 | 4 | 1 | 1 | 1 | 2 | 2 | 3 | 2 |
| 07:18 | 26,4 | 4 | 1 | 4 | 4 | 4 | 2 | 1 | 3 | 3 | 2 |
| 07:22 | 26,1 | 7 | 3 | 1 | 4 | 4 | 3 | 1 | 3 | 3 | 2 |
| 07:25 | 26,6 | 7 | 3 | 2 | 4 | 4 | 2 | 2 | 3 | 3 | 2 |
| 07:40 | 26,7 | 4 | 1 | 4 | 4 | 4 | 3 | 1 | 3 | 3 | 2 |
| 07:50 | 27,5 | 4 | 1 | 4 | 3 | 3 | 3 | 2 | 3 | 3 | 2 |
| 08:00 | 28,0 | 4 | 1 | 4 | 4 | 4 | 3 | 2 | 2 | 3 | 2 |
| 07:05 | 26,5 | 5 | 3 | 2 | 2 | 2 | 2 | 2 | 2 | 1 | 2 |
| 07:09 | 26,5 | 6 | 3 | 2 | 4 | 5 | 3 | 1 | 3 | 1 | 2 |
| 07:12 | 25,4 | 6 | 3 | 1 | 3 | 4 | 3 | 1 | 3 | 1 | 2 |
| 07:15 | 26,4 | 6 | 1 | 2 | 4 | 5 | 3 | 1 | 2 | 1 | 2 |
| 07:19 | 26,4 | 4 | 1 | 3 | 4 | 5 | 2 | 1 | 3 | 1 | 2 |
| 07:23 | 26,1 | 5 | 1 | 1 | 3 | 5 | 1 | 1 | 3 | 1 | 2 |
| 07:27 | 26,6 | 5 | 2 | 2 | 2 | 3 | 1 | 2 | 2 | 1 | 2 |
| 07:32 | 26,1 | 5 | 1 | 3 | 3 | 4 | 1 | 2 | 2 | 1 | 2 |
| 07:36 | 26,7 | 6 | 3 | 3 | 4 | 5 | 3 | 1 | 3 | 2 | 2 |
| 07:42 | 26,7 | 6 | 3 | 1 | 2 | 2 | 3 | 2 | 2 | 2 | 2 |
| 08:05 | 27,8 | 6 | 3 | 2 | 4 | 5 | 3 | 1 | 3 | 1 | 2 |
| 08:09 | 27,8 | 5 | 1 | 2 | 4 | 4 | 1 | 1 | 3 | 2 | 2 |
| 08:13 | 27,9 | 5 | 2 | 1 | 2 | 3 | 1 | 2 | 3 | 2 | 2 |
| 08:17 | 27,9 | 6 | 1 | 2 | 4 | 5 | 2 | 1 | 2 | 2 | 2 |
| 08:23 | 28,1 | 5 | 1 | 3 | 3 | 4 | 3 | 1 | 2 | 1 | 2 |
| 08:27 | 28,0 | 6 | 3 | 1 | 2 | 4 | 2 | 2 | 2 | 2 | 2 |
| 08:33 | 28,2 | 5 | 2 | 1 | 4 | 4 | 3 | 1 | 2 | 1 | 2 |
| 08:39 | 27,9 | 7 | 4 | 2 | 4 | 4 | 1 | 1 | 3 | 1 | 2 |
| 08:44 | 36,8 | 7 | 2 | 1 | 4 | 5 | 3 | 1 | 3 | 1 | 1 |
| 08:56 | 35,5 | 5 | 1 | 2 | 3 | 4 | 2 | 2 | 2 | 2 | 1 |
| 08:07 | 27,8 | 5 | 2 | 2 | 4 | 5 | 4 | 1 | 3 | 3 | 2 |
| 08:11 | 27,9 | 5 | 1 | 2 | 3 | 3 | 2 | 1 | 3 | 1 | 2 |
| 08:19 | 27,9 | 7 | 4 | 2 | 4 | 5 | 2 | 1 | 3 | 1 | 2 |
| 09:40 | 30,8 | 6 | 1 | 4 | 4 | 4 | 2 | 1 | 3 | 3 | 2 |
| 09:43 | 30,8 | 6 | 1 | 4 | 4 | 3 | 4 | 1 | 3 | 3 | 2 |
| 10:15 | 30,1 | 7 | 2 | 3 | 2 | 3 | 4 | 2 | 3 | 3 | 2 |
| 10:30 | 30,0 | 4 | 2 | 4 | 3 | 4 | 1 | 1 | 3 | 1 | 2 |
| 11:40 | 30,8 | 7 | 2 | 2 | 3 | 3 | 3 | 1 | 3 | 2 | 2 |
| 09:05 | 29,7 | 4 | 1 | 2 | 2 | 3 | 3 | 2 | 1 | 1 | 2 |
| 09:11 | 29,5 | 7 | 3 | 1 | 3 | 3 | 3 | 2 | 2 | 1 | 2 |
| 09:17 | 29,6 | 4 | 1 | 2 | 2 | 2 | 1 | 1 | 2 | 1 | 2 |
| 09:24 | 28,9 | 7 | 3 | 1 | 3 | 4 | 2 | 2 | 1 | 1 | 2 |
| 09:30 | 36,9 | 7 | 4 | 1 | 4 | 5 | 3 | 1 | 1 | 1 | 1 |
| 09:37 | 30,6 | 6 | 3 | 2 | 4 | 5 | 4 | 1 | 2 | 1 | 2 |
| 09:42 | 35,7 | 6 | 2 | 2 | 4 | 5 | 1 | 1 | 2 | 2 | 1 |
| 09:47 | 30,1 | 7 | 3 | 2 | 3 | 4 | 3 | 1 | 2 | 2 | 2 |
| 09:54 | 30,3 | 4 | 1 | 2 | 4 | 5 | 3 | 1 | 3 | 1 | 1 |
| 09:58 | 30,4 | 4 | 1 | 2 | 2 | 3 | 1 | 2 | 2 | 2 | 2 |
| 09:05 | 29,7 | 7 | 2 | 1 | 4 | 5 | 3 | 1 | 3 | 1 | 2 |
| 09:15 | 29,6 | 6 | 3 | 3 | 4 | 3 | 1 | 1 | 2 | 2 | 2 |
| 09:25 | 30,0 | 4 | 3 | 3 | 2 | 4 | 1 | 2 | 3 | 2 | 2 |
| 09:29 | 30,0 | 5 | 2 | 4 | 4 | 4 | 1 | 1 | 3 | 2 | 2 |
| 09:33 | 30,1 | 7 | 1 | 4 | 3 | 2 | 3 | 1 | 2 | 2 | 2 |
| 09:39 | 30,6 | 7 | 3 | 3 | 4 | 3 | 2 | 1 | 3 | 2 | 2 |
| 09:45 | 30,1 | 7 | 3 | 4 | 2 | 2 | 1 | 2 | 3 | 1 | 2 |
| 09:49 | 30,1 | 7 | 3 | 4 | 3 | 2 | 1 | 2 | 3 | 1 | 2 |
| 09:52 | 29,9 | 7 | 4 | 1 | 4 | 5 | 3 | 1 | 3 | 99 | 2 |
| 09:59 | 30,4 | 7 | 2 | 3 | 3 | 4 | 4 | 1 | 3 | 3 | 2 |
| 10:05 | 30,1 | 6 | 3 | 1 | 4 | 5 | 2 | 1 | 2 | 2 | 2 |
| 10:11 | 29,9 | 7 | 4 | 2 | 3 | 3 | 3 | 1 | 2 | 1 | 2 |
| 10:17 | 30,1 | 6 | 2 | 2 | 4 | 4 | 1 | 1 | 2 | 1 | 2 |
| 10:25 | 29,8 | 7 | 3 | 2 | 2 | 3 | 4 | 2 | 2 | 2 | 2 |
| 10:29 | 29,8 | 4 | 1 | 4 | 4 | 4 | 1 | 1 | 1 | 1 | 2 |
| 10:35 | 30,0 | 7 | 3 | 2 | 3 | 3 | 1 | 2 | 2 | 2 | 2 |
| 10:39 | 30,0 | 7 | 3 | 2 | 4 | 5 | 3 | 1 | 2 | 2 | 2 |
| 10:45 | 29,5 | 4 | 1 | 2 | 4 | 4 | 3 | 1 | 3 | 1 | 2 |
| 10:52 | 30,1 | 6 | 2 | 2 | 4 | 5 | 2 | 1 | 3 | 2 | 2 |
| 10:58 | 29,8 | 6 | 2 | 1 | 3 | 3 | 1 | 2 | 2 | 1 | 2 |
| 10:10 | 29,9 | 7 | 4 | 3 | 3 | 5 | 2 | 1 | 2 | 1 | 2 |
| 10:14 | 29,9 | 7 | 2 | 2 | 4 | 4 | 2 | 1 | 3 | 1 | 2 |
| 10:19 | 30,1 | 7 | 2 | 2 | 4 | 3 | 2 | 1 | 3 | 1 | 2 |
| 10:25 | 29,8 | 7 | 2 | 3 | 3 | 4 | 3 | 2 | 3 | 1 | 2 |
| 10:29 | 29,8 | 7 | 3 | 7 | 3 | 3 | 4 | 2 | 2 | 2 | 2 |
| 10:34 | 30,0 | 7 | 3 | 2 | 4 | 3 | 3 | 1 | 3 | 1 | 2 |
| 10:41 | 30,9 | 7 | 4 | 2 | 3 | 4 | 1 | 2 | 2 | 1 | 2 |
| 10:48 | 29,5 | 4 | 1 | 4 | 3 | 2 | 1 | 2 | 2 | 3 | 2 |
| 10:52 | 30,1 | 7 | 4 | 3 | 4 | 4 | 1 | 2 | 3 | 1 | 2 |
| 10:57 | 29,8 | 7 | 3 | 3 | 4 | 4 | 1 | 2 | 3 | 1 | 2 |
| 11:10 | 30,3 | 4 | 1 | 2 | 4 | 5 | 1 | 2 | 1 | 2 | 2 |
| 11:17 | 30,4 | 4 | 1 | 2 | 4 | 4 | 3 | 2 | 3 | 1 | 2 |
| 11:26 | 30,4 | 6 | 4 | 1 | 4 | 5 | 2 | 1 | 2 | 2 | 2 |
| 11:32 | 30,6 | 4 | 1 | 1 | 4 | 5 | 1 | 1 | 2 | 2 | 2 |
| 11:38 | 31,0 | 6 | 2 | 2 | 3 | 4 | 1 | 1 | 2 | 2 | 2 |
| 11:44 | 30,8 | 6 | 2 | 2 | 3 | 3 | 1 | 2 | 1 | 1 | 2 |
| 11:50 | 31,0 | 5 | 3 | 2 | 4 | 5 | 2 | 2 | 2 | 1 | 2 |
| 11:53 | 31,0 | 7 | 4 | 2 | 4 | 4 | 2 | 2 | 2 | 1 | 2 |
| 11:56 | 31,7 | 4 | 2 | 1 | 4 | 5 | 2 | 1 | 3 | 99 | 2 |
| 11:59 | 31,7 | 6 | 2 | 2 | 3 | 4 | 1 | 2 | 2 | 99 | 2 |
| 11:05 | 29,9 | 7 | 3 | 3 | 4 | 4 | 1 | 2 | 3 | 1 | 2 |
| 11:15 | 30,4 | 3 | 1 | 4 | 3 | 3 | 2 | 1 | 3 | 3 | 2 |
| 11:20 | 30,6 | 7 | 2 | 4 | 3 | 4 | 2 | 2 | 3 | 1 | 2 |
| 11:25 | 30,4 | 5 | 1 | 4 | 4 | 3 | 4 | 1 | 3 | 3 | 2 |
| 11:34 | 30,6 | 4 | 1 | 4 | 4 | 3 | 3 | 1 | 3 | 1 | 2 |
| 11:48 | 31,0 | 3 | 2 | 4 | 3 | 3 | 3 | 1 | 3 | 1 | 2 |
| 11:53 | 31,0 | 7 | 4 | 1 | 4 | 5 | 1 | 1 | 3 | 3 | 2 |
| 11:57 | 31,7 | 7 | 4 | 3 | 4 | 3 | 1 | 1 | 3 | 3 | 2 |
| 12:00 | 32,7 | 6 | 3 | 3 | 3 | 3 | 2 | 1 | 3 | 1 | 2 |
| 12:03 | 32,7 | 4 | 1 | 4 | 4 | 5 | 1 | 1 | 3 | 1 | 2 |
| 12:15 | 31,6 | 5 | 2 | 4 | 4 | 5 | 4 | 1 | 3 | 2 | 2 |
| 12:18 | 31,6 | 4 | 4 | 4 | 4 | 3 | 1 | 1 | 3 | 3 | 2 |
| 12:23 | 31,9 | 7 | 4 | 1 | 4 | 3 | 1 | 2 | 2 | 1 | 2 |
| 12:29 | 32,3 | 7 | 2 | 3 | 4 | 2 | 1 | 1 | 3 | 1 | 2 |
| 12:33 | 32,2 | 7 | 2 | 3 | 4 | 3 | 1 | 1 | 2 | 1 | 2 |
| 12:39 | 31,8 | 7 | 2 | 3 | 4 | 4 | 2 | 1 | 3 | 1 | 2 |
| 12:42 | 31,3 | 7 | 2 | 3 | 4 | 5 | 4 | 1 | 3 | 1 | 2 |
| 12:47 | 32,8 | 7 | 2 | 4 | 2 | 4 | 2 | 1 | 3 | 1 | 2 |
| 12:51 | 32,8 | 7 | 2 | 4 | 99 | 2 | 1 | 1 | 3 | 2 | 2 |
| 12:57 | 32,1 | 7 | 2 | 4 | 99 | 3 | 1 | 1 | 3 | 2 | 2 |
| 12:10 | 31,9 | 7 | 2 | 2 | 4 | 5 | 4 | 1 | 2 | 2 | 2 |
| 12:10 | 31,9 | 7 | 2 | 3 | 4 | 5 | 4 | 1 | 2 | 2 | 2 |
| 12:17 | 31,6 | 6 | 3 | 3 | 4 | 3 | 1 | 1 | 2 | 1 | 2 |
| 12:23 | 31,9 | 7 | 1 | 3 | 4 | 4 | 4 | 1 | 2 | 3 | 2 |
| 12:27 | 32,3 | 7 | 4 | 2 | 4 | 4 | 3 | 1 | 2 | 3 | 2 |
| 12:32 | 32,2 | 6 | 2 | 3 | 3 | 2 | 1 | 2 | 2 | 3 | 2 |
| 12:39 | 31,8 | 6 | 1 | 3 | 2 | 3 | 2 | 2 | 1 | 3 | 2 |
| 12:43 | 31,3 | 6 | 3 | 3 | 3 | 3 | 4 | 2 | 1 | 3 | 2 |
| 12:51 | 32,8 | 7 | 3 | 3 | 3 | 2 | 2 | 2 | 2 | 3 | 2 |
| 12:55 | 32,1 | 7 | 4 | 4 | 3 | 2 | 1 | 2 | 2 | 3 | 2 |
| 12:25 | 38,6 | 7 | 1 | 2 | 4 | 4 | 4 | 1 | 2 | 2 | 1 |
| 12:54 | 32,8 | 7 | 4 | 3 | 3 | 4 | 4 | 1 | 3 | 2 | 2 |
| 13:05 | 37,4 | 7 | 3 | 3 | 3 | 3 | 2 | 1 | 4 | 2 | 1 |
| 13:10 | 33,1 | 7 | 4 | 3 | 3 | 2 | 2 | 1 | 1 | 99 | 2 |
| 13:15 | 40,3 | 7 | 3 | 3 | 3 | 3 | 1 | 1 | 1 | 99 | 1 |
| 13:20 | 37,0 | 7 | 3 | 2 | 3 | 5 | 2 | 1 | 3 | 99 | 1 |
| 13:30 | 33,4 | 7 | 2 | 3 | 4 | 5 | 2 | 1 | 2 | 99 | 2 |
| 13:35 | 33,4 | 7 | 3 | 3 | 1 | 2 | 1 | 2 | 2 | 99 | 2 |
| 13:45 | 32,8 | 7 | 3 | 1 | 4 | 5 | 3 | 99 | 3 | 99 | 2 |
| 13:50 | 34,0 | 7 | 2 | 3 | 2 | 2 | 2 | 2 | 1 | 99 | 2 |
| 13:05 | 33,1 | 6 | 2 | 3 | 4 | 4 | 4 | 1 | 2 | 1 | 2 |
| 13:16 | 33,3 | 7 | 2 | 2 | 3 | 4 | 3 | 1 | 2 | 2 | 2 |
| 13:21 | 37,0 | 4 | 1 | 4 | 4 | 5 | 3 | 1 | 2 | 2 | 1 |
| 13:27 | 34,9 | 7 | 3 | 1 | 3 | 3 | 4 | 2 | 2 | 2 | 2 |
| 13:30 | 33,4 | 7 | 2 | 3 | 2 | 2 | 3 | 2 | 2 | 1 | 2 |
| 13:35 | 33,4 | 7 | 2 | 3 | 3 | 5 | 2 | 2 | 2 | 1 | 2 |
| 13:37 | 33,4 | 7 | 3 | 3 | 4 | 5 | 2 | 1 | 2 | 2 | 2 |
| 13:45 | 32,8 | 7 | 3 | 4 | 4 | 5 | 3 | 1 | 2 | 2 | 2 |
| 13:54 | 39,4 | 6 | 2 | 2 | 2 | 3 | 2 | 1 | 4 | 3 | 1 |
| 13:54 | 39,4 | 7 | 3 | 2 | 2 | 2 | 1 | 2 | 4 | 3 | 1 |
| 13:02 | 32,3 | 7 | 2 | 2 | 2 | 3 | 1 | 1 | 3 | 1 | 2 |
| 13:05 | 33,1 | 7 | 2 | 2 | 4 | 3 | 1 | 1 | 2 | 2 | 2 |
| 13:09 | 33,1 | 6 | 2 | 4 | 4 | 3 | 1 | 1 | 3 | 2 | 2 |
| 13:15 | 40,3 | 7 | 3 | 4 | 2 | 2 | 2 | 1 | 3 | 3 | 1 |
| 13:20 | 34,8 | 7 | 4 | 2 | 4 | 4 | 1 | 1 | 3 | 1 | 2 |
| 13:25 | 34,9 | 6 | 2 | 2 | 4 | 5 | 2 | 1 | 3 | 1 | 2 |
| 13:41 | 32,6 | 7 | 4 | 4 | 4 | 5 | 1 | 1 | 3 | 1 | 2 |
| 13:46 | 32,8 | 7 | 4 | 1 | 2 | 1 | 1 | 2 | 3 | 2 | 2 |
| 13:50 | 34,0 | 7 | 4 | 1 | 4 | 3 | 2 | 2 | 3 | 1 | 2 |
| 13:59 | 38,5 | 7 | 4 | 1 | 5 | 5 | 2 | 2 | 3 | 1 | 2 |
| 14:07 | 37,6 | 7 | 3 | 2 | 3 | 4 | 1 | 2 | 2 | 1 | 2 |
| 14:11 | 38,6 | 6 | 3 | 3 | 2 | 1 | 1 | 2 | 2 | 1 | 2 |
| 14:11 | 38,6 | 4 | 1 | 4 | 3 | 4 | 1 | 2 | 2 | 1 | 2 |
| 14:17 | 33,1 | 6 | 3 | 3 | 3 | 3 | 2 | 2 | 1 | 1 | 2 |
| 14:38 | 33,4 | 7 | 4 | 1 | 2 | 2 | 1 | 2 | 2 | 2 | 2 |
| 14:42 | 33,2 | 7 | 3 | 3 | 3 | 4 | 4 | 2 | 1 | 2 | 2 |
| 14:49 | 33,6 | 7 | 2 | 3 | 2 | 1 | 1 | 2 | 2 | 2 | 2 |
| 14:49 | 33,6 | 7 | 2 | 3 | 3 | 3 | 1 | 2 | 2 | 2 | 2 |
| 15:11 | 34,2 | 6 | 1 | 1 | 2 | 1 | 1 | 2 | 2 | 1 | 2 |
| 15:11 | 34,2 | 6 | 1 | 2 | 2 | 1 | 1 | 2 | 2 | 1 | 2 |
| 14:10 | 38,6 | 7 | 2 | 3 | 4 | 3 | 1 | 1 | 1 | 1 | 2 |
| 14:13 | 38,6 | 7 | 1 | 3 | 4 | 2 | 1 | 1 | 3 | 1 | 2 |
| 14:20 | 33,1 | 7 | 4 | 2 | 4 | 4 | 2 | 1 | 1 | 3 | 2 |
| 14:25 | 33,2 | 4 | 1 | 2 | 4 | 2 | 1 | 1 | 3 | 2 | 2 |
| 14:32 | 34,0 | 7 | 4 | 2 | 4 | 3 | 1 | 1 | 1 | 1 | 2 |
| 14:35 | 33,4 | 7 | 4 | 2 | 3 | 1 | 1 | 2 | 2 | 1 | 2 |
| 14:40 | 33,2 | 7 | 2 | 2 | 4 | 5 | 4 | 1 | 3 | 3 | 2 |
| 14:45 | 40,4 | 7 | 3 | 3 | 4 | 5 | 2 | 1 | 1 | 3 | 1 |
| 14:50 | 42,4 | 7 | 4 | 2 | 4 | 5 | 1 | 1 | 1 | 3 | 1 |
| 14:55 | 38,0 | 7 | 4 | 2 | 4 | 5 | 1 | 1 | 3 | 3 | 1 |
| 12:45 | 32,8 | 7 | 4 | 3 | 3 | 5 | 3 | 2 | 1 | 2 | 2 |
| 14:00 | 35,6 | 7 | 2 | 2 | 3 | 5 | 4 | 1 | 1 | 99 | 2 |
| 14:05 | 37,6 | 5 | 1 | 1 | 4 | 5 | 4 | 1 | 1 | 99 | 2 |
| 14:10 | 38,6 | 4 | 3 | 3 | 4 | 5 | 2 | 2 | 3 | 99 | 2 |
| 14:20 | 39,0 | 5 | 1 | 2 | 3 | 2 | 1 | 99 | 4 | 99 | 1 |
| 14:25 | 33,2 | 7 | 3 | 2 | 3 | 4 | 3 | 2 | 3 | 99 | 2 |
| 14:35 | 33,4 | 7 | 3 | 7 | 3 | 2 | 2 | 2 | 1 | 99 | 2 |
| 14:40 | 33,2 | 7 | 3 | 2 | 3 | 5 | 3 | 2 | 1 | 99 | 2 |
| 14:45 | 33,6 | 7 | 3 | 3 | 3 | 2 | 1 | 2 | 1 | 99 | 2 |
| 15:50 | 32,2 | 7 | 4 | 3 | 3 | 4 | 2 | 1 | 1 | 99 | 2 |
| 13:35 | 33,4 | 6 | 2 | 3 | 4 | 4 | 1 | 1 | 3 | 1 | 2 |
| 15:00 | 33,8 | 7 | 3 | 3 | 3 | 3 | 1 | 2 | 1 | 99 | 2 |
| 15:05 | 35,5 | 6 | 4 | 3 | 2 | 2 | 2 | 2 | 1 | 99 | 2 |
| 15:10 | 34,2 | 7 | 2 | 2 | 3 | 3 | 2 | 2 | 3 | 99 | 2 |
| 15:20 | 33,1 | 7 | 4 | 3 | 4 | 2 | 1 | 1 | 1 | 99 | 2 |
| 15:30 | 33,1 | 7 | 2 | 3 | 4 | 3 | 1 | 1 | 1 | 99 | 2 |
| 15:30 | 33,1 | 7 | 3 | 2 | 3 | 4 | 1 | 99 | 1 | 99 | 2 |
| 15:45 | 32,4 | 7 | 3 | 3 | 4 | 3 | 4 | 99 | 2 | 99 | 2 |
| 15:55 | 32,6 | 7 | 4 | 2 | 4 | 4 | 4 | 99 | 3 | 99 | 2 |
| 16:00 | 33,2 | 7 | 4 | 3 | 3 | 3 | 2 | 99 | 2 | 99 | 2 |
| 15:26 | 33,5 | 7 | 4 | 3 | 2 | 2 | 3 | 2 | 2 | 3 | 2 |
| 15:31 | 33,1 | 6 | 1 | 4 | 2 | 3 | 3 | 2 | 2 | 1 | 2 |
| 15:37 | 32,7 | 7 | 3 | 2 | 4 | 5 | 2 | 1 | 1 | 1 | 2 |
| 15:37 | 32,7 | 4 | 1 | 2 | 4 | 3 | 1 | 1 | 2 | 1 | 2 |
| 15:41 | 32,7 | 7 | 4 | 4 | 3 | 4 | 2 | 1 | 2 | 1 | 2 |
| 15:51 | 32,2 | 4 | 1 | 5 | 3 | 4 | 4 | 1 | 2 | 1 | 2 |
| 15:53 | 32,2 | 7 | 1 | 4 | 4 | 4 | 4 | 1 | 2 | 1 | 2 |
| 16:06 | 41,0 | 6 | 3 | 3 | 4 | 4 | 3 | 1 | 2 | 2 | 1 |
| 16:11 | 32,7 | 6 | 4 | 1 | 4 | 5 | 1 | 1 | 3 | 3 | 2 |
| 16:13 | 32,7 | 6 | 3 | 4 | 4 | 5 | 1 | 1 | 1 | 3 | 2 |
| 16:00 | 38,3 | 7 | 2 | 4 | 3 | 5 | 4 | 2 | 3 | 99 | 1 |
| 16:05 | 41,0 | 7 | 3 | 2 | 3 | 3 | 2 | 2 | 1 | 99 | 1 |
| 16:10 | 32,7 | 7 | 3 | 2 | 2 | 3 | 3 | 2 | 2 | 99 | 2 |
| 16:15 | 32,4 | 7 | 4 | 3 | 4 | 5 | 3 | 2 | 1 | 99 | 2 |
| 16:20 | 32,4 | 7 | 4 | 2 | 4 | 4 | 1 | 1 | 1 | 99 | 2 |
| 16:30 | 32,7 | 7 | 4 | 2 | 3 | 4 | 2 | 2 | 1 | 99 | 2 |
| 16:40 | 33,0 | 7 | 2 | 5 | 3 | 3 | 1 | 1 | 2 | 99 | 2 |
| 16:45 | 32,7 | 7 | 3 | 3 | 3 | 3 | 2 | 2 | 1 | 99 | 2 |
| 16:50 | 32,3 | 7 | 3 | 2 | 3 | 4 | 4 | 2 | 2 | 99 | 2 |
| 16:55 | 32,8 | 7 | 3 | 2 | 4 | 5 | 2 | 2 | 1 | 99 | 2 |
| 16:23 | 32,4 | 7 | 4 | 4 | 4 | 3 | 1 | 2 | 2 | 1 | 2 |
| 16:23 | 32,4 | 6 | 4 | 4 | 3 | 2 | 1 | 2 | 1 | 1 | 2 |
| 16:29 | 32,9 | 7 | 4 | 2 | 4 | 5 | 3 | 1 | 2 | 2 | 2 |
| 16:33 | 32,7 | 7 | 3 | 3 | 3 | 3 | 1 | 2 | 1 | 3 | 2 |
| 16:43 | 33,0 | 7 | 4 | 3 | 4 | 4 | 1 | 2 | 1 | 2 | 2 |
| 16:43 | 33,0 | 7 | 4 | 3 | 3 | 2 | 1 | 2 | 2 | 2 | 2 |
| 16:46 | 32,7 | 7 | 3 | 1 | 4 | 5 | 2 | 1 | 2 | 1 | 2 |
| 16:50 | 35,2 | 7 | 2 | 4 | 3 | 3 | 1 | 2 | 2 | 1 | 1 |
| 16:30 | 32,7 | 7 | 4 | 2 | 2 | 3 | 2 | 1 | 1 | 3 | 2 |
| **Liberdade Square**  **08th July 2013** | | | | | | | | | | | |
| **Time** | **PET (°C)** | **Thermal**  **Perception** | **Thermal**  **Comfort**  **Evaluation** | **Preference**  **of Thermal**  **Sensation** | **Height** | **Weigh** | **Age** | **Gender** | **Clothing** | **Physical Activity** | **Position (sun/shadow)** |
| 07:07 | 17,3 | 2 | 1 | 4 | 2 | 3 | 2 | 2 | 3 | 2 | 2 |
| 07:13 | 16,6 | 3 | 1 | 5 | 3 | 3 | 3 | 2 | 3 | 2 | 2 |
| 07:17 | 18,9 | 3 | 2 | 5 | 2 | 3 | 3 | 2 | 3 | 1 | 2 |
| 07:22 | 16,8 | 2 | 2 | 4 | 3 | 1 | 2 | 1 | 2 | 1 | 2 |
| 07:27 | 19,0 | 4 | 1 | 4 | 4 | 4 | 1 | 1 | 3 | 2 | 2 |
| 07:38 | 17,5 | 4 | 1 | 4 | 4 | 4 | 3 | 1 | 2 | 2 | 2 |
| 07:43 | 17,5 | 5 | 2 | 3 | 3 | 2 | 1 | 2 | 3 | 2 | 2 |
| 07:53 | 18,5 | 4 | 1 | 4 | 5 | 5 | 2 | 1 | 2 | 1 | 2 |
| 08:05 | 17,1 | 5 | 2 | 4 | 3 | 4 | 4 | 2 | 4 | 1 | 2 |
| 08:08 | 17,1 | 4 | 1 | 4 | 3 | 3 | 1 | 2 | 3 | 2 | 2 |
| 08:18 | 17,6 | 4 | 1 | 2 | 4 | 2 | 1 | 1 | 2 | 1 | 2 |
| 08:22 | 17,4 | 1 | 1 | 6 | 3 | 3 | 2 | 2 | 2 | 2 | 2 |
| 08:36 | 27,5 | 4 | 1 | 4 | 2 | 2 | 3 | 2 | 3 | 2 | 2 |
| 08:41 | 25,5 | 4 | 1 | 4 | 3 | 3 | 1 | 2 | 3 | 2 | 2 |
| 09:03 | 18,4 | 3 | 2 | 4 | 3 | 2 | 1 | 2 | 3 | 2 | 2 |
| 09:06 | 18,3 | 4 | 1 | 4 | 3 | 5 | 1 | 2 | 3 | 2 | 2 |
| 09:10 | 20,4 | 1 | 2 | 5 | 2 | 3 | 1 | 2 | 3 | 2 | 2 |
| 09:15 | 20,0 | 4 | 1 | 4 | 4 | 3 | 4 | 2 | 3 | 2 | 2 |
| 09:21 | 20,0 | 4 | 1 | 4 | 3 | 4 | 2 | 2 | 3 | 2 | 2 |
| 09:35 | 22,6 | 4 | 1 | 5 | 4 | 4 | 4 | 1 | 2 | 1 | 1 |
| 09:47 | 20,3 | 5 | 2 | 3 | 2 | 2 | 3 | 2 | 3 | 1 | 2 |
| 09:51 | 18,1 | 4 | 1 | 4 | 3 | 3 | 3 | 2 | 2 | 1 | 2 |
| 09:55 | 19,6 | 4 | 1 | 4 | 4 | 5 | 4 | 1 | 2 | 2 | 2 |
| 10:04 | 19,0 | 4 | 1 | 4 | 5 | 5 | 3 | 1 | 2 | 1 | 2 |
| 10:18 | 26,1 | 4 | 2 | 3 | 3 | 3 | 1 | 2 | 3 | 2 | 1 |
| 10:22 | 21,3 | 4 | 1 | 4 | 4 | 5 | 1 | 1 | 3 | 1 | 2 |
| 11:01 | 30,2 | 4 | 1 | 4 | 3 | 3 | 4 | 2 | 2 | 3 | 1 |
| 11:01 | 30,2 | 4 | 1 | 4 | 2 | 2 | 1 | 2 | 3 | 3 | 1 |
| 11:08 | 30,5 | 6 | 2 | 2 | 4 | 5 | 4 | 1 | 1 | 2 | 1 |
| 11:13 | 30,9 | 6 | 2 | 3 | 4 | 4 | 4 | 1 | 2 | 2 | 1 |
| 11:22 | 30,2 | 4 | 1 | 4 | 3 | 4 | 2 | 2 | 1 | 1 | 1 |
| 11:29 | 29,2 | 4 | 1 | 4 | 4 | 5 | 2 | 1 | 1 | 3 | 1 |
| 11:42 | 22,5 | 4 | 2 | 3 | 3 | 3 | 2 | 2 | 2 | 2 | 2 |
| 11:46 | 30,4 | 7 | 3 | 3 | 3 | 3 | 3 | 2 | 1 | 2 | 1 |
| 11:50 | 25,5 | 5 | 3 | 4 | 4 | 4 | 1 | 1 | 2 | 1 | 2 |
| 11:54 | 30,6 | 4 | 1 | 4 | 4 | 4 | 1 | 1 | 2 | 1 | 1 |
| 11:56 | 24,8 | 5 | 2 | 3 | 3 | 3 | 3 | 2 | 2 | 1 | 2 |
| 12:01 | 23,7 | 5 | 1 | 4 | 3 | 2 | 3 | 2 | 2 | 1 | 2 |
| 07:08 | 19,4 | 3 | 1 | 1 | 3 | 2 | 1 | 2 | 4 | 3 | 1 |
| 07:14 | 16,6 | 3 | 1 | 4 | 3 | 2 | 1 | 1 | 4 | 3 | 2 |
| 07:20 | 16,8 | 4 | 1 | 4 | 3 | 4 | 2 | 1 | 4 | 3 | 2 |
| 07:24 | 16,8 | 4 | 1 | 4 | 2 | 3 | 3 | 2 | 4 | 3 | 2 |
| 07:30 | 17,8 | 3 | 1 | 2 | 2 | 1 | 2 | 2 | 4 | 3 | 2 |
| 07:34 | 17,8 | 3 | 2 | 5 | 3 | 3 | 2 | 1 | 4 | 3 | 2 |
| 07:38 | 18,8 | 2 | 1 | 5 | 3 | 2 | 1 | 2 | 4 | 1 | 1 |
| 07:41 | 19,3 | 2 | 1 | 1 | 2 | 2 | 3 | 2 | 4 | 3 | 1 |
| 07:48 | 19,5 | 4 | 1 | 4 | 2 | 1 | 2 | 2 | 4 | 3 | 1 |
| 07:52 | 18,5 | 3 | 1 | 6 | 3 | 3 | 4 | 2 | 4 | 3 | 2 |
| 07:59 | 17,6 | 3 | 2 | 5 | 3 | 3 | 4 | 1 | 3 | 1 | 2 |
| 08:01 | 17,7 | 3 | 1 | 4 | 4 | 2 | 1 | 1 | 2 | 3 | 1 |
| 08:06 | 17,1 | 3 | 2 | 1 | 2 | 2 | 1 | 2 | 4 | 2 | 2 |
| 08:12 | 18,2 | 3 | 1 | 4 | 3 | 4 | 2 | 1 | 4 | 2 | 1 |
| 08:16 | 19,0 | 3 | 1 | 4 | 2 | 2 | 1 | 2 | 4 | 1 | 1 |
| 08:20 | 17,4 | 4 | 1 | 4 | 3 | 2 | 1 | 1 | 4 | 1 | 2 |
| 08:32 | 18,9 | 1 | 2 | 1 | 3 | 3 | 2 | 1 | 3 | 2 | 1 |
| 08:42 | 20,0 | 1 | 1 | 1 | 4 | 4 | 4 | 1 | 4 | 2 | 1 |
| 08:48 | 22,3 | 4 | 2 | 2 | 2 | 2 | 2 | 2 | 4 | 2 | 1 |
| 08:52 | 24,9 | 4 | 1 | 4 | 4 | 3 | 1 | 1 | 2 | 2 | 1 |
| 09:00 | 26,3 | 3 | 1 | 3 | 3 | 2 | 2 | 2 | 3 | 2 | 1 |
| 09:04 | 26,3 | 1 | 3 | 2 | 4 | 3 | 2 | 1 | 2 | 2 | 1 |
| 09:10 | 25,0 | 4 | 1 | 1 | 3 | 3 | 3 | 2 | 2 | 2 | 1 |
| 09:13 | 25,0 | 1 | 3 | 6 | 3 | 3 | 3 | 2 | 3 | 2 | 1 |
| 09:20 | 23,9 | 1 | 2 | 1 | 3 | 4 | 4 | 1 | 4 | 2 | 1 |
| 09:32 | 22,7 | 4 | 2 | 4 | 3 | 2 | 2 | 2 | 2 | 1 | 1 |
| 09:36 | 21,2 | 3 | 1 | 4 | 3 | 3 | 3 | 2 | 3 | 1 | 2 |
| 09:39 | 21,2 | 4 | 1 | 4 | 2 | 2 | 2 | 2 | 4 | 2 | 2 |
| 09:42 | 24,1 | 5 | 1 | 1 | 3 | 1 | 1 | 2 | 4 | 2 | 1 |
| 09:48 | 25,0 | 4 | 1 | 1 | 4 | 3 | 3 | 1 | 3 | 2 | 1 |
| 10:00 | 25,9 | 4 | 1 | 4 | 3 | 2 | 2 | 2 | 3 | 2 | 1 |
| 10:05 | 24,1 | 4 | 1 | 4 | 2 | 3 | 3 | 2 | 3 | 2 | 2 |
| 10:09 | 25,8 | 3 | 1 | 5 | 3 | 2 | 1 | 2 | 4 | 2 | 1 |
| 10:13 | 25,4 | 4 | 1 | 1 | 2 | 3 | 4 | 2 | 3 | 2 | 1 |
| 10:19 | 26,1 | 4 | 1 | 4 | 4 | 3 | 4 | 1 | 4 | 1 | 1 |
| 10:25 | 27,6 | 1 | 1 | 1 | 3 | 3 | 1 | 2 | 3 | 2 | 1 |
| 10:30 | 27,8 | 5 | 3 | 1 | 4 | 3 | 2 | 2 | 3 | 2 | 1 |
| 10:35 | 21,8 | 4 | 1 | 1 | 3 | 2 | 2 | 2 | 3 | 2 | 2 |
| 10:41 | 28,2 | 4 | 1 | 1 | 5 | 5 | 4 | 1 | 4 | 2 | 1 |
| 10:50 | 29,6 | 4 | 1 | 1 | 3 | 2 | 1 | 1 | 3 | 2 | 1 |
| 10:49 | 28,6 | 5 | 2 | 5 | 3 | 3 | 4 | 2 | 3 | 2 | 1 |
| 10:57 | 23,1 | 1 | 1 | 1 | 1 | 4 | 3 | 2 | 4 | 2 | 2 |
| 11:17 | 30,7 | 4 | 1 | 1 | 4 | 3 | 1 | 2 | 3 | 2 | 1 |
| 11:20 | 23,0 | 4 | 1 | 5 | 2 | 3 | 3 | 2 | 3 | 1 | 2 |
| 11:26 | 29,2 | 1 | 1 | 2 | 4 | 4 | 4 | 1 | 4 | 1 | 1 |
| 11:29 | 22,6 | 5 | 1 | 4 | 3 | 4 | 3 | 1 | 3 | 2 | 2 |
| 11:36 | 28,3 | 1 | 2 | 4 | 5 | 4 | 4 | 2 | 3 | 2 | 1 |
| 11:42 | 22,5 | 4 | 1 | 5 | 4 | 4 | 4 | 1 | 3 | 1 | 2 |
| 11:45 | 30,4 | 1 | 2 | 4 | 3 | 3 | 3 | 2 | 3 | 2 | 1 |
| 11:51 | 25,5 | 3 | 1 | 5 | 4 | 4 | 3 | 1 | 4 | 2 | 2 |
| 12:10 | 24,5 | 4 | 2 | 5 | 3 | 4 | 2 | 2 | 2 | 2 | 2 |
| 12:14 | 24,5 | 6 | 2 | 4 | 4 | 3 | 3 | 2 | 2 | 1 | 2 |
| 12:18 | 25,1 | 6 | 2 | 4 | 3 | 4 | 2 | 2 | 2 | 2 | 2 |
| 12:27 | 23,2 | 6 | 1 | 3 | 4 | 3 | 2 | 1 | 2 | 1 | 2 |
| 12:31 | 22,9 | 7 | 2 | 1 | 3 | 3 | 1 | 2 | 2 | 1 | 2 |
| 12:35 | 23,2 | 6 | 2 | 4 | 3 | 2 | 2 | 2 | 2 | 1 | 2 |
| 12:40 | 21,9 | 6 | 2 | 3 | 4 | 3 | 1 | 2 | 2 | 1 | 2 |
| 12:44 | 21,9 | 7 | 2 | 1 | 4 | 5 | 1 | 1 | 2 | 2 | 2 |
| 12:57 | 23,5 | 4 | 2 | 3 | 3 | 3 | 1 | 2 | 2 | 1 | 2 |
| 13:00 | 24,5 | 6 | 2 | 2 | 4 | 4 | 2 | 1 | 1 | 1 | 2 |
| 13:17 | 25,4 | 6 | 2 | 2 | 3 | 3 | 2 | 2 | 2 | 2 | 2 |
| 13:23 | 25,1 | 4 | 2 | 4 | 4 | 4 | 4 | 1 | 1 | 2 | 2 |
| 13:30 | 30,9 | 5 | 1 | 4 | 4 | 4 | 2 | 1 | 2 | 1 | 1 |
| 13:50 | 27,0 | 5 | 2 | 3 | 3 | 4 | 2 | 1 | 2 | 3 | 2 |
| 13:54 | 27,0 | 5 | 1 | 3 | 2 | 2 | 2 | 2 | 2 | 2 | 2 |
| 13:54 | 27,0 | 6 | 3 | 3 | 3 | 3 | 2 | 2 | 2 | 2 | 2 |
| 15:18 | 26,6 | 4 | 1 | 4 | 3 | 3 | 1 | 2 | 2 | 2 | 2 |
| 15:23 | 25,2 | 6 | 2 | 2 | 2 | 3 | 1 | 2 | 2 | 2 | 2 |
| 15:26 | 24,1 | 4 | 1 | 4 | 4 | 4 | 3 | 1 | 2 | 2 | 2 |
| 15:29 | 24,1 | 4 | 2 | 3 | 4 | 3 | 1 | 2 | 3 | 1 | 2 |
| 15:34 | 22,4 | 4 | 1 | 4 | 3 | 2 | 3 | 2 | 2 | 2 | 2 |
| 15:38 | 22,8 | 6 | 1 | 4 | 3 | 3 | 4 | 2 | 2 | 1 | 2 |
| 15:48 | 21,9 | 3 | 1 | 4 | 3 | 2 | 3 | 2 | 3 | 2 | 2 |
| 15:55 | 26,8 | 4 | 2 | 3 | 3 | 2 | 2 | 2 | 2 | 1 | 2 |
| 16:00 | 25,4 | 6 | 3 | 2 | 4 | 3 | 1 | 1 | 2 | 2 | 2 |
| 16:05 | 25,3 | 4 | 1 | 4 | 4 | 4 | 4 | 1 | 2 | 2 | 2 |
| 16:10 | 23,7 | 6 | 1 | 4 | 2 | 4 | 2 | 2 | 2 | 2 | 2 |
| 16:15 | 23,0 | 4 | 1 | 4 | 3 | 4 | 4 | 2 | 3 | 1 | 2 |
| 16:30 | 23,9 | 4 | 1 | 4 | 3 | 3 | 2 | 2 | 3 | 2 | 2 |
| 16:34 | 23,9 | 5 | 2 | 4 | 4 | 4 | 3 | 2 | 3 | 2 | 2 |
| 16:44 | 22,4 | 4 | 1 | 4 | 3 | 4 | 3 | 2 | 2 | 1 | 2 |
| 16:44 | 22,4 | 4 | 1 | 5 | 3 | 3 | 3 | 2 | 2 | 1 | 2 |
| 16:47 | 22,7 | 7 | 3 | 4 | 3 | 2 | 1 | 1 | 3 | 2 | 2 |
| 16:57 | 24,4 | 5 | 1 | 4 | 3 | 2 | 2 | 2 | 2 | 2 | 2 |
| 17:00 | 23,3 | 4 | 1 | 5 | 3 | 5 | 3 | 2 | 2 | 1 | 2 |
| 16:01 | 25,4 | 4 | 1 | 4 | 3 | 4 | 2 | 1 | 3 | 1 | 2 |
| 16:08 | 25,3 | 4 | 1 | 4 | 3 | 3 | 1 | 2 | 2 | 1 | 2 |
| 16:12 | 23,7 | 3 | 1 | 4 | 3 | 1 | 3 | 2 | 1 | 1 | 2 |
| 16:18 | 23,0 | 4 | 1 | 4 | 4 | 3 | 2 | 1 | 2 | 1 | 2 |
| 16:22 | 23,8 | 4 | 4 | 2 | 5 | 4 | 1 | 1 | 2 | 1 | 2 |
| 16:36 | 22,2 | 3 | 1 | 4 | 5 | 4 | 3 | 1 | 3 | 1 | 2 |
| 16:40 | 22,4 | 4 | 1 | 4 | 4 | 4 | 4 | 1 | 2 | 1 | 2 |
| 15:08 | 26,3 | 4 | 1 | 3 | 4 | 4 | 1 | 1 | 2 | 3 | 2 |
| 15:12 | 26,6 | 4 | 1 | 4 | 2 | 3 | 4 | 2 | 3 | 1 | 2 |
| 15:15 | 26,6 | 4 | 1 | 4 | 3 | 2 | 1 | 2 | 1 | 1 | 2 |
| 15:18 | 26,6 | 4 | 1 | 4 | 4 | 1 | 1 | 2 | 2 | 1 | 2 |
| 15:20 | 25,2 | 4 | 1 | 4 | 2 | 2 | 4 | 2 | 2 | 1 | 2 |
| 15:25 | 24,1 | 4 | 1 | 4 | 4 | 3 | 1 | 1 | 2 | 1 | 2 |
| 15:28 | 24,1 | 4 | 1 | 2 | 4 | 3 | 1 | 1 | 2 | 1 | 2 |
| 15:35 | 22,8 | 2 | 1 | 6 | 2 | 1 | 1 | 2 | 3 | 1 | 2 |
| 15:38 | 22,8 | 1 | 1 | 6 | 3 | 2 | 3 | 2 | 2 | 1 | 2 |
| 15:49 | 21,9 | 4 | 1 | 2 | 3 | 3 | 2 | 2 | 99 | 1 | 2 |
| 14:18 | 30,8 | 4 | 1 | 4 | 2 | 2 | 4 | 2 | 3 | 1 | 1 |
| 14:24 | 31,3 | 6 | 2 | 2 | 5 | 5 | 1 | 1 | 3 | 1 | 1 |
| 14:34 | 26,2 | 4 | 1 | 4 | 4 | 5 | 3 | 1 | 3 | 2 | 2 |
| 14:38 | 25,6 | 4 | 2 | 4 | 5 | 5 | 3 | 1 | 2 | 1 | 2 |
| 14:42 | 30,2 | 4 | 2 | 2 | 3 | 3 | 2 | 2 | 2 | 1 | 1 |
| 14:48 | 26,0 | 4 | 1 | 2 | 4 | 4 | 3 | 1 | 3 | 3 | 2 |
| 14:49 | 26,0 | 4 | 1 | 4 | 3 | 1 | 1 | 2 | 2 | 1 | 2 |
| 14:58 | 26,0 | 4 | 1 | 2 | 2 | 4 | 1 | 1 | 3 | 1 | 2 |
| 15:01 | 26,7 | 4 | 2 | 4 | 4 | 2 | 2 | 2 | 2 | 2 | 2 |
| 15:05 | 26,3 | 7 | 2 | 4 | 3 | 2 | 3 | 2 | 3 | 3 | 2 |
| 13:40 | 30,7 | 4 | 1 | 4 | 4 | 3 | 1 | 1 | 2 | 1 | 1 |
| 13:45 | 29,5 | 4 | 1 | 2 | 4 | 3 | 2 | 1 | 3 | 2 | 2 |
| 13:52 | 31,6 | 4 | 2 | 4 | 4 | 3 | 1 | 1 | 2 | 1 | 1 |
| 13:54 | 31,6 | 4 | 1 | 4 | 5 | 4 | 1 | 1 | 2 | 1 | 1 |
| 13:56 | 31,8 | 4 | 1 | 4 | 3 | 4 | 1 | 2 | 2 | 1 | 1 |
| 13:59 | 31,8 | 4 | 1 | 4 | 5 | 4 | 1 | 2 | 3 | 1 | 1 |
| 14:02 | 24,6 | 4 | 1 | 4 | 4 | 3 | 1 | 1 | 3 | 1 | 2 |
| 14:05 | 24,9 | 4 | 1 | 4 | 2 | 3 | 1 | 2 | 3 | 1 | 2 |
| 14:10 | 31,2 | 6 | 1 | 4 | 4 | 3 | 1 | 2 | 3 | 1 | 1 |
| 14:12 | 24,6 | 4 | 1 | 4 | 4 | 5 | 3 | 1 | 99 | 1 | 2 |
| 13:02 | 24,5 | 6 | 1 | 4 | 3 | 3 | 1 | 1 | 2 | 1 | 2 |
| 13:05 | 30,9 | 2 | 2 | 5 | 4 | 2 | 3 | 2 | 3 | 1 | 1 |
| 13:10 | 23,1 | 4 | 1 | 4 | 4 | 4 | 1 | 2 | 2 | 1 | 2 |
| 13:18 | 25,4 | 4 | 1 | 5 | 5 | 4 | 1 | 1 | 3 | 1 | 2 |
| 13:20 | 25,1 | 2 | 2 | 4 | 3 | 3 | 1 | 2 | 2 | 1 | 2 |
| 13:25 | 26,3 | 1 | 1 | 4 | 3 | 2 | 1 | 2 | 2 | 1 | 2 |
| 13:27 | 26,3 | 4 | 1 | 4 | 4 | 2 | 1 | 1 | 2 | 1 | 2 |
| 13:29 | 26,3 | 4 | 1 | 4 | 3 | 1 | 1 | 1 | 3 | 1 | 2 |
| 13:32 | 25,8 | 4 | 1 | 4 | 5 | 4 | 1 | 1 | 1 | 1 | 2 |
| 13:36 | 30,4 | 2 | 1 | 6 | 4 | 3 | 99 | 1 | 2 | 1 | 1 |
| 12:13 | 30,6 | 4 | 1 | 4 | 2 | 3 | 2 | 1 | 4 | 1 | 1 |
| 12:22 | 23,5 | 2 | 2 | 5 | 2 | 2 | 2 | 1 | 4 | 1 | 2 |
| 12:28 | 23,2 | 2 | 2 | 5 | 2 | 1 | 2 | 2 | 3 | 1 | 2 |
| 12:32 | 22,9 | 6 | 1 | 3 | 2 | 4 | 2 | 2 | 4 | 1 | 2 |
| 12:39 | 23,2 | 4 | 1 | 3 | 4 | 5 | 1 | 1 | 2 | 1 | 2 |
| 12:42 | 21,9 | 3 | 2 | 3 | 4 | 4 | 2 | 1 | 2 | 1 | 2 |
| 12:46 | 29,7 | 5 | 2 | 3 | 4 | 5 | 1 | 1 | 2 | 1 | 1 |
| 12:49 | 29,7 | 4 | 1 | 4 | 5 | 5 | 1 | 1 | 2 | 2 | 1 |
| 12:51 | 29,9 | 4 | 1 | 4 | 4 | 4 | 4 | 1 | 2 | 2 | 1 |
| 12:55 | 30,0 | 4 | 1 | 2 | 4 | 4 | 2 | 2 | 2 | 1 | 1 |
| 07:15 | 18,9 | 3 | 1 | 4 | 3 | 4 | 2 | 2 | 3 | 3 | 2 |
| 07:20 | 16,8 | 4 | 1 | 4 | 1 | 2 | 3 | 1 | 2 | 3 | 2 |
| 07:20 | 16,8 | 4 | 1 | 4 | 3 | 4 | 3 | 1 | 2 | 3 | 2 |
| 07:26 | 19,0 | 4 | 1 | 4 | 1 | 1 | 2 | 2 | 3 | 3 | 2 |
| 07:33 | 17,8 | 3 | 1 | 5 | 4 | 4 | 4 | 1 | 2 | 3 | 2 |
| 07:39 | 17,5 | 3 | 1 | 5 | 1 | 4 | 3 | 2 | 2 | 3 | 2 |
| 07:39 | 17,5 | 3 | 1 | 5 | 5 | 4 | 2 | 1 | 2 | 3 | 2 |
| 07:43 | 19,3 | 4 | 1 | 4 | 1 | 2 | 2 | 2 | 3 | 3 | 1 |
| 13:25 | 26,3 | 4 | 1 | 4 | 1 | 3 | 1 | 2 | 2 | 2 | 2 |
| 16:00 | 25,4 | 4 | 1 | 4 | 5 | 4 | 1 | 1 | 1 | 1 | 2 |
| 16:09 | 22,4 | 5 | 3 | 3 | 1 | 1 | 1 | 2 | 2 | 3 | 1 |
| 16:17 | 23,7 | 5 | 4 | 3 | 3 | 3 | 2 | 2 | 2 | 3 | 1 |
| 16:20 | 23,8 | 4 | 1 | 2 | 3 | 4 | 2 | 1 | 2 | 2 | 2 |
| 16:24 | 23,8 | 4 | 1 | 4 | 3 | 4 | 2 | 1 | 2 | 2 | 2 |
| 08:00 | 17,4 | 3 | 1 | 6 | 3 | 2 | 2 | 2 | 1 | 1 | 2 |
| 08:05 | 17,1 | 2 | 1 | 6 | 4 | 2 | 1 | 1 | 3 | 1 | 2 |
| 08:09 | 17,1 | 4 | 2 | 4 | 3 | 3 | 4 | 2 | 3 | 1 | 2 |
| 08:13 | 17,0 | 4 | 1 | 4 | 4 | 4 | 5 | 1 | 3 | 3 | 2 |
| 08:18 | 17,6 | 4 | 1 | 4 | 4 | 4 | 3 | 1 | 3 | 3 | 2 |
| 08:23 | 17,4 | 4 | 1 | 4 | 4 | 4 | 1 | 1 | 3 | 3 | 2 |
| 08:30 | 22,3 | 4 | 1 | 5 | 4 | 2 | 1 | 2 | 2 | 3 | 2 |
| 08:35 | 27,5 | 4 | 1 | 4 | 3 | 2 | 1 | 2 | 3 | 3 | 2 |
| 08:39 | 27,5 | 4 | 1 | 2 | 5 | 3 | 5 | 1 | 3 | 3 | 2 |
| 08:47 | 22,9 | 4 | 1 | 5 | 3 | 4 | 2 | 2 | 3 | 3 | 2 |
| 08:56 | 19,6 | 4 | 1 | 3 | 5 | 3 | 5 | 1 | 3 | 3 | 2 |
| 09:00 | 26,3 | 3 | 2 | 6 | 3 | 3 | 3 | 2 | 3 | 1 | 1 |
| 09:05 | 18,3 | 2 | 2 | 6 | 3 | 4 | 2 | 1 | 3 | 1 | 2 |
| 09:09 | 18,3 | 4 | 1 | 4 | 3 | 2 | 1 | 2 | 3 | 1 | 2 |
| 09:13 | 20,4 | 4 | 1 | 4 | 3 | 4 | 4 | 1 | 3 | 2 | 2 |
| 09:20 | 23,9 | 4 | 1 | 4 | 2 | 1 | 2 | 2 | 3 | 1 | 1 |
| 09:26 | 18,8 | 4 | 2 | 5 | 3 | 3 | 1 | 99 | 1 | 1 | 2 |
| 09:31 | 22,7 | 3 | 1 | 4 | 3 | 3 | 1 | 2 | 3 | 2 | 1 |
| 09:40 | 20,9 | 4 | 1 | 4 | 4 | 4 | 5 | 1 | 3 | 3 | 2 |
| 09:45 | 20,3 | 4 | 1 | 4 | 4 | 4 | 1 | 1 | 2 | 1 | 2 |
| 09:57 | 26,1 | 4 | 1 | 4 | 1 | 1 | 5 | 1 | 3 | 1 | 1 |
| 10:04 | 19,0 | 4 | 1 | 4 | 3 | 4 | 2 | 1 | 2 | 3 | 2 |
| 10:09 | 24,1 | 4 | 1 | 4 | 3 | 3 | 3 | 2 | 3 | 3 | 2 |
| 10:15 | 26,1 | 4 | 1 | 2 | 3 | 4 | 1 | 2 | 2 | 1 | 1 |
| 10:19 | 23,1 | 2 | 1 | 4 | 4 | 4 | 2 | 1 | 3 | 1 | 2 |
| 10:30 | 27,8 | 6 | 1 | 5 | 3 | 3 | 3 | 2 | 1 | 2 | 1 |
| 10:36 | 21,8 | 6 | 1 | 4 | 3 | 3 | 2 | 2 | 1 | 1 | 2 |
| 10:43 | 20,8 | 2 | 1 | 2 | 4 | 1 | 3 | 2 | 3 | 1 | 2 |
| 10:47 | 22,1 | 4 | 1 | 6 | 4 | 1 | 4 | 1 | 2 | 1 | 2 |
| 10:51 | 24,2 | 4 | 1 | 4 | 4 | 4 | 1 | 1 | 2 | 1 | 2 |
| 10:57 | 23,1 | 4 | 1 | 4 | 4 | 3 | 5 | 1 | 2 | 2 | 2 |
| 11:05 | 30,5 | 4 | 1 | 4 | 4 | 3 | 2 | 2 | 2 | 3 | 1 |
| 11:07 | 30,5 | 4 | 1 | 4 | 4 | 5 | 4 | 1 | 2 | 3 | 1 |
| 11:12 | 22,0 | 4 | 1 | 3 | 2 | 3 | 2 | 2 | 2 | 1 | 2 |
| 11:16 | 22,9 | 4 | 1 | 4 | 3 | 3 | 2 | 1 | 3 | 1 | 2 |
| 11:20 | 23,0 | 4 | 1 | 4 | 3 | 3 | 2 | 1 | 2 | 1 | 2 |
| 11:26 | 29,2 | 4 | 1 | 2 | 3 | 2 | 1 | 2 | 3 | 1 | 1 |
| 11:30 | 22,3 | 4 | 1 | 5 | 3 | 3 | 1 | 2 | 3 | 1 | 2 |
| 11:39 | 22,3 | 4 | 2 | 3 | 3 | 3 | 1 | 2 | 3 | 1 | 2 |
| 11:46 | 24,8 | 2 | 1 | 4 | 3 | 3 | 5 | 1 | 3 | 1 | 2 |
| 11:53 | 25,5 | 4 | 1 | 4 | 2 | 3 | 1 | 2 | 1 | 1 | 2 |
| 07:05 | 17,3 | 1 | 2 | 1 | 2 | 2 | 3 | 2 | 3 | 3 | 2 |
| 07:08 | 17,3 | 4 | 1 | 1 | 2 | 4 | 4 | 2 | 3 | 1 | 2 |
| 07:15 | 18,9 | 4 | 2 | 6 | 3 | 3 | 1 | 1 | 3 | 3 | 2 |
| 07:27 | 19,0 | 4 | 1 | 4 | 5 | 2 | 5 | 1 | 3 | 1 | 2 |
| 07:34 | 17,8 | 2 | 1 | 6 | 3 | 3 | 2 | 2 | 3 | 1 | 2 |
| 07:37 | 17,5 | 2 | 2 | 3 | 3 | 2 | 3 | 2 | 3 | 1 | 2 |
| 07:45 | 18,8 | 2 | 1 | 4 | 2 | 3 | 3 | 2 | 1 | 3 | 2 |
| 07:50 | 18,5 | 2 | 2 | 5 | 3 | 3 | 3 | 2 | 2 | 3 | 2 |
| 07:53 | 18,5 | 4 | 1 | 4 | 5 | 3 | 5 | 1 | 3 | 3 | 2 |
| 07:58 | 17,6 | 4 | 2 | 4 | 3 | 3 | 4 | 2 | 3 | 3 | 2 |
| 07:05 | 17,3 | 1 | 1 | 6 | 3 | 2 | 1 | 1 | 4 | 3 | 2 |
| 07:10 | 16,6 | 5 | 1 | 2 | 2 | 2 | 1 | 2 | 4 | 3 | 2 |
| 07:15 | 18,9 | 4 | 1 | 6 | 3 | 2 | 1 | 1 | 4 | 3 | 2 |
| 07:19 | 18,9 | 4 | 1 | 5 | 4 | 3 | 4 | 1 | 4 | 3 | 2 |
| 07:25 | 19,0 | 4 | 1 | 4 | 3 | 3 | 1 | 1 | 2 | 1 | 2 |
| 07:33 | 17,8 | 4 | 1 | 4 | 3 | 2 | 4 | 1 | 4 | 3 | 2 |
| 07:38 | 17,5 | 4 | 1 | 2 | 3 | 2 | 4 | 1 | 4 | 3 | 2 |
| 07:42 | 17,5 | 2 | 1 | 4 | 2 | 4 | 1 | 2 | 4 | 3 | 2 |
| 07:44 | 19,3 | 4 | 1 | 4 | 2 | 1 | 1 | 2 | 3 | 1 | 1 |
| 07:46 | 19,5 | 4 | 1 | 4 | 4 | 3 | 1 | 1 | 4 | 1 | 1 |
| 07:52 | 19,9 | 4 | 1 | 4 | 2 | 2 | 1 | 2 | 3 | 1 | 1 |
| 07:57 | 19,9 | 4 | 1 | 4 | 3 | 4 | 4 | 1 | 4 | 1 | 1 |
| 08:04 | 17,4 | 4 | 1 | 4 | 1 | 1 | 2 | 2 | 3 | 3 | 2 |
| 08:12 | 17,0 | 7 | 2 | 3 | 3 | 4 | 2 | 2 | 2 | 1 | 2 |
| 08:20 | 17,4 | 4 | 2 | 4 | 3 | 2 | 1 | 1 | 2 | 3 | 2 |
| 08:30 | 22,3 | 4 | 2 | 4 | 1 | 2 | 4 | 2 | 3 | 3 | 2 |
| 08:33 | 22,3 | 4 | 1 | 4 | 2 | 2 | 3 | 2 | 2 | 3 | 2 |
| 08:36 | 27,5 | 4 | 1 | 4 | 2 | 2 | 4 | 1 | 1 | 3 | 2 |
| 08:40 | 25,5 | 2 | 3 | 6 | 3 | 4 | 3 | 2 | 3 | 3 | 2 |
| 08:48 | 22,3 | 4 | 1 | 4 | 1 | 3 | 4 | 2 | 2 | 3 | 1 |
| 08:51 | 24,9 | 6 | 1 | 5 | 2 | 1 | 4 | 2 | 2 | 1 | 1 |
| 08:55 | 26,3 | 4 | 1 | 4 | 2 | 3 | 4 | 1 | 1 | 3 | 1 |
| 08:59 | 26,3 | 6 | 1 | 4 | 3 | 4 | 2 | 1 | 2 | 3 | 1 |
| 09:04 | 26,3 | 2 | 1 | 4 | 2 | 1 | 1 | 2 | 3 | 1 | 1 |
| 09:11 | 20,4 | 4 | 1 | 4 | 4 | 4 | 4 | 1 | 2 | 1 | 2 |
| 09:22 | 23,9 | 4 | 1 | 3 | 2 | 1 | 1 | 2 | 3 | 1 | 1 |
| 09:25 | 18,8 | 4 | 1 | 4 | 2 | 1 | 3 | 2 | 3 | 2 | 2 |
| 09:28 | 18,8 | 5 | 1 | 5 | 3 | 1 | 1 | 2 | 3 | 2 | 2 |
| 09:38 | 22,6 | 4 | 1 | 4 | 3 | 3 | 1 | 1 | 2 | 1 | 1 |
| 09:43 | 20,9 | 4 | 1 | 4 | 2 | 2 | 2 | 2 | 3 | 1 | 2 |
| 09:50 | 18,1 | 4 | 1 | 4 | 2 | 1 | 1 | 2 | 2 | 1 | 2 |
| 09:52 | 18,1 | 4 | 1 | 4 | 3 | 3 | 2 | 1 | 2 | 1 | 2 |
| 09:55 | 19,6 | 4 | 1 | 4 | 4 | 5 | 4 | 1 | 3 | 2 | 2 |
| 09:58 | 19,6 | 4 | 1 | 4 | 2 | 3 | 2 | 1 | 2 | 1 | 2 |
| 10:10 | 24,9 | 4 | 1 | 2 | 3 | 2 | 3 | 1 | 3 | 1 | 2 |
| 10:15 | 23,1 | 4 | 1 | 2 | 3 | 1 | 2 | 2 | 3 | 1 | 2 |
| 10:20 | 21,3 | 4 | 1 | 2 | 3 | 1 | 2 | 2 | 3 | 1 | 2 |
| 10:23 | 26,4 | 3 | 1 | 4 | 3 | 4 | 4 | 1 | 4 | 1 | 1 |
| 10:26 | 27,6 | 4 | 1 | 1 | 2 | 1 | 3 | 2 | 2 | 2 | 1 |
| 10:31 | 27,8 | 4 | 1 | 4 | 3 | 1 | 2 | 2 | 2 | 3 | 1 |
| 10:36 | 29,3 | 6 | 1 | 4 | 1 | 3 | 4 | 2 | 3 | 3 | 1 |
| 10:42 | 20,8 | 4 | 1 | 3 | 4 | 5 | 2 | 1 | 3 | 1 | 2 |
| 10:43 | 20,8 | 4 | 1 | 4 | 3 | 1 | 1 | 2 | 2 | 1 | 2 |
| 10:46 | 22,1 | 3 | 2 | 2 | 4 | 3 | 4 | 1 | 2 | 1 | 2 |
| 10:51 | 24,2 | 4 | 1 | 4 | 4 | 3 | 2 | 1 | 3 | 3 | 2 |
| 10:54 | 24,2 | 4 | 1 | 4 | 3 | 2 | 3 | 1 | 2 | 1 | 2 |
| 11:04 | 30,2 | 4 | 1 | 4 | 4 | 5 | 3 | 1 | 2 | 1 | 1 |
| 11:15 | 22,9 | 4 | 1 | 4 | 4 | 4 | 4 | 1 | 2 | 3 | 2 |
| 11:22 | 23,0 | 4 | 1 | 4 | 4 | 3 | 2 | 1 | 1 | 5 | 2 |
| 11:27 | 22,6 | 4 | 1 | 4 | 1 | 1 | 4 | 2 | 3 | 1 | 2 |
| 16:03 | 25,4 | 4 | 1 | 2 | 3 | 3 | 1 | 2 | 3 | 1 | 2 |
| 16:07 | 25,3 | 4 | 1 | 4 | 5 | 5 | 2 | 1 | 2 | 3 | 2 |
| 16:12 | 23,7 | 4 | 1 | 4 | 3 | 3 | 2 | 2 | 3 | 1 | 2 |
| 16:17 | 23,0 | 4 | 1 | 4 | 2 | 2 | 2 | 99 | 3 | 99 | 2 |
| 16:23 | 23,8 | 4 | 1 | 4 | 4 | 2 | 4 | 2 | 3 | 3 | 2 |
| 16:28 | 23,7 | 4 | 1 | 5 | 2 | 4 | 3 | 2 | 3 | 1 | 2 |
| 16:34 | 23,9 | 5 | 2 | 3 | 3 | 4 | 1 | 2 | 3 | 3 | 2 |
| 16:40 | 22,4 | 4 | 1 | 4 | 3 | 4 | 1 | 1 | 1 | 1 | 2 |
| 16:47 | 22,7 | 4 | 1 | 4 | 5 | 4 | 1 | 1 | 2 | 1 | 2 |
| 16:53 | 24,1 | 2 | 1 | 4 | 5 | 4 | 2 | 1 | 3 | 3 | 2 |
| 15:02 | 30,3 | 4 | 1 | 4 | 4 | 4 | 1 | 1 | 3 | 1 | 1 |
| 15:08 | 26,3 | 4 | 1 | 2 | 5 | 3 | 1 | 1 | 1 | 1 | 2 |
| 15:14 | 26,6 | 4 | 1 | 4 | 2 | 2 | 1 | 2 | 1 | 1 | 2 |
| 15:21 | 25,2 | 4 | 1 | 4 | 4 | 2 | 4 | 2 | 3 | 1 | 2 |
| 15:29 | 24,1 | 4 | 1 | 4 | 1 | 1 | 4 | 2 | 3 | 1 | 2 |
| 15:32 | 22,4 | 4 | 1 | 4 | 4 | 3 | 4 | 1 | 3 | 1 | 2 |
| 15:37 | 22,8 | 5 | 2 | 3 | 4 | 5 | 2 | 1 | 3 | 3 | 1 |
| 15:41 | 21,9 | 4 | 1 | 4 | 4 | 3 | 4 | 2 | 3 | 3 | 2 |
| 15:47 | 21,9 | 4 | 1 | 4 | 2 | 3 | 4 | 2 | 3 | 1 | 2 |
| 15:56 | 21,7 | 4 | 1 | 4 | 3 | 2 | 4 | 1 | 3 | 3 | 1 |
| 14:00 | 30,7 | 4 | 1 | 4 | 4 | 4 | 2 | 1 | 3 | 2 | 1 |
| 14:07 | 24,9 | 4 | 1 | 2 | 4 | 1 | 1 | 1 | 3 | 2 | 2 |
| 14:11 | 31,2 | 4 | 1 | 4 | 4 | 3 | 4 | 2 | 3 | 2 | 1 |
| 14:15 | 25,2 | 4 | 1 | 2 | 4 | 4 | 4 | 2 | 3 | 1 | 2 |
| 14:19 | 30,8 | 4 | 1 | 4 | 2 | 5 | 4 | 1 | 1 | 1 | 1 |
| 14:24 | 25,1 | 4 | 1 | 3 | 5 | 2 | 5 | 1 | 2 | 1 | 2 |
| 14:29 | 27,2 | 4 | 1 | 4 | 2 | 2 | 1 | 2 | 2 | 1 | 2 |
| 14:33 | 31,3 | 4 | 1 | 4 | 4 | 5 | 4 | 1 | 2 | 3 | 1 |
| 14:40 | 30,2 | 2 | 1 | 6 | 3 | 3 | 4 | 2 | 3 | 1 | 1 |
| 14:55 | 31,5 | 6 | 2 | 3 | 2 | 2 | 1 | 2 | 2 | 1 | 1 |
| 13:05 | 30,9 | 4 | 1 | 4 | 2 | 3 | 1 | 2 | 2 | 3 | 1 |
| 13:09 | 30,9 | 4 | 1 | 4 | 4 | 4 | 4 | 1 | 2 | 1 | 1 |
| 13:15 | 30,7 | 4 | 1 | 4 | 3 | 3 | 2 | 2 | 2 | 3 | 1 |
| 13:19 | 25,4 | 4 | 1 | 5 | 4 | 5 | 3 | 1 | 2 | 1 | 2 |
| 13:22 | 30,9 | 4 | 1 | 4 | 3 | 2 | 1 | 2 | 3 | 1 | 1 |
| 13:30 | 30,9 | 4 | 1 | 4 | 3 | 1 | 2 | 2 | 3 | 1 | 1 |
| 13:35 | 30,4 | 4 | 1 | 3 | 5 | 4 | 1 | 1 | 2 | 1 | 1 |
| 13:40 | 28,1 | 4 | 1 | 4 | 5 | 5 | 2 | 1 | 2 | 1 | 2 |
| 13:46 | 32,1 | 4 | 1 | 4 | 3 | 3 | 3 | 2 | 3 | 3 | 1 |
| 13:52 | 27,0 | 4 | 1 | 3 | 3 | 2 | 2 | 99 | 4 | 99 | 2 |
| 12:03 | 23,7 | 4 | 2 | 4 | 4 | 4 | 1 | 2 | 3 | 1 | 2 |
| 12:09 | 23,4 | 4 | 2 | 4 | 2 | 2 | 1 | 2 | 3 | 1 | 2 |
| 12:14 | 24,5 | 4 | 1 | 4 | 4 | 3 | 5 | 1 | 2 | 1 | 2 |
| 12:20 | 23,5 | 4 | 1 | 4 | 3 | 1 | 4 | 1 | 1 | 1 | 2 |
| 12:25 | 23,2 | 4 | 1 | 2 | 5 | 1 | 4 | 1 | 3 | 1 | 2 |
| 12:31 | 22,9 | 4 | 1 | 4 | 3 | 3 | 1 | 2 | 2 | 1 | 2 |
| 12:38 | 23,2 | 4 | 1 | 2 | 2 | 2 | 1 | 2 | 2 | 1 | 2 |
| 12:42 | 21,9 | 4 | 1 | 6 | 1 | 3 | 4 | 2 | 2 | 1 | 2 |
| 12:47 | 23,4 | 4 | 1 | 4 | 3 | 3 | 1 | 2 | 2 | 1 | 2 |
| 12:58 | 23,5 | 4 | 1 | 4 | 4 | 5 | 1 | 1 | 3 | 1 | 2 |
| 16:05 | 25,3 | 4 | 1 | 4 | 3 | 4 | 4 | 1 | 2 | 1 | 2 |
| 16:08 | 25,3 | 1 | 1 | 4 | 2 | 3 | 1 | 2 | 2 | 2 | 2 |
| 16:11 | 23,7 | 4 | 1 | 7 | 2 | 3 | 3 | 2 | 3 | 1 | 2 |
| 16:16 | 23,0 | 4 | 1 | 4 | 3 | 4 | 3 | 2 | 2 | 1 | 2 |
| 16:22 | 23,8 | 2 | 1 | 4 | 3 | 5 | 3 | 1 | 2 | 99 | 2 |
| 16:26 | 23,7 | 4 | 2 | 7 | 2 | 3 | 2 | 2 | 3 | 99 | 2 |
| 16:34 | 23,9 | 3 | 1 | 4 | 2 | 2 | 1 | 2 | 3 | 1 | 2 |
| 16:40 | 22,4 | 3 | 1 | 5 | 1 | 2 | 2 | 1 | 3 | 1 | 2 |
| 16:45 | 22,7 | 4 | 1 | 7 | 3 | 2 | 1 | 2 | 2 | 1 | 2 |
| 16:49 | 22,7 | 2 | 1 | 4 | 2 | 3 | 3 | 2 | 2 | 1 | 2 |
| 15:02 | 30,3 | 4 | 1 | 4 | 3 | 2 | 1 | 1 | 2 | 1 | 1 |
| 15:10 | 26,6 | 4 | 1 | 5 | 3 | 3 | 2 | 1 | 2 | 1 | 2 |
| 15:12 | 28,5 | 4 | 1 | 1 | 2 | 2 | 2 | 2 | 2 | 1 | 1 |
| 15:16 | 26,6 | 5 | 1 | 4 | 3 | 3 | 3 | 2 | 2 | 1 | 2 |
| 15:20 | 25,2 | 6 | 1 | 5 | 4 | 3 | 4 | 1 | 3 | 1 | 2 |
| 15:25 | 24,1 | 4 | 2 | 4 | 3 | 3 | 4 | 2 | 2 | 1 | 2 |
| 15:40 | 21,9 | 4 | 1 | 4 | 4 | 4 | 1 | 1 | 2 | 1 | 2 |
| 15:44 | 21,6 | 4 | 1 | 4 | 3 | 2 | 1 | 2 | 3 | 2 | 1 |
| 15:50 | 24,4 | 5 | 1 | 1 | 3 | 3 | 1 | 2 | 2 | 1 | 2 |
| 15:57 | 26,8 | 4 | 2 | 4 | 2 | 2 | 2 | 1 | 2 | 2 | 2 |
| 13:55 | 25,8 | 4 | 1 | 7 | 4 | 4 | 2 | 1 | 4 | 1 | 2 |
| 14:03 | 30,7 | 4 | 1 | 7 | 2 | 3 | 1 | 1 | 2 | 2 | 1 |
| 14:18 | 25,2 | 3 | 1 | 4 | 1 | 1 | 1 | 2 | 2 | 1 | 2 |
| 14:23 | 25,1 | 3 | 2 | 4 | 2 | 3 | 1 | 2 | 2 | 2 | 2 |
| 14:30 | 31,3 | 6 | 1 | 4 | 3 | 2 | 1 | 1 | 2 | 2 | 1 |
| 14:38 | 25,6 | 4 | 1 | 4 | 5 | 5 | 1 | 1 | 2 | 2 | 2 |
| 14:42 | 25,9 | 4 | 1 | 4 | 4 | 5 | 2 | 1 | 2 | 2 | 2 |
| 14:47 | 26,0 | 4 | 1 | 4 | 4 | 4 | 1 | 1 | 2 | 2 | 2 |
| 14:50 | 26,2 | 4 | 1 | 4 | 3 | 4 | 3 | 1 | 2 | 1 | 2 |
| 14:59 | 26,0 | 5 | 1 | 7 | 2 | 3 | 2 | 2 | 3 | 1 | 2 |
| 12:10 | 30,6 | 4 | 1 | 4 | 2 | 2 | 2 | 2 | 2 | 1 | 1 |
| 12:17 | 25,1 | 2 | 1 | 1 | 2 | 2 | 1 | 2 | 3 | 1 | 2 |
| 12:23 | 23,5 | 4 | 1 | 4 | 2 | 1 | 1 | 2 | 2 | 2 | 2 |
| 12:34 | 22,9 | 6 | 3 | 1 | 3 | 2 | 1 | 2 | 1 | 1 | 2 |
| 12:46 | 23,4 | 2 | 4 | 4 | 4 | 3 | 1 | 1 | 1 | 1 | 2 |
| 13:13 | 30,6 | 6 | 2 | 3 | 3 | 4 | 2 | 1 | 2 | 2 | 1 |
| 13:30 | 25,8 | 4 | 1 | 4 | 4 | 4 | 2 | 1 | 3 | 1 | 2 |
| 13:39 | 30,4 | 4 | 1 | 4 | 3 | 3 | 2 | 1 | 2 | 1 | 1 |
| 13:47 | 29,5 | 3 | 1 | 4 | 2 | 2 | 2 | 2 | 2 | 1 | 2 |
| 13:51 | 27,0 | 3 | 1 | 4 | 2 | 3 | 2 | 2 | 3 | 1 | 2 |
| 11:33 | 28,8 | 4 | 1 | 4 | 2 | 1 | 1 | 2 | 2 | 1 | 1 |
| 11:37 | 28,3 | 4 | 1 | 2 | 2 | 3 | 1 | 1 | 2 | 1 | 1 |
| 11:40 | 22,5 | 4 | 1 | 4 | 3 | 4 | 1 | 2 | 2 | 1 | 2 |
| 11:47 | 24,8 | 4 | 2 | 4 | 1 | 1 | 2 | 2 | 2 | 1 | 2 |
| **Sete de Setembro Square**  **09th July 2013** | | | | | | | | | | | |
| **Time** | **PET (°C)** | **Thermal**  **Perception** | **Thermal**  **Comfort**  **Evaluation** | **Preference**  **of Thermal**  **Sensation** | **Height** | **Weigh** | **Age** | **Gender** | **Clothing** | **Physical Activity** | **Position (sun/shadow)** |
| 07:02 | 16,9 | 1 | 2 | 5 | 2 | 3 | 3 | 1 | 4 | 2 | 2 |
| 07:06 | 16,2 | 4 | 1 | 4 | 4 | 3 | 2 | 1 | 3 | 3 | 2 |
| 07:09 | 16,2 | 4 | 1 | 4 | 3 | 3 | 2 | 1 | 2 | 3 | 2 |
| 07:15 | 17,4 | 3 | 3 | 7 | 4 | 5 | 3 | 1 | 1 | 3 | 2 |
| 07:19 | 17,4 | 4 | 1 | 3 | 3 | 4 | 3 | 1 | 4 | 3 | 2 |
| 07:23 | 16,5 | 4 | 2 | 5 | 1 | 1 | 1 | 2 | 3 | 2 | 2 |
| 07:28 | 16,6 | 3 | 2 | 5 | 2 | 2 | 3 | 2 | 3 | 3 | 2 |
| 07:33 | 17,5 | 1 | 3 | 3 | 3 | 4 | 4 | 2 | 4 | 3 | 2 |
| 07:38 | 17,1 | 2 | 1 | 4 | 2 | 3 | 2 | 2 | 4 | 2 | 2 |
| 07:43 | 17,1 | 3 | 1 | 4 | 3 | 5 | 4 | 2 | 4 | 3 | 2 |
| 07:47 | 15,3 | 2 | 1 | 4 | 5 | 3 | 2 | 1 | 3 | 2 | 1 |
| 07:50 | 16,0 | 4 | 1 | 4 | 3 | 3 | 2 | 1 | 3 | 2 | 1 |
| 07:53 | 16,0 | 4 | 1 | 4 | 2 | 3 | 4 | 1 | 3 | 2 | 1 |
| 07:57 | 16,3 | 3 | 2 | 5 | 4 | 4 | 1 | 1 | 4 | 2 | 1 |
| 08:01 | 17,2 | 4 | 1 | 3 | 4 | 5 | 2 | 1 | 4 | 3 | 2 |
| 08:09 | 17,3 | 4 | 1 | 3 | 4 | 4 | 3 | 1 | 4 | 2 | 2 |
| 08:12 | 21,3 | 4 | 1 | 4 | 4 | 5 | 4 | 1 | 3 | 2 | 1 |
| 08:18 | 22,2 | 4 | 1 | 4 | 3 | 3 | 4 | 1 | 4 | 2 | 1 |
| 08:23 | 20,9 | 4 | 1 | 4 | 3 | 3 | 3 | 1 | 2 | 2 | 1 |
| 08:26 | 22,3 | 4 | 1 | 7 | 1 | 1 | 1 | 2 | 3 | 2 | 1 |
| 08:30 | 26,1 | 2 | 3 | 5 | 2 | 2 | 1 | 2 | 3 | 2 | 1 |
| 08:38 | 24,0 | 2 | 1 | 4 | 4 | 4 | 3 | 1 | 3 | 2 | 1 |
| 08:42 | 24,2 | 2 | 3 | 5 | 1 | 1 | 1 | 1 | 2 | 2 | 1 |
| 08:47 | 23,4 | 3 | 1 | 4 | 1 | 2 | 4 | 2 | 2 | 2 | 1 |
| 08:52 | 24,2 | 3 | 2 | 5 | 3 | 2 | 1 | 2 | 3 | 2 | 1 |
| 08:54 | 24,2 | 2 | 1 | 4 | 2 | 2 | 1 | 2 | 3 | 2 | 1 |
| 08:56 | 24,2 | 2 | 1 | 4 | 2 | 3 | 2 | 2 | 3 | 2 | 1 |
| 08:58 | 24,2 | 2 | 2 | 4 | 2 | 1 | 1 | 2 | 3 | 2 | 1 |
| 09:00 | 25,5 | 4 | 1 | 4 | 2 | 2 | 1 | 1 | 1 | 3 | 1 |
| 09:05 | 27,1 | 4 | 1 | 4 | 3 | 3 | 1 | 1 | 2 | 3 | 1 |
| 09:30 | 24,8 | 4 | 1 | 4 | 2 | 1 | 1 | 1 | 1 | 2 | 1 |
| 09:35 | 25,3 | 4 | 1 | 4 | 3 | 4 | 4 | 1 | 2 | 3 | 1 |
| 09:40 | 27,2 | 2 | 3 | 5 | 3 | 2 | 1 | 2 | 3 | 1 | 1 |
| 09:45 | 26,1 | 2 | 2 | 4 | 2 | 3 | 4 | 2 | 2 | 1 | 1 |
| 09:49 | 26,1 | 5 | 1 | 3 | 2 | 2 | 2 | 1 | 2 | 1 | 1 |
| 09:55 | 25,5 | 4 | 1 | 4 | 4 | 3 | 2 | 1 | 2 | 1 | 1 |
| 10:02 | 18,8 | 4 | 1 | 4 | 4 | 3 | 2 | 1 | 2 | 2 | 1 |
| 10:10 | 20,8 | 4 | 1 | 4 | 3 | 3 | 1 | 1 | 1 | 2 | 1 |
| 10:12 | 20,6 | 3 | 2 | 5 | 2 | 5 | 4 | 2 | 2 | 2 | 2 |
| 10:17 | 19,3 | 3 | 2 | 5 | 2 | 5 | 4 | 2 | 3 | 2 | 2 |
| 10:31 | 21,2 | 3 | 1 | 3 | 3 | 2 | 1 | 2 | 2 | 1 | 1 |
| 10:34 | 21,2 | 5 | 1 | 4 | 2 | 1 | 1 | 2 | 2 | 1 | 1 |
| 10:38 | 21,5 | 4 | 1 | 4 | 2 | 1 | 1 | 2 | 2 | 1 | 1 |
| 10:41 | 20,0 | 3 | 1 | 4 | 4 | 5 | 4 | 1 | 2 | 2 | 1 |
| 10:59 | 20,8 | 2 | 2 | 4 | 2 | 2 | 1 | 2 | 3 | 2 | 2 |
| 11:10 | 26,4 | 4 | 1 | 4 | 4 | 4 | 1 | 1 | 2 | 1 | 1 |
| 11:15 | 24,1 | 4 | 1 | 4 | 3 | 4 | 4 | 2 | 2 | 1 | 1 |
| 11:20 | 27,6 | 6 | 2 | 4 | 3 | 1 | 1 | 2 | 3 | 1 | 1 |
| 11:26 | 27,5 | 4 | 1 | 4 | 2 | 5 | 4 | 2 | 2 | 3 | 1 |
| 11:29 | 21,8 | 3 | 2 | 3 | 3 | 2 | 1 | 2 | 2 | 2 | 2 |
| 11:34 | 28,6 | 4 | 1 | 4 | 3 | 3 | 3 | 1 | 2 | 2 | 1 |
| 11:38 | 25,7 | 5 | 1 | 4 | 4 | 4 | 1 | 1 | 2 | 2 | 1 |
| 11:42 | 25,1 | 2 | 1 | 4 | 4 | 4 | 2 | 1 | 3 | 3 | 1 |
| 11:46 | 30,3 | 4 | 1 | 4 | 2 | 4 | 4 | 2 | 2 | 3 | 1 |
| 11:50 | 19,6 | 4 | 1 | 3 | 2 | 4 | 1 | 2 | 3 | 3 | 1 |
| 11:54 | 19,6 | 4 | 1 | 3 | 3 | 2 | 2 | 1 | 2 | 3 | 1 |
| 11:57 | 26,5 | 4 | 1 | 4 | 3 | 4 | 4 | 1 | 1 | 2 | 1 |
| 07:04 | 16,9 | 2 | 1 | 5 | 3 | 4 | 4 | 1 | 4 | 2 | 2 |
| 07:07 | 16,2 | 3 | 2 | 3 | 3 | 3 | 2 | 1 | 3 | 2 | 2 |
| 07:10 | 16,6 | 2 | 1 | 4 | 4 | 4 | 2 | 1 | 4 | 2 | 2 |
| 07:14 | 16,6 | 1 | 1 | 5 | 4 | 4 | 3 | 1 | 4 | 2 | 2 |
| 07:17 | 17,4 | 1 | 2 | 5 | 3 | 3 | 3 | 2 | 4 | 2 | 2 |
| 07:21 | 16,5 | 2 | 1 | 5 | 4 | 4 | 4 | 1 | 3 | 2 | 2 |
| 07:25 | 16,6 | 1 | 2 | 4 | 5 | 5 | 2 | 1 | 4 | 2 | 2 |
| 07:28 | 16,6 | 1 | 3 | 5 | 3 | 2 | 1 | 2 | 4 | 1 | 2 |
| 07:30 | 17,5 | 2 | 2 | 4 | 2 | 3 | 2 | 2 | 4 | 2 | 2 |
| 07:35 | 17,1 | 1 | 1 | 4 | 4 | 3 | 3 | 1 | 2 | 1 | 2 |
| 07:49 | 17,2 | 1 | 2 | 4 | 3 | 3 | 3 | 2 | 4 | 2 | 2 |
| 07:54 | 18,3 | 4 | 1 | 4 | 4 | 4 | 3 | 1 | 3 | 2 | 2 |
| 08:00 | 17,2 | 1 | 2 | 3 | 4 | 4 | 4 | 1 | 4 | 2 | 2 |
| 08:03 | 17,2 | 2 | 2 | 4 | 4 | 3 | 1 | 1 | 4 | 2 | 2 |
| 08:07 | 17,3 | 1 | 2 | 1 | 3 | 4 | 4 | 2 | 4 | 2 | 2 |
| 08:10 | 18,5 | 2 | 1 | 4 | 4 | 4 | 3 | 1 | 4 | 2 | 2 |
| 08:15 | 17,9 | 3 | 1 | 3 | 3 | 4 | 3 | 1 | 4 | 2 | 2 |
| 08:20 | 18,1 | 4 | 1 | 4 | 4 | 5 | 4 | 1 | 3 | 2 | 2 |
| 08:25 | 16,8 | 2 | 2 | 5 | 3 | 3 | 3 | 2 | 3 | 2 | 2 |
| 08:35 | 17,3 | 2 | 3 | 5 | 3 | 2 | 2 | 2 | 4 | 2 | 2 |
| 08:48 | 23,4 | 3 | 1 | 2 | 4 | 3 | 3 | 1 | 3 | 2 | 1 |
| 08:52 | 24,2 | 3 | 1 | 1 | 4 | 4 | 3 | 1 | 4 | 2 | 1 |
| 08:59 | 24,2 | 1 | 2 | 1 | 4 | 4 | 4 | 1 | 4 | 2 | 1 |
| 09:03 | 25,5 | 4 | 1 | 1 | 3 | 3 | 3 | 2 | 3 | 2 | 1 |
| 09:08 | 27,1 | 3 | 1 | 2 | 4 | 2 | 1 | 2 | 3 | 2 | 1 |
| 09:12 | 23,3 | 4 | 3 | 5 | 4 | 3 | 1 | 1 | 2 | 2 | 1 |
| 09:16 | 26,1 | 2 | 2 | 1 | 3 | 3 | 2 | 2 | 3 | 2 | 1 |
| 09:21 | 19,5 | 3 | 1 | 4 | 4 | 4 | 1 | 1 | 4 | 1 | 2 |
| 09:26 | 27,6 | 3 | 1 | 5 | 5 | 5 | 2 | 1 | 4 | 2 | 1 |
| 09:31 | 24,8 | 4 | 1 | 1 | 3 | 3 | 2 | 1 | 3 | 1 | 1 |
| 09:44 | 27,2 | 4 | 2 | 5 | 4 | 4 | 1 | 1 | 3 | 2 | 1 |
| 09:51 | 25,8 | 5 | 2 | 1 | 3 | 5 | 1 | 1 | 3 | 2 | 1 |
| 09:56 | 25,5 | 3 | 1 | 4 | 4 | 3 | 1 | 1 | 3 | 2 | 1 |
| 10:00 | 18,8 | 1 | 3 | 3 | 4 | 3 | 2 | 2 | 3 | 1 | 1 |
| 10:05 | 20,2 | 4 | 3 | 4 | 3 | 3 | 4 | 2 | 3 | 2 | 1 |
| 10:10 | 20,8 | 3 | 1 | 5 | 4 | 4 | 4 | 2 | 4 | 2 | 1 |
| 10:17 | 21,7 | 4 | 1 | 4 | 3 | 3 | 2 | 2 | 4 | 2 | 1 |
| 10:21 | 20,4 | 4 | 1 | 1 | 4 | 4 | 2 | 1 | 4 | 2 | 1 |
| 10:26 | 21,8 | 2 | 1 | 4 | 3 | 3 | 4 | 2 | 3 | 2 | 1 |
| 10:31 | 21,2 | 1 | 1 | 3 | 4 | 3 | 3 | 1 | 3 | 2 | 1 |
| 10:45 | 21,8 | 3 | 1 | 6 | 2 | 2 | 1 | 2 | 2 | 1 | 1 |
| 10:50 | 19,6 | 7 | 3 | 1 | 3 | 2 | 1 | 2 | 4 | 2 | 1 |
| 10:55 | 24,6 | 3 | 1 | 1 | 5 | 5 | 3 | 1 | 4 | 2 | 1 |
| 11:00 | 20,7 | 3 | 1 | 1 | 4 | 4 | 4 | 2 | 4 | 2 | 2 |
| 11:05 | 26,6 | 3 | 1 | 1 | 4 | 5 | 4 | 1 | 4 | 2 | 1 |
| 11:10 | 26,4 | 4 | 1 | 1 | 4 | 4 | 2 | 1 | 3 | 2 | 1 |
| 11:15 | 24,1 | 6 | 2 | 1 | 3 | 4 | 3 | 1 | 3 | 2 | 1 |
| 11:21 | 27,6 | 5 | 1 | 5 | 3 | 3 | 2 | 1 | 3 | 2 | 1 |
| 11:26 | 27,5 | 3 | 1 | 1 | 3 | 2 | 2 | 2 | 3 | 1 | 1 |
| 11:30 | 28,6 | 5 | 2 | 1 | 2 | 3 | 3 | 2 | 3 | 2 | 1 |
| 11:37 | 22,2 | 3 | 1 | 1 | 3 | 3 | 3 | 2 | 3 | 2 | 2 |
| 11:44 | 25,1 | 4 | 1 | 1 | 3 | 3 | 2 | 2 | 3 | 2 | 1 |
| 11:51 | 19,6 | 4 | 1 | 1 | 3 | 3 | 4 | 2 | 4 | 2 | 1 |
| 12:25 | 28,7 | 7 | 3 | 4 | 4 | 2 | 1 | 1 | 2 | 2 | 1 |
| 12:25 | 28,7 | 4 | 1 | 4 | 3 | 2 | 1 | 1 | 2 | 2 | 1 |
| 12:30 | 27,2 | 4 | 1 | 4 | 3 | 2 | 1 | 1 | 3 | 2 | 1 |
| 12:33 | 27,2 | 3 | 1 | 4 | 3 | 3 | 4 | 1 | 3 | 2 | 1 |
| 12:35 | 24,6 | 4 | 1 | 4 | 2 | 3 | 4 | 2 | 2 | 2 | 1 |
| 12:40 | 20,8 | 4 | 1 | 4 | 3 | 2 | 1 | 2 | 2 | 2 | 1 |
| 12:45 | 22,8 | 4 | 1 | 5 | 3 | 3 | 1 | 1 | 2 | 2 | 1 |
| 12:50 | 28,7 | 5 | 2 | 4 | 3 | 3 | 1 | 1 | 2 | 2 | 1 |
| 12:50 | 28,7 | 4 | 1 | 3 | 2 | 3 | 4 | 2 | 2 | 2 | 1 |
| 12:50 | 28,7 | 4 | 1 | 4 | 3 | 2 | 2 | 2 | 2 | 2 | 1 |
| 13:03 | 29,5 | 5 | 2 | 3 | 99 | 99 | 99 | 2 | 99 | 99 | 1 |
| 13:05 | 30,9 | 3 | 3 | 4 | 3 | 3 | 1 | 1 | 2 | 2 | 1 |
| 13:05 | 30,9 | 4 | 1 | 4 | 2 | 2 | 1 | 2 | 1 | 2 | 1 |
| 13:07 | 30,9 | 6 | 1 | 4 | 4 | 4 | 4 | 1 | 3 | 2 | 1 |
| 13:10 | 20,9 | 4 | 1 | 3 | 3 | 2 | 2 | 2 | 2 | 2 | 1 |
| 13:10 | 20,9 | 4 | 2 | 4 | 3 | 4 | 1 | 1 | 3 | 2 | 1 |
| 13:12 | 20,9 | 4 | 4 | 5 | 2 | 2 | 2 | 2 | 3 | 2 | 1 |
| 13:15 | 26,4 | 4 | 1 | 3 | 4 | 4 | 2 | 1 | 3 | 2 | 1 |
| 13:20 | 31,4 | 4 | 1 | 3 | 3 | 2 | 2 | 2 | 3 | 2 | 1 |
| 13:25 | 28,5 | 4 | 5 | 4 | 4 | 4 | 2 | 1 | 3 | 3 | 1 |
| 13:35 | 29,4 | 4 | 1 | 4 | 4 | 4 | 2 | 2 | 2 | 2 | 1 |
| 13:40 | 26,1 | 4 | 1 | 3 | 4 | 4 | 2 | 1 | 4 | 3 | 1 |
| 13:45 | 24,3 | 4 | 1 | 4 | 4 | 3 | 1 | 1 | 3 | 2 | 2 |
| 13:50 | 23,8 | 4 | 1 | 4 | 3 | 4 | 3 | 1 | 3 | 2 | 2 |
| 14:00 | 24,2 | 4 | 4 | 4 | 3 | 4 | 4 | 1 | 3 | 3 | 2 |
| 14:05 | 24,5 | 4 | 1 | 4 | 3 | 4 | 4 | 1 | 2 | 2 | 2 |
| 14:08 | 24,5 | 4 | 1 | 4 | 3 | 3 | 1 | 1 | 2 | 2 | 2 |
| 14:20 | 23,7 | 4 | 1 | 4 | 4 | 2 | 1 | 1 | 3 | 2 | 2 |
| 14:35 | 24,4 | 4 | 1 | 2 | 3 | 2 | 1 | 1 | 1 | 2 | 2 |
| 14:35 | 24,4 | 4 | 1 | 4 | 3 | 3 | 1 | 2 | 3 | 2 | 2 |
| 14:45 | 22,8 | 5 | 1 | 4 | 4 | 4 | 2 | 1 | 2 | 3 | 2 |
| 14:50 | 21,5 | 5 | 3 | 2 | 4 | 4 | 3 | 1 | 3 | 3 | 2 |
| 14:50 | 21,5 | 2 | 2 | 5 | 4 | 3 | 1 | 1 | 1 | 3 | 2 |
| 15:00 | 22,2 | 4 | 4 | 4 | 4 | 4 | 4 | 1 | 3 | 2 | 2 |
| 15:15 | 22,2 | 4 | 1 | 4 | 3 | 3 | 2 | 2 | 3 | 2 | 2 |
| 15:20 | 22,2 | 4 | 4 | 4 | 3 | 4 | 4 | 1 | 2 | 2 | 2 |
| 15:25 | 22,7 | 4 | 1 | 4 | 4 | 3 | 2 | 1 | 3 | 3 | 2 |
| 15:30 | 24,6 | 4 | 1 | 4 | 3 | 3 | 1 | 2 | 2 | 2 | 2 |
| 15:40 | 23,3 | 4 | 1 | 3 | 2 | 3 | 2 | 2 | 3 | 3 | 2 |
| 15:42 | 23,3 | 4 | 1 | 2 | 3 | 3 | 1 | 1 | 3 | 3 | 2 |
| 15:45 | 23,1 | 4 | 1 | 3 | 2 | 4 | 2 | 2 | 2 | 2 | 2 |
| 16:00 | 23,2 | 4 | 1 | 2 | 4 | 5 | 2 | 1 | 3 | 3 | 2 |
| 16:05 | 23,1 | 4 | 1 | 2 | 3 | 3 | 2 | 1 | 3 | 2 | 2 |
| 16:10 | 22,5 | 4 | 1 | 4 | 4 | 3 | 1 | 1 | 2 | 2 | 2 |
| 16:15 | 23,8 | 4 | 1 | 4 | 3 | 3 | 1 | 2 | 2 | 2 | 2 |
| 16:20 | 22,9 | 4 | 1 | 4 | 3 | 4 | 1 | 2 | 2 | 2 | 2 |
| 16:25 | 21,4 | 3 | 2 | 4 | 3 | 5 | 2 | 1 | 3 | 2 | 2 |
| 16:30 | 19,8 | 4 | 1 | 4 | 3 | 3 | 3 | 2 | 2 | 3 | 2 |
| 16:33 | 19,8 | 4 | 1 | 2 | 3 | 4 | 2 | 1 | 3 | 2 | 2 |
| 16:40 | 23,3 | 4 | 1 | 4 | 2 | 1 | 2 | 1 | 3 | 2 | 2 |
| 16:40 | 23,3 | 4 | 1 | 4 | 4 | 4 | 4 | 1 | 3 | 2 | 2 |
| 16:45 | 21,5 | 1 | 4 | 6 | 4 | 3 | 2 | 2 | 2 | 2 | 2 |
| 16:55 | 21,2 | 2 | 1 | 4 | 3 | 3 | 1 | 1 | 3 | 2 | 2 |
| 16:56 | 21,2 | 2 | 1 | 4 | 3 | 3 | 1 | 1 | 2 | 2 | 2 |
| 12:02 | 21,9 | 6 | 4 | 3 | 3 | 5 | 1 | 2 | 2 | 2 | 2 |
| 12:14 | 23,0 | 4 | 1 | 4 | 5 | 4 | 2 | 1 | 2 | 2 | 2 |
| 12:18 | 21,6 | 4 | 1 | 1 | 5 | 5 | 4 | 1 | 3 | 2 | 2 |
| 12:24 | 23,1 | 2 | 1 | 4 | 3 | 4 | 4 | 2 | 3 | 2 | 2 |
| 12:28 | 21,4 | 6 | 4 | 2 | 3 | 5 | 1 | 2 | 2 | 2 | 2 |
| 12:32 | 22,7 | 6 | 4 | 2 | 2 | 3 | 1 | 2 | 2 | 2 | 2 |
| 12:36 | 22,9 | 6 | 4 | 2 | 2 | 4 | 1 | 2 | 2 | 2 | 2 |
| 12:49 | 23,0 | 4 | 4 | 2 | 3 | 3 | 1 | 2 | 2 | 2 | 2 |
| 12:52 | 24,1 | 2 | 3 | 6 | 5 | 5 | 3 | 1 | 3 | 2 | 2 |
| 12:55 | 23,1 | 4 | 1 | 6 | 3 | 3 | 1 | 1 | 3 | 2 | 2 |
| 13:40 | 25,6 | 2 | 3 | 5 | 1 | 3 | 1 | 1 | 2 | 2 | 2 |
| 13:44 | 26,1 | 2 | 1 | 5 | 4 | 3 | 1 | 2 | 2 | 1 | 1 |
| 13:48 | 24,4 | 4 | 1 | 4 | 4 | 3 | 1 | 1 | 2 | 1 | 1 |
| 13:51 | 23,9 | 4 | 1 | 4 | 5 | 3 | 1 | 1 | 1 | 1 | 1 |
| 13:55 | 25,4 | 2 | 1 | 2 | 5 | 3 | 1 | 2 | 2 | 2 | 1 |
| 13:58 | 23,9 | 4 | 2 | 4 | 3 | 1 | 1 | 2 | 2 | 1 | 2 |
| 14:02 | 24,2 | 4 | 1 | 4 | 4 | 3 | 1 | 1 | 2 | 1 | 2 |
| 14:12 | 23,5 | 6 | 1 | 4 | 4 | 5 | 2 | 1 | 1 | 2 | 2 |
| 14:15 | 24,1 | 4 | 1 | 2 | 2 | 3 | 4 | 2 | 3 | 2 | 2 |
| 14:28 | 24,0 | 2 | 1 | 4 | 4 | 3 | 3 | 1 | 2 | 2 | 2 |
| 13:02 | 22,7 | 4 | 1 | 4 | 4 | 3 | 2 | 1 | 3 | 2 | 2 |
| 13:04 | 22,7 | 2 | 1 | 4 | 3 | 5 | 3 | 1 | 2 | 2 | 2 |
| 13:08 | 21,6 | 4 | 1 | 2 | 3 | 3 | 4 | 2 | 2 | 2 | 2 |
| 13:12 | 22,3 | 6 | 1 | 4 | 3 | 5 | 3 | 1 | 2 | 2 | 2 |
| 13:15 | 26,4 | 6 | 2 | 3 | 3 | 5 | 3 | 2 | 2 | 1 | 1 |
| 13:18 | 22,2 | 2 | 1 | 4 | 3 | 4 | 1 | 2 | 2 | 2 | 2 |
| 13:22 | 23,6 | 2 | 1 | 4 | 4 | 3 | 2 | 2 | 3 | 2 | 2 |
| 13:31 | 23,6 | 4 | 1 | 4 | 3 | 4 | 3 | 1 | 3 | 2 | 2 |
| 13:35 | 24,7 | 4 | 1 | 4 | 4 | 5 | 1 | 1 | 2 | 2 | 2 |
| 13:38 | 29,4 | 2 | 1 | 4 | 4 | 4 | 1 | 1 | 1 | 1 | 1 |
| 14:25 | 24,0 | 2 | 1 | 2 | 4 | 4 | 2 | 1 | 2 | 1 | 2 |
| 14:31 | 23,6 | 4 | 2 | 2 | 4 | 5 | 1 | 1 | 2 | 1 | 2 |
| 14:38 | 24,4 | 2 | 1 | 5 | 4 | 3 | 2 | 1 | 1 | 1 | 2 |
| 14:40 | 23,6 | 2 | 1 | 5 | 5 | 5 | 3 | 1 | 3 | 1 | 2 |
| 14:44 | 23,6 | 2 | 1 | 6 | 2 | 3 | 3 | 2 | 1 | 1 | 2 |
| 14:48 | 22,8 | 2 | 2 | 4 | 4 | 3 | 1 | 1 | 1 | 2 | 2 |
| 14:52 | 21,5 | 4 | 1 | 4 | 4 | 3 | 1 | 1 | 2 | 2 | 2 |
| 15:02 | 22,2 | 4 | 1 | 4 | 4 | 4 | 1 | 1 | 2 | 2 | 2 |
| 15:06 | 23,3 | 4 | 2 | 6 | 3 | 4 | 3 | 1 | 2 | 2 | 2 |
| 15:09 | 23,3 | 4 | 1 | 4 | 2 | 4 | 2 | 2 | 2 | 2 | 2 |
| 15:22 | 22,2 | 4 | 2 | 4 | 3 | 4 | 2 | 2 | 3 | 1 | 2 |
| 15:28 | 22,7 | 2 | 4 | 4 | 3 | 2 | 3 | 2 | 3 | 2 | 2 |
| 15:38 | 24,6 | 2 | 1 | 6 | 4 | 3 | 1 | 2 | 2 | 2 | 2 |
| 15:42 | 23,3 | 2 | 1 | 4 | 2 | 2 | 1 | 2 | 3 | 1 | 2 |
| 15:49 | 23,1 | 2 | 1 | 6 | 3 | 5 | 1 | 2 | 2 | 1 | 2 |
| 15:59 | 24,3 | 2 | 3 | 6 | 3 | 5 | 1 | 2 | 3 | 1 | 2 |
| 16:02 | 23,2 | 2 | 3 | 6 | 5 | 3 | 1 | 2 | 3 | 1 | 2 |
| 16:08 | 23,1 | 2 | 3 | 6 | 3 | 2 | 3 | 2 | 3 | 1 | 2 |
| 16:12 | 22,5 | 2 | 2 | 6 | 2 | 1 | 2 | 2 | 2 | 1 | 2 |
| 16:20 | 22,9 | 2 | 3 | 4 | 3 | 2 | 1 | 2 | 2 | 1 | 2 |
| 16:20 | 22,9 | 4 | 1 | 4 | 4 | 3 | 1 | 1 | 3 | 2 | 2 |
| 16:23 | 22,9 | 2 | 1 | 6 | 4 | 3 | 1 | 2 | 3 | 2 | 2 |
| 16:25 | 21,4 | 4 | 3 | 4 | 4 | 3 | 2 | 1 | 3 | 1 | 2 |
| 16:28 | 21,4 | 4 | 1 | 4 | 2 | 3 | 2 | 2 | 3 | 2 | 2 |
| 16:30 | 19,8 | 2 | 1 | 6 | 3 | 3 | 1 | 1 | 2 | 2 | 2 |
| 16:34 | 19,8 | 2 | 2 | 4 | 5 | 5 | 1 | 1 | 2 | 2 | 2 |
| 16:38 | 21,8 | 2 | 2 | 4 | 4 | 4 | 1 | 1 | 2 | 2 | 2 |
| 16:41 | 23,3 | 4 | 1 | 4 | 4 | 3 | 1 | 1 | 3 | 1 | 2 |
| 16:45 | 21,5 | 4 | 1 | 4 | 3 | 5 | 3 | 1 | 2 | 2 | 2 |
| 16:50 | 21,3 | 3 | 2 | 6 | 3 | 5 | 4 | 1 | 2 | 2 | 2 |
| 14:08 | 24,5 | 5 | 1 | 4 | 2 | 2 | 3 | 1 | 2 | 2 | 2 |
| 07:00 | 16,9 | 4 | 4 | 5 | 4 | 4 | 3 | 2 | 3 | 1 | 2 |
| 07:00 | 16,9 | 2 | 1 | 4 | 3 | 2 | 3 | 2 | 3 | 1 | 2 |
| 07:09 | 16,2 | 2 | 1 | 5 | 2 | 2 | 3 | 2 | 3 | 3 | 2 |
| 07:12 | 16,6 | 2 | 1 | 4 | 4 | 4 | 4 | 1 | 3 | 99 | 2 |
| 07:15 | 17,4 | 4 | 1 | 5 | 3 | 4 | 3 | 1 | 3 | 3 | 2 |
| 07:19 | 17,4 | 3 | 2 | 4 | 4 | 4 | 1 | 2 | 3 | 1 | 2 |
| 07:23 | 16,5 | 2 | 2 | 6 | 3 | 4 | 2 | 2 | 3 | 1 | 2 |
| 07:27 | 16,6 | 4 | 1 | 6 | 3 | 4 | 3 | 1 | 2 | 3 | 2 |
| 07:32 | 17,5 | 4 | 1 | 4 | 2 | 3 | 3 | 2 | 3 | 1 | 2 |
| 07:37 | 17,1 | 3 | 1 | 5 | 4 | 5 | 1 | 2 | 3 | 1 | 2 |
| 08:05 | 17,3 | 2 | 3 | 4 | 3 | 4 | 1 | 1 | 3 | 1 | 2 |
| 08:09 | 17,3 | 3 | 2 | 5 | 2 | 1 | 1 | 2 | 3 | 1 | 2 |
| 08:15 | 17,9 | 3 | 1 | 5 | 4 | 5 | 3 | 1 | 3 | 3 | 2 |
| 08:20 | 18,1 | 2 | 2 | 5 | 3 | 3 | 3 | 2 | 3 | 3 | 2 |
| 08:25 | 16,8 | 4 | 1 | 5 | 3 | 5 | 3 | 1 | 2 | 2 | 2 |
| 08:30 | 18,1 | 3 | 2 | 5 | 5 | 5 | 1 | 1 | 3 | 2 | 2 |
| 08:35 | 17,3 | 4 | 1 | 4 | 4 | 5 | 2 | 1 | 3 | 1 | 2 |
| 08:41 | 17,9 | 2 | 1 | 4 | 4 | 3 | 2 | 1 | 3 | 1 | 2 |
| 08:46 | 16,8 | 4 | 1 | 4 | 3 | 3 | 4 | 1 | 3 | 3 | 2 |
| 08:53 | 17,7 | 4 | 1 | 4 | 4 | 4 | 4 | 1 | 2 | 3 | 2 |
| 09:02 | 18,6 | 3 | 1 | 4 | 4 | 5 | 3 | 1 | 4 | 3 | 2 |
| 09:07 | 17,3 | 4 | 1 | 5 | 4 | 4 | 1 | 1 | 1 | 2 | 2 |
| 09:12 | 18,4 | 2 | 2 | 5 | 4 | 2 | 4 | 1 | 3 | 3 | 2 |
| 09:16 | 19,1 | 3 | 1 | 4 | 4 | 5 | 3 | 1 | 1 | 1 | 2 |
| 09:20 | 19,5 | 4 | 1 | 4 | 5 | 5 | 1 | 1 | 3 | 3 | 2 |
| 09:26 | 18,4 | 3 | 2 | 5 | 3 | 4 | 2 | 2 | 3 | 3 | 2 |
| 09:30 | 19,2 | 2 | 1 | 4 | 2 | 1 | 1 | 2 | 3 | 1 | 2 |
| 09:36 | 18,1 | 2 | 3 | 3 | 2 | 2 | 3 | 1 | 3 | 3 | 2 |
| 09:41 | 18 | 4 | 1 | 4 | 5 | 5 | 3 | 1 | 3 | 3 | 2 |
| 09:30 | 19,2 | 4 | 1 | 4 | 4 | 5 | 4 | 1 | 2 | 3 | 2 |
| 10:02 | 20,6 | 4 | 1 | 4 | 4 | 5 | 3 | 1 | 4 | 1 | 2 |
| 10:07 | 22,2 | 4 | 1 | 2 | 3 | 4 | 2 | 1 | 3 | 2 | 2 |
| 10:13 | 20,6 | 2 | 1 | 4 | 4 | 5 | 2 | 2 | 3 | 1 | 2 |
| 10:20 | 20,9 | 2 | 1 | 5 | 5 | 4 | 2 | 1 | 2 | 3 | 2 |
| 10:26 | 19,8 | 2 | 1 | 5 | 2 | 2 | 4 | 2 | 3 | 3 | 2 |
| 10:31 | 20,4 | 5 | 1 | 4 | 4 | 4 | 1 | 1 | 2 | 3 | 2 |
| 10:35 | 21,1 | 2 | 1 | 2 | 3 | 4 | 4 | 2 | 3 | 3 | 2 |
| 10:46 | 20,8 | 4 | 1 | 4 | 4 | 5 | 1 | 2 | 3 | 1 | 2 |
| 10:55 | 20,8 | 4 | 1 | 4 | 1 | 4 | 2 | 1 | 2 | 2 | 2 |
| 10:56 | 20,8 | 3 | 3 | 5 | 5 | 2 | 2 | 1 | 3 | 2 | 2 |
| 11:00 | 20,7 | 4 | 1 | 4 | 3 | 5 | 2 | 2 | 2 | 3 | 2 |
| 11:04 | 20,7 | 4 | 1 | 4 | 5 | 5 | 3 | 1 | 2 | 3 | 2 |
| 11:09 | 21,7 | 4 | 1 | 4 | 4 | 4 | 4 | 1 | 2 | 3 | 2 |
| 11:13 | 20,6 | 2 | 1 | 4 | 3 | 4 | 3 | 2 | 3 | 2 | 2 |
| 11:19 | 21,0 | 4 | 1 | 4 | 4 | 4 | 2 | 1 | 2 | 3 | 2 |
| 11:22 | 21,2 | 4 | 1 | 4 | 4 | 5 | 2 | 1 | 2 | 3 | 2 |
| 11:30 | 21,3 | 1 | 2 | 4 | 4 | 5 | 2 | 1 | 3 | 3 | 2 |
| 11:35 | 22,2 | 3 | 2 | 5 | 3 | 2 | 2 | 2 | 3 | 1 | 2 |
| 11:40 | 23,5 | 2 | 3 | 5 | 2 | 3 | 1 | 2 | 2 | 3 | 2 |
| 11:44 | 23,5 | 2 | 1 | 4 | 3 | 5 | 4 | 1 | 2 | 2 | 2 |
| 11:49 | 22,0 | 3 | 1 | 4 | 2 | 2 | 3 | 1 | 2 | 1 | 2 |
| 11:53 | 23,4 | 2 | 2 | 5 | 5 | 5 | 1 | 1 | 3 | 3 | 2 |
| 11:58 | 22,5 | 2 | 3 | 5 | 3 | 3 | 1 | 2 | 3 | 3 | 2 |
| 07:04 | 16,9 | 4 | 1 | 5 | 2 | 3 | 2 | 2 | 3 | 1 | 2 |
| 07:08 | 16,2 | 2 | 1 | 4 | 3 | 3 | 3 | 1 | 4 | 1 | 2 |
| 07:10 | 16,6 | 3 | 2 | 4 | 3 | 2 | 1 | 1 | 3 | 2 | 2 |
| 07:10 | 16,6 | 4 | 1 | 4 | 2 | 2 | 3 | 1 | 3 | 2 | 2 |
| 07:20 | 16,5 | 1 | 4 | 6 | 2 | 3 | 3 | 2 | 3 | 2 | 2 |
| 07:23 | 16,5 | 1 | 4 | 6 | 3 | 4 | 3 | 1 | 3 | 3 | 2 |
| 07:26 | 16,6 | 2 | 1 | 4 | 3 | 3 | 4 | 1 | 3 | 3 | 2 |
| 07:30 | 17,5 | 4 | 1 | 4 | 4 | 5 | 3 | 1 | 4 | 3 | 2 |
| 07:34 | 17,5 | 2 | 1 | 5 | 3 | 3 | 2 | 1 | 3 | 3 | 2 |
| 07:40 | 17,1 | 4 | 1 | 4 | 3 | 3 | 1 | 1 | 3 | 3 | 2 |
| 08:20 | 18,1 | 1 | 1 | 3 | 4 | 3 | 2 | 2 | 3 | 2 | 2 |
| 08:25 | 16,8 | 2 | 1 | 5 | 3 | 2 | 2 | 2 | 3 | 2 | 2 |
| 08:28 | 16,8 | 3 | 1 | 3 | 3 | 3 | 2 | 1 | 2 | 1 | 2 |
| 08:32 | 18,1 | 3 | 1 | 4 | 3 | 2 | 3 | 2 | 3 | 1 | 2 |
| 08:32 | 18,1 | 4 | 1 | 4 | 4 | 4 | 3 | 1 | 3 | 2 | 2 |
| 08:35 | 17,3 | 2 | 1 | 3 | 4 | 3 | 3 | 2 | 3 | 2 | 2 |
| 08:40 | 17,9 | 3 | 1 | 5 | 4 | 3 | 1 | 1 | 2 | 1 | 2 |
| 08:45 | 16,8 | 4 | 1 | 5 | 3 | 2 | 1 | 2 | 2 | 2 | 2 |
| 08:45 | 16,8 | 4 | 4 | 3 | 3 | 3 | 1 | 1 | 3 | 2 | 2 |
| 08:50 | 17,7 | 2 | 2 | 5 | 3 | 3 | 2 | 1 | 3 | 3 | 2 |
| 07:41 | 17,1 | 3 | 1 | 7 | 3 | 3 | 1 | 1 | 3 | 1 | 2 |
| 07:45 | 17,2 | 4 | 1 | 4 | 4 | 4 | 2 | 1 | 3 | 2 | 2 |
| 07:50 | 18,3 | 4 | 1 | 4 | 4 | 3 | 3 | 1 | 3 | 2 | 2 |
| 07:50 | 18,3 | 4 | 1 | 6 | 4 | 5 | 3 | 1 | 3 | 2 | 2 |
| 07:58 | 17,6 | 4 | 1 | 4 | 3 | 3 | 1 | 1 | 3 | 2 | 2 |
| 08:00 | 17,2 | 1 | 4 | 6 | 2 | 2 | 3 | 2 | 2 | 2 | 2 |
| 08:05 | 17,3 | 4 | 1 | 4 | 3 | 3 | 1 | 1 | 3 | 2 | 2 |
| 08:08 | 17,3 | 2 | 2 | 5 | 4 | 5 | 3 | 1 | 2 | 3 | 2 |
| 08:14 | 18,5 | 2 | 2 | 5 | 3 | 3 | 2 | 2 | 3 | 2 | 2 |
| 08:20 | 18,1 | 4 | 1 | 4 | 4 | 4 | 2 | 1 | 3 | 3 | 2 |
| 08:55 | 17,1 | 4 | 2 | 3 | 2 | 4 | 3 | 1 | 3 | 2 | 2 |
| 08:55 | 17,1 | 4 | 2 | 3 | 3 | 4 | 3 | 1 | 3 | 2 | 2 |
| 09:00 | 18,6 | 4 | 1 | 5 | 2 | 2 | 4 | 1 | 2 | 2 | 2 |
| 09:05 | 17,3 | 4 | 1 | 3 | 3 | 3 | 2 | 1 | 3 | 3 | 2 |
| 09:10 | 18,4 | 7 | 1 | 4 | 4 | 5 | 4 | 1 | 3 | 3 | 2 |
| 09:15 | 19,1 | 4 | 1 | 4 | 3 | 2 | 4 | 2 | 3 | 2 | 2 |
| 09:20 | 19,5 | 4 | 1 | 4 | 4 | 3 | 2 | 1 | 3 | 2 | 2 |
| 09:30 | 19,2 | 2 | 2 | 5 | 1 | 1 | 1 | 2 | 3 | 2 | 2 |
| 09:40 | 18 | 3 | 2 | 5 | 3 | 3 | 2 | 1 | 3 | 2 | 2 |
| 09:40 | 18 | 1 | 4 | 5 | 3 | 3 | 2 | 1 | 3 | 2 | 2 |
| 09:45 | 19,1 | 4 | 1 | 4 | 3 | 2 | 1 | 1 | 3 | 1 | 2 |
| 09:45 | 19,1 | 4 | 1 | 4 | 3 | 3 | 1 | 2 | 3 | 1 | 2 |
| 09:50 | 19,3 | 4 | 1 | 3 | 2 | 4 | 1 | 2 | 3 | 1 | 2 |
| 09:50 | 19,3 | 4 | 1 | 4 | 4 | 4 | 2 | 1 | 3 | 2 | 2 |
| 09:55 | 19,0 | 1 | 2 | 5 | 3 | 5 | 1 | 2 | 3 | 2 | 2 |
| 10:05 | 22,2 | 4 | 1 | 4 | 3 | 3 | 3 | 1 | 2 | 2 | 2 |
| 10:08 | 22,2 | 1 | 1 | 2 | 3 | 3 | 3 | 1 | 2 | 3 | 2 |
| 10:10 | 20,6 | 3 | 1 | 3 | 3 | 3 | 3 | 1 | 3 | 2 | 2 |
| 10:15 | 19,3 | 2 | 2 | 2 | 3 | 2 | 4 | 1 | 3 | 2 | 2 |
| 10:20 | 20,9 | 4 | 1 | 2 | 3 | 3 | 1 | 1 | 2 | 1 | 2 |
| 10:30 | 20,4 | 4 | 1 | 4 | 3 | 2 | 1 | 2 | 2 | 1 | 2 |
| 10:35 | 21,1 | 4 | 1 | 4 | 3 | 3 | 3 | 2 | 3 | 2 | 2 |
| 10:35 | 21,1 | 5 | 2 | 3 | 3 | 2 | 2 | 2 | 3 | 2 | 2 |
| 10:45 | 20,8 | 4 | 1 | 4 | 4 | 4 | 3 | 1 | 3 | 2 | 2 |
| 10:45 | 20,8 | 4 | 1 | 4 | 4 | 4 | 3 | 1 | 3 | 2 | 2 |
| 10:50 | 20,5 | 4 | 2 | 4 | 3 | 4 | 4 | 1 | 3 | 2 | 2 |
| 10:50 | 20,5 | 4 | 1 | 4 | 3 | 2 | 4 | 2 | 3 | 2 | 2 |
| 11:00 | 20,7 | 4 | 1 | 4 | 4 | 3 | 1 | 1 | 1 | 5 | 2 |
| 11:05 | 21,7 | 4 | 1 | 4 | 2 | 2 | 3 | 2 | 3 | 2 | 2 |
| 11:10 | 20,6 | 5 | 1 | 5 | 2 | 3 | 3 | 2 | 3 | 2 | 2 |
| 11:45 | 22,0 | 3 | 3 | 4 | 3 | 5 | 2 | 2 | 2 | 3 | 2 |
| 11:57 | 22,5 | 5 | 2 | 2 | 5 | 4 | 1 | 1 | 3 | 2 | 2 |
| 12:06 | 22,2 | 4 | 1 | 5 | 4 | 4 | 4 | 1 | 4 | 2 | 2 |
| 12:11 | 23,0 | 4 | 1 | 4 | 4 | 5 | 2 | 2 | 2 | 2 | 2 |
| 12:19 | 21,6 | 4 | 1 | 4 | 4 | 4 | 4 | 1 | 2 | 2 | 2 |
| 12:26 | 21,4 | 3 | 2 | 4 | 2 | 2 | 1 | 2 | 3 | 3 | 2 |
| 12:32 | 22,7 | 3 | 1 | 4 | 3 | 4 | 1 | 2 | 3 | 2 | 2 |
| 12:39 | 22,9 | 4 | 1 | 4 | 3 | 4 | 4 | 2 | 3 | 3 | 2 |
| 12:44 | 22,6 | 4 | 1 | 6 | 2 | 5 | 4 | 1 | 1 | 3 | 2 |
| 12:51 | 24,1 | 6 | 1 | 4 | 4 | 3 | 4 | 1 | 2 | 3 | 2 |
| 12:59 | 23,1 | 4 | 2 | 3 | 4 | 4 | 2 | 1 | 1 | 3 | 2 |
| 13:17 | 22,2 | 3 | 1 | 5 | 4 | 4 | 4 | 1 | 3 | 3 | 2 |
| 13:21 | 23,6 | 3 | 1 | 3 | 3 | 4 | 1 | 1 | 4 | 2 | 2 |
| 13:25 | 22,4 | 3 | 1 | 4 | 1 | 3 | 3 | 2 | 2 | 3 | 2 |
| 13:30 | 23,6 | 3 | 1 | 4 | 3 | 3 | 3 | 1 | 4 | 3 | 2 |
| 13:34 | 23,6 | 4 | 1 | 4 | 4 | 4 | 3 | 1 | 4 | 2 | 2 |
| 13:43 | 25,6 | 4 | 1 | 3 | 4 | 4 | 2 | 1 | 3 | 3 | 2 |
| 13:49 | 24,3 | 4 | 1 | 4 | 3 | 5 | 4 | 1 | 2 | 1 | 2 |
| 13:53 | 23,8 | 4 | 1 | 4 | 2 | 3 | 3 | 2 | 3 | 3 | 2 |
| 13:57 | 23,9 | 4 | 1 | 4 | 3 | 5 | 4 | 2 | 2 | 2 | 2 |
| 14:03 | 24,2 | 5 | 1 | 5 | 2 | 5 | 2 | 2 | 1 | 3 | 2 |
| 14:08 | 24,5 | 4 | 1 | 4 | 2 | 4 | 3 | 1 | 4 | 3 | 2 |
| 14:11 | 23,5 | 3 | 1 | 4 | 2 | 2 | 1 | 2 | 4 | 2 | 2 |
| 14:18 | 24,1 | 4 | 1 | 4 | 3 | 4 | 3 | 1 | 3 | 3 | 2 |
| 14:23 | 23,7 | 4 | 1 | 4 | 3 | 5 | 3 | 2 | 3 | 3 | 2 |
| 14:27 | 24,0 | 4 | 2 | 4 | 3 | 5 | 4 | 1 | 3 | 2 | 2 |
| 14:30 | 23,6 | 4 | 1 | 4 | 4 | 5 | 1 | 2 | 2 | 2 | 2 |
| 14:37 | 24,4 | 4 | 1 | 5 | 3 | 4 | 2 | 2 | 1 | 3 | 2 |
| 14:42 | 23,6 | 4 | 1 | 4 | 4 | 5 | 4 | 1 | 2 | 3 | 2 |
| 14:46 | 22,8 | 4 | 1 | 3 | 5 | 5 | 1 | 1 | 2 | 3 | 2 |
| 14:51 | 21,5 | 5 | 1 | 4 | 2 | 5 | 2 | 2 | 3 | 3 | 2 |
| 14:57 | 22,5 | 4 | 1 | 4 | 3 | 5 | 3 | 2 | 1 | 3 | 2 |
| 15:02 | 22,2 | 3 | 1 | 2 | 4 | 5 | 1 | 1 | 1 | 2 | 2 |
| 15:08 | 23,3 | 4 | 2 | 4 | 3 | 4 | 3 | 2 | 2 | 3 | 2 |
| 15:14 | 24,6 | 4 | 1 | 4 | 5 | 5 | 3 | 2 | 2 | 3 | 2 |
| 15:18 | 22,2 | 2 | 2 | 5 | 5 | 5 | 1 | 2 | 3 | 3 | 2 |
| 15:32 | 24,6 | 4 | 1 | 3 | 5 | 4 | 1 | 1 | 2 | 3 | 2 |
| 15:35 | 24,6 | 4 | 1 | 4 | 5 | 5 | 3 | 2 | 2 | 3 | 2 |
| 15:40 | 23,3 | 4 | 1 | 4 | 2 | 3 | 3 | 2 | 2 | 3 | 2 |
| 15:48 | 23,1 | 4 | 2 | 2 | 2 | 4 | 4 | 2 | 1 | 3 | 2 |
| 15:55 | 24,3 | 1 | 2 | 4 | 4 | 4 | 1 | 1 | 2 | 3 | 2 |
| 15:59 | 24,3 | 2 | 1 | 5 | 4 | 4 | 1 | 1 | 2 | 2 | 2 |
| 16:03 | 23,2 | 4 | 1 | 4 | 5 | 4 | 4 | 1 | 2 | 3 | 2 |
| 16:07 | 23,1 | 4 | 1 | 4 | 5 | 5 | 3 | 1 | 3 | 2 | 2 |
| 16:14 | 22,5 | 4 | 2 | 3 | 5 | 4 | 1 | 1 | 2 | 3 | 2 |
| 16:20 | 22,9 | 5 | 3 | 5 | 3 | 2 | 1 | 2 | 3 | 3 | 2 |
| 16:25 | 21,4 | 4 | 1 | 4 | 4 | 5 | 4 | 1 | 3 | 2 | 2 |
| 16:32 | 19,8 | 4 | 1 | 4 | 4 | 4 | 4 | 1 | 2 | 3 | 2 |
| 16:36 | 21,8 | 6 | 4 | 3 | 2 | 4 | 2 | 1 | 2 | 2 | 2 |
| 16:40 | 23,3 | 4 | 1 | 5 | 2 | 3 | 1 | 2 | 2 | 3 | 2 |
| 16:44 | 23,3 | 3 | 2 | 4 | 3 | 5 | 3 | 2 | 1 | 3 | 2 |
| 16:53 | 21,3 | 3 | 2 | 4 | 5 | 5 | 3 | 1 | 2 | 1 | 2 |
| 16:56 | 21,2 | 4 | 1 | 3 | 5 | 5 | 2 | 1 | 2 | 3 | 2 |
| 11:53 | 23,4 | 4 | 1 | 2 | 3 | 3 | 1 | 1 | 4 | 3 | 2 |
| 11:56 | 22,5 | 4 | 3 | 4 | 3 | 2 | 2 | 2 | 1 | 3 | 2 |
| 12:01 | 21,9 | 2 | 2 | 5 | 2 | 3 | 2 | 2 | 5 | 3 | 2 |
| 12:06 | 22,2 | 4 | 1 | 4 | 2 | 4 | 4 | 1 | 3 | 3 | 2 |
| 12:11 | 23,0 | 4 | 1 | 5 | 1 | 3 | 4 | 2 | 1 | 3 | 2 |
| 12:14 | 23,0 | 5 | 1 | 4 | 3 | 3 | 3 | 99 | 3 | 3 | 2 |
| 12:18 | 21,6 | 3 | 1 | 5 | 3 | 3 | 3 | 1 | 3 | 3 | 2 |
| 12:26 | 21,4 | 4 | 1 | 4 | 3 | 2 | 4 | 1 | 3 | 2 | 2 |
| 12:30 | 22,7 | 6 | 2 | 4 | 4 | 2 | 2 | 1 | 3 | 3 | 2 |
| 12:35 | 22,9 | 5 | 2 | 4 | 4 | 3 | 2 | 1 | 4 | 3 | 2 |
| 12:43 | 22,6 | 3 | 1 | 4 | 2 | 2 | 2 | 2 | 4 | 3 | 2 |
| 12:47 | 23,0 | 4 | 1 | 4 | 3 | 2 | 4 | 1 | 3 | 1 | 2 |
| 12:50 | 24,1 | 4 | 1 | 4 | 2 | 4 | 3 | 1 | 3 | 3 | 2 |
| 12:53 | 24,1 | 4 | 1 | 2 | 3 | 5 | 3 | 1 | 3 | 3 | 2 |
| 12:58 | 23,1 | 2 | 1 | 5 | 2 | 1 | 4 | 2 | 4 | 3 | 2 |
| 13:04 | 22,7 | 4 | 1 | 4 | 2 | 3 | 3 | 2 | 3 | 3 | 2 |
| 13:08 | 21,6 | 4 | 1 | 5 | 4 | 3 | 2 | 1 | 4 | 3 | 2 |
| 13:13 | 22,3 | 4 | 1 | 3 | 4 | 2 | 2 | 1 | 3 | 3 | 2 |
| 13:17 | 22,2 | 4 | 1 | 3 | 3 | 3 | 3 | 1 | 4 | 3 | 2 |
| 13:20 | 23,6 | 4 | 2 | 4 | 3 | 2 | 2 | 2 | 3 | 3 | 2 |
| 13:27 | 22,4 | 3 | 1 | 4 | 2 | 4 | 4 | 1 | 3 | 3 | 2 |
| 13:30 | 23,6 | 4 | 1 | 4 | 3 | 3 | 3 | 1 | 3 | 3 | 2 |
| 13:34 | 23,6 | 4 | 1 | 5 | 3 | 2 | 3 | 2 | 3 | 3 | 2 |
| 13:39 | 24,7 | 5 | 1 | 6 | 3 | 3 | 2 | 1 | 3 | 1 | 2 |
| 13:42 | 25,6 | 4 | 1 | 4 | 2 | 2 | 2 | 99 | 5 | 99 | 2 |
| 13:47 | 24,3 | 4 | 1 | 4 | 3 | 4 | 3 | 1 | 3 | 2 | 2 |
| 13:51 | 23,8 | 4 | 1 | 4 | 3 | 3 | 2 | 2 | 1 | 2 | 2 |
| 13:55 | 23,9 | 4 | 1 | 4 | 2 | 3 | 4 | 1 | 4 | 3 | 2 |
| 13:59 | 23,9 | 4 | 3 | 3 | 4 | 2 | 4 | 1 | 3 | 3 | 2 |
| 14:12 | 23,5 | 7 | 1 | 4 | 3 | 3 | 3 | 1 | 3 | 3 | 2 |
| 14:29 | 24,0 | 4 | 1 | 4 | 4 | 2 | 4 | 1 | 3 | 2 | 2 |
| 14:32 | 23,6 | 4 | 1 | 5 | 3 | 4 | 4 | 1 | 3 | 2 | 2 |
| 14:37 | 24,4 | 4 | 1 | 5 | 3 | 3 | 4 | 99 | 4 | 99 | 2 |
| 14:39 | 24,4 | 4 | 1 | 3 | 4 | 2 | 4 | 1 | 4 | 3 | 2 |
| 14:43 | 23,6 | 4 | 1 | 4 | 3 | 2 | 4 | 1 | 4 | 3 | 2 |
| 14:47 | 22,8 | 4 | 1 | 4 | 3 | 3 | 4 | 1 | 3 | 3 | 2 |
| 14:51 | 21,5 | 4 | 1 | 4 | 3 | 3 | 4 | 1 | 4 | 3 | 2 |
| 14:53 | 21,5 | 4 | 1 | 5 | 5 | 3 | 4 | 1 | 3 | 3 | 2 |
| 14:58 | 22,5 | 4 | 3 | 4 | 2 | 3 | 4 | 2 | 3 | 3 | 2 |
| 15:07 | 23,3 | 4 | 1 | 4 | 3 | 4 | 2 | 1 | 5 | 2 | 2 |
| 15:04 | 22,2 | 4 | 1 | 4 | 4 | 3 | 3 | 1 | 2 | 3 | 2 |
| 15:10 | 24,6 | 4 | 1 | 6 | 3 | 4 | 4 | 1 | 5 | 3 | 2 |
| 15:20 | 22,2 | 4 | 1 | 4 | 2 | 4 | 3 | 1 | 5 | 3 | 2 |
| 15:23 | 22,2 | 3 | 1 | 3 | 3 | 2 | 4 | 2 | 4 | 3 | 2 |
| 15:29 | 22,7 | 3 | 2 | 4 | 4 | 3 | 4 | 2 | 3 | 3 | 2 |
| 15:39 | 24,6 | 3 | 2 | 4 | 4 | 2 | 3 | 2 | 3 | 3 | 2 |
| 15:43 | 23,3 | 3 | 1 | 4 | 2 | 5 | 4 | 2 | 5 | 3 | 2 |
| 15:50 | 23,3 | 4 | 1 | 4 | 3 | 4 | 4 | 1 | 3 | 3 | 2 |
| 15:53 | 23,3 | 4 | 1 | 4 | 3 | 4 | 4 | 1 | 3 | 3 | 2 |
| 15:57 | 24,3 | 6 | 2 | 3 | 2 | 4 | 4 | 2 | 3 | 3 | 2 |
| 16:02 | 23,2 | 3 | 1 | 4 | 3 | 4 | 3 | 1 | 5 | 3 | 2 |
| 16:07 | 23,1 | 4 | 1 | 4 | 3 | 3 | 4 | 1 | 3 | 3 | 2 |
| 16:11 | 22,5 | 4 | 1 | 4 | 2 | 4 | 4 | 1 | 5 | 3 | 2 |
| 16:14 | 22,5 | 2 | 2 | 4 | 3 | 3 | 3 | 1 | 3 | 3 | 2 |
| 16:19 | 23,8 | 3 | 1 | 7 | 2 | 4 | 2 | 1 | 3 | 3 | 2 |
| 16:24 | 22,9 | 5 | 4 | 3 | 3 | 4 | 4 | 1 | 3 | 3 | 2 |
| 16:30 | 19,8 | 4 | 1 | 4 | 3 | 2 | 1 | 2 | 4 | 3 | 2 |
| 16:39 | 21,8 | 1 | 2 | 5 | 3 | 2 | 2 | 1 | 5 | 3 | 2 |
| 16:47 | 21,5 | 4 | 1 | 4 | 4 | 3 | 2 | 1 | 2 | 3 | 2 |
| 16:56 | 21,2 | 4 | 1 | 4 | 3 | 3 | 2 | 1 | 3 | 3 | 2 |
| 09:41 | 18 | 3 | 2 | 4 | 3 | 2 | 2 | 2 | 3 | 2 | 2 |
| 10:05 | 22,2 | 2 | 1 | 6 | 4 | 3 | 1 | 2 | 4 | 3 | 2 |
| 10:05 | 22,2 | 6 | 1 | 4 | 4 | 4 | 1 | 1 | 3 | 3 | 2 |
| 10:14 | 20,6 | 4 | 1 | 4 | 5 | 5 | 4 | 1 | 4 | 2 | 2 |
| 11:07 | 21,7 | 4 | 2 | 4 | 3 | 4 | 2 | 1 | 2 | 2 | 2 |
| 11:07 | 21,7 | 3 | 1 | 4 | 3 | 3 | 1 | 2 | 2 | 2 | 2 |
| 11:07 | 21,7 | 3 | 1 | 4 | 4 | 4 | 4 | 1 | 2 | 2 | 2 |
| 11:55 | 22,5 | 4 | 1 | 4 | 4 | 4 | 4 | 1 | 3 | 3 | 2 |
| 12:10 | 23,0 | 4 | 2 | 5 | 4 | 4 | 1 | 1 | 2 | 2 | 2 |
| 13:35 | 24,7 | 4 | 1 | 4 | 1 | 1 | 2 | 2 | 2 | 1 | 2 |
| 14:32 | 23,6 | 4 | 4 | 4 | 4 | 4 | 2 | 1 | 2 | 3 | 2 |
| 14:32 | 23,6 | 4 | 1 | 4 | 4 | 4 | 2 | 1 | 2 | 3 | 2 |
| 14:40 | 23,6 | 4 | 1 | 3 | 5 | 4 | 2 | 1 | 2 | 2 | 2 |
| 15:04 | 22,2 | 4 | 2 | 5 | 3 | 2 | 1 | 2 | 2 | 3 | 2 |
| 11:30 | 21,3 | 4 | 1 | 1 | 3 | 2 | 1 | 1 | 2 | 3 | 2 |
| 11:35 | 22,2 | 4 | 1 | 3 | 4 | 5 | 3 | 1 | 2 | 3 | 2 |
| 11:43 | 23,5 | 3 | 1 | 5 | 3 | 3 | 1 | 1 | 1 | 1 | 2 |
| 11:50 | 23,4 | 4 | 1 | 4 | 3 | 3 | 2 | 1 | 2 | 3 | 2 |
